# Supplementary figures and images for: The Trypanosoma brucei MISP family of invariant proteins is co-expressed with BARP as triple helical bundle structures on the surface of salivary gland forms, but is dispensable for parasite development within the tsetse vector
Source: PLoS Pathog. 2023 Mar 30;19(3):e1011269. doi: 10.1371/journal.ppat.1011269 (PMC10089363; doi:10.1371/journal.ppat.1011269)

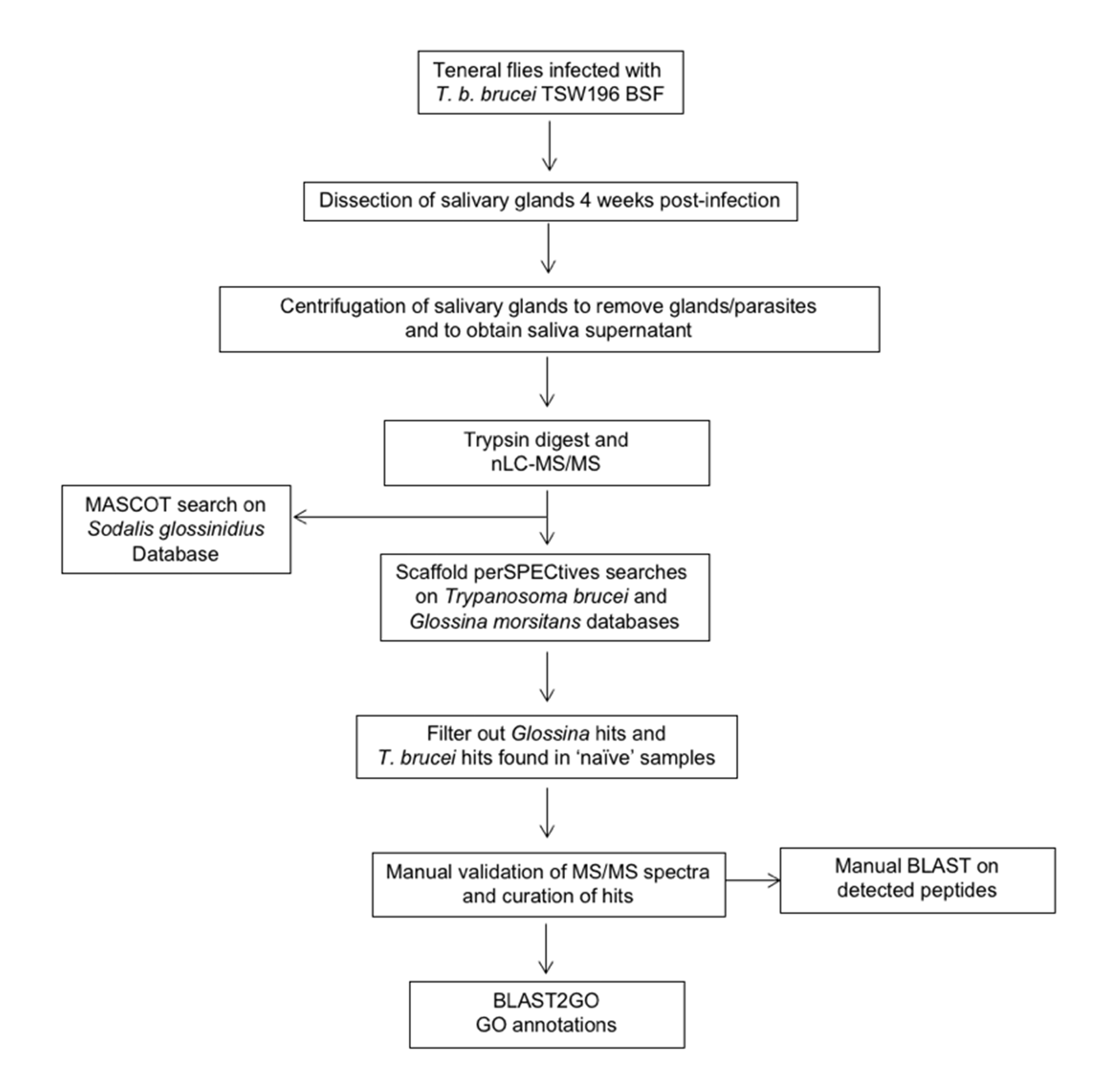

Supplement: S1 Fig — For each biological replicate, saliva samples from naïve flies were also collected and processed in parallel. (TIF) [file ppat.1011269.s001.tif]

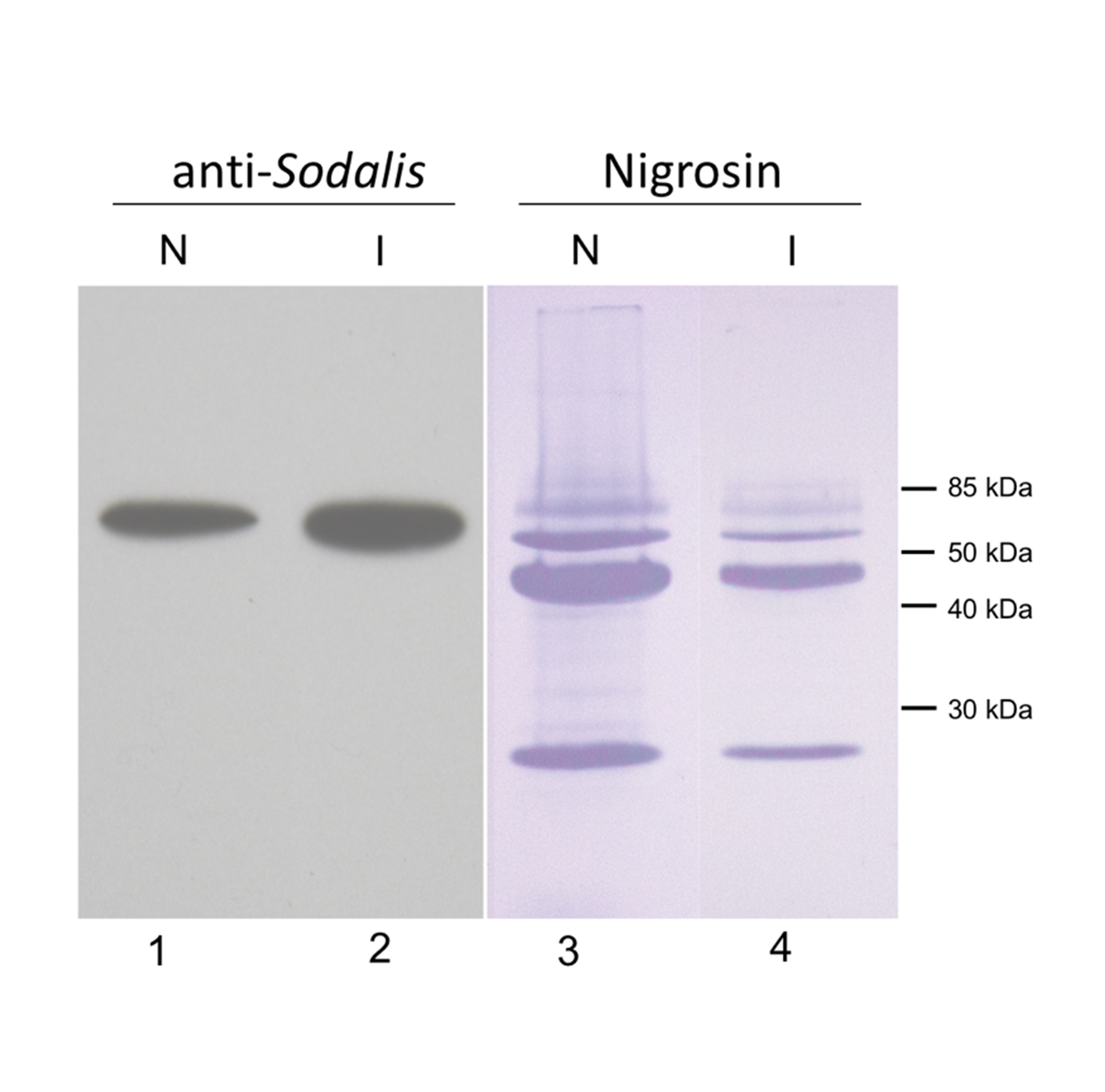

Supplement: S2 Fig — Mouse anti-Sodalis monoclonal antibody 1H1 recognizes a heat shock protein (Hsp60) produced by Sodalis glossinidius. Lanes 1 and 2 represent film exposed for 10 seconds. Lanes 3 and 4 show the nigrosine-stained PVDF membrane used for blotting to visualize the protein loading in each lane. Naïve saliva (N). Infected saliva (I). (TIF) [file ppat.1011269.s002.tif]

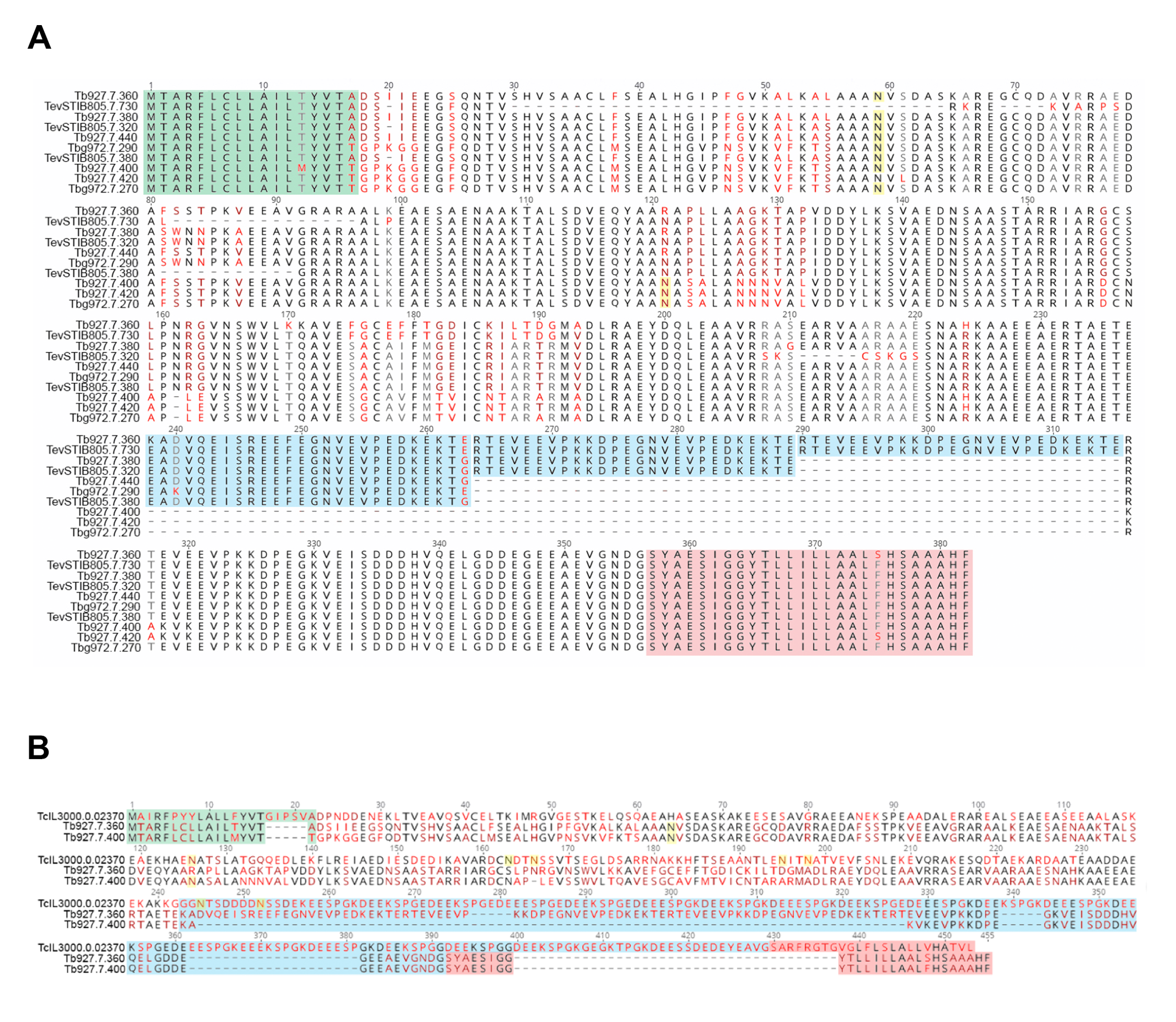

Supplement: S3 Fig — A, Multiple protein alignment of all MISPs (T. congolense MISP excluded). Residues colored based on identity conservation from black (maximum) to red (minimum). Alignment made using a Clustal Omega BLOSUM62 matrix with a gap open cost 10 and a gap extension cost of 0.1, using Geneious R9. Domains highlighted: ER signal peptide (green), potential N-glycosylation sites (yellow asparagines), C-terminus motifs (blue), and GPI-anchor attachment signal peptide (red). B, Multiple protein alignment of T. congolense MISP with MISP360 (MISP-A) and MISP400 (MISP-B). Domains highlighted as in ‘A’. (TIF) [file ppat.1011269.s003.tif]

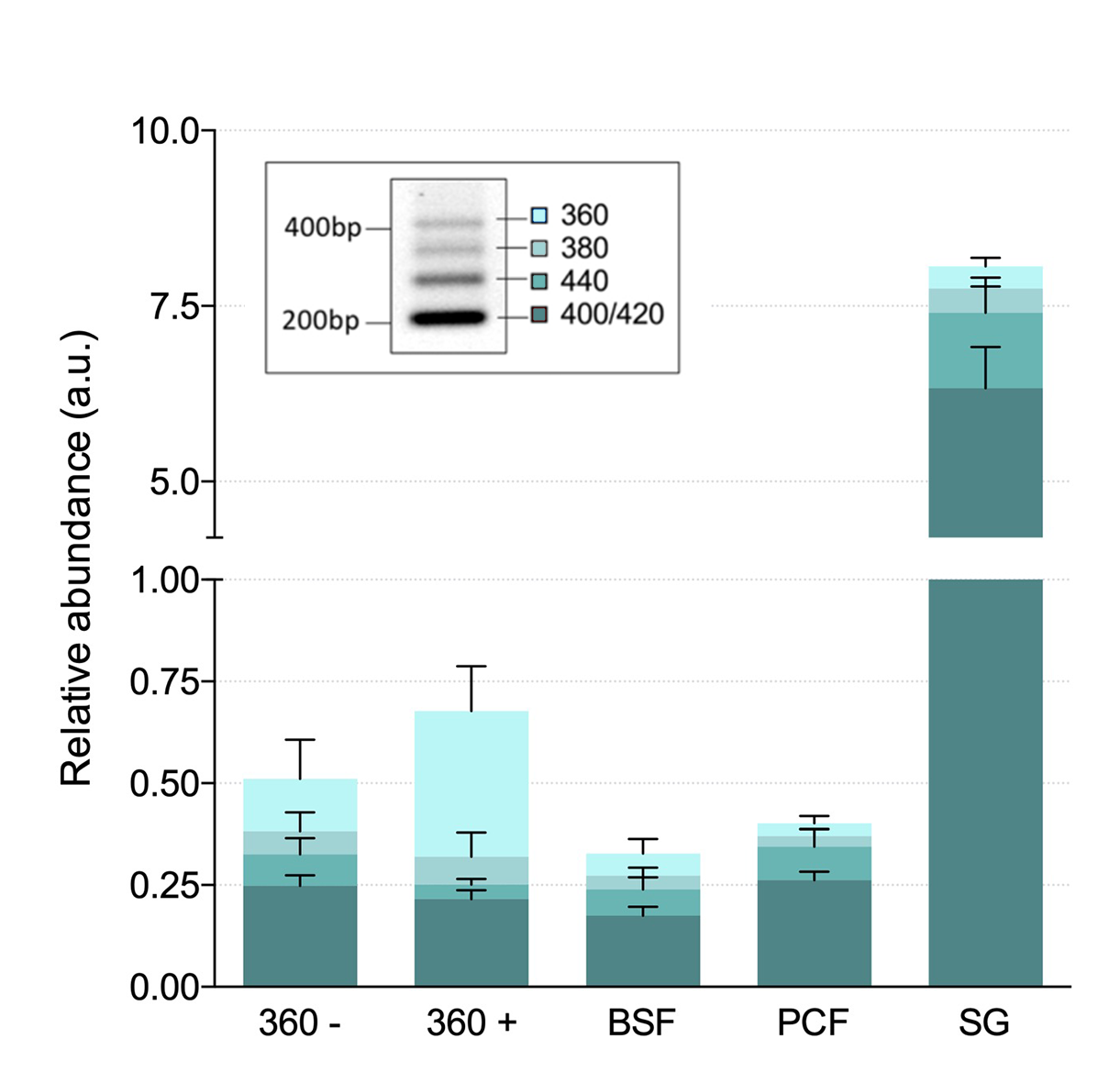

Supplement: S4 Fig — BSF, PCF and SG samples shown as in Fig 2A. The HA-GFPMISP360 PCF line either uninduced (360 -) or tet induced (360 +) was introduced expression control. The inset is a representative image of a DNA agarose gel displaying 4 bands corresponding to the RT-PCR amplification of the different misp homologs. Expression of the different misp isoforms was normalized to the expression of tert. Error bars represent +S.D. from two technical replicates (n = 2). (TIF) [file ppat.1011269.s004.tif]

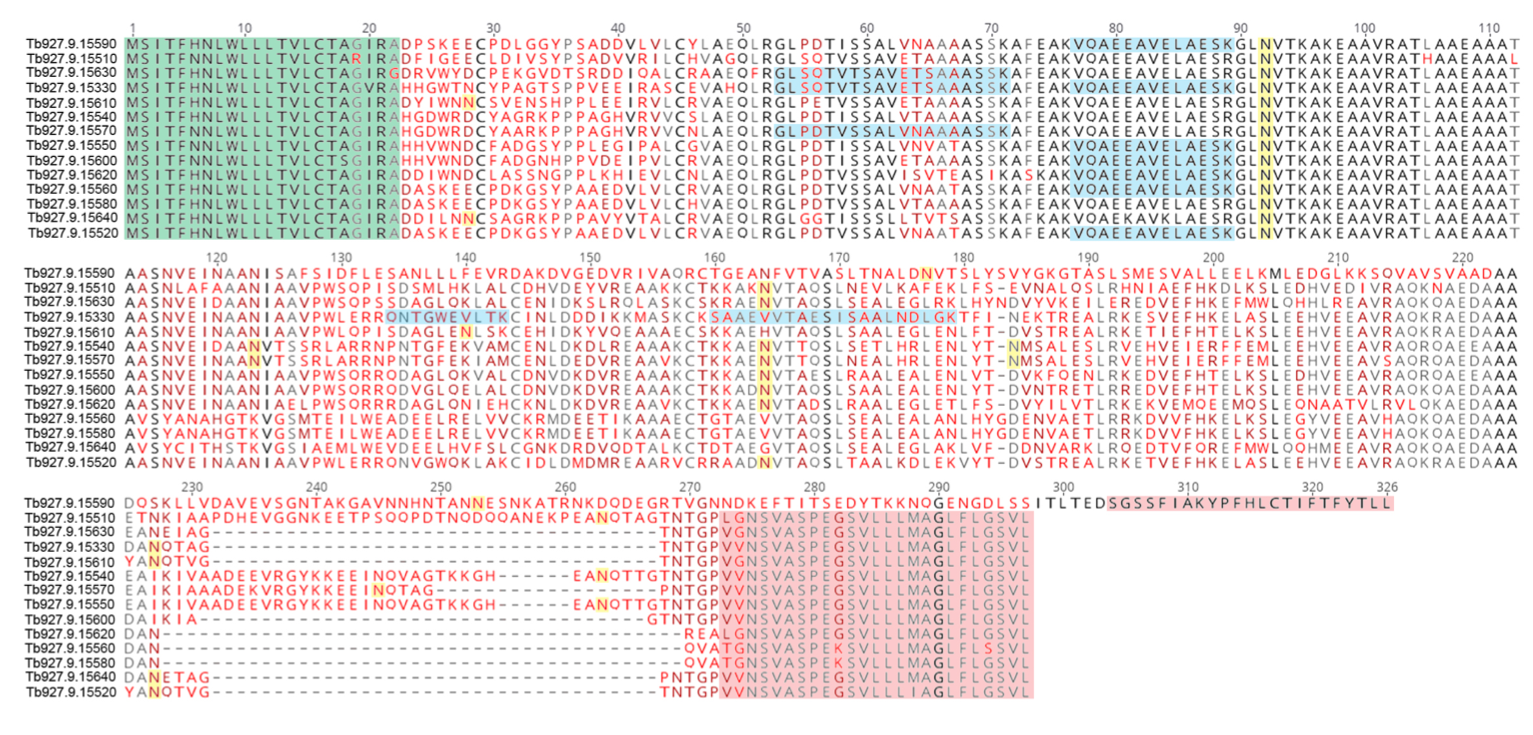

Supplement: S5 Fig — Residues colored based on identity conservation from black (maximum) to red (minimum). Alignment made using a Clustal Omega BLOSUM62 matrix with a gap open cost 10 and a gap extension cost of 0.1, using Geneious R9. The detected BARP peptides in the nLC-MS/MS analysis are highlighted in blue. Domains highlighted: ER signal peptides (green), potential N-glycosylation sites (yellow asparagine residues) and GPI-anchor attachment signal peptides (red). (TIF) [file ppat.1011269.s005.tif]

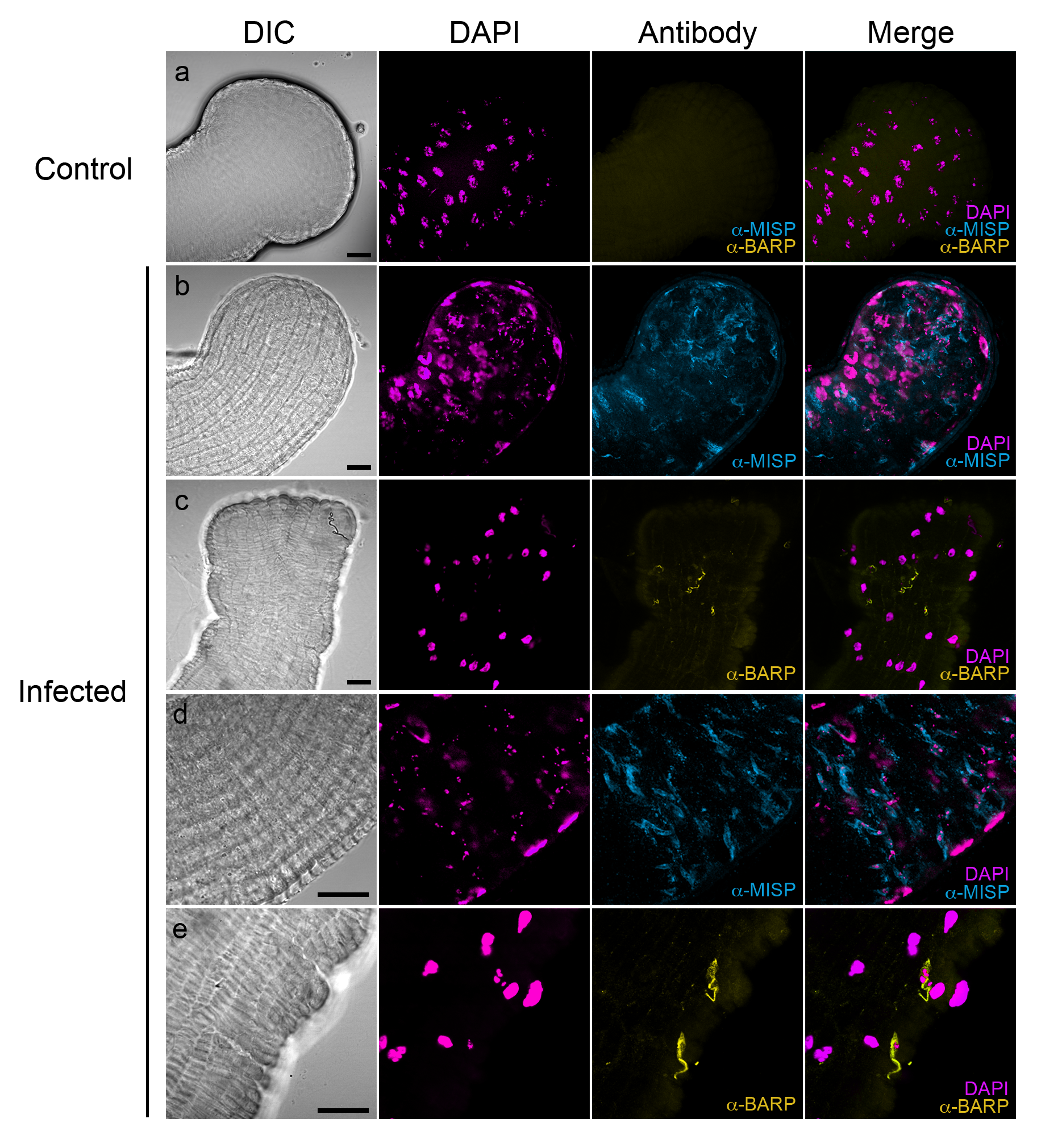

Supplement: S6 Fig — Non-infected salivary gland (Control); a) Tbb-infected (b-e) salivary glands immunostained with either anti-MISP (cyan) or anti-BARP (yellow) polyclonal antibody; DAPI DNA counterstain (magenta); scale bars 200 μm (a, b, c) and 20 μm (d, e). (TIF) [file ppat.1011269.s006.tif]

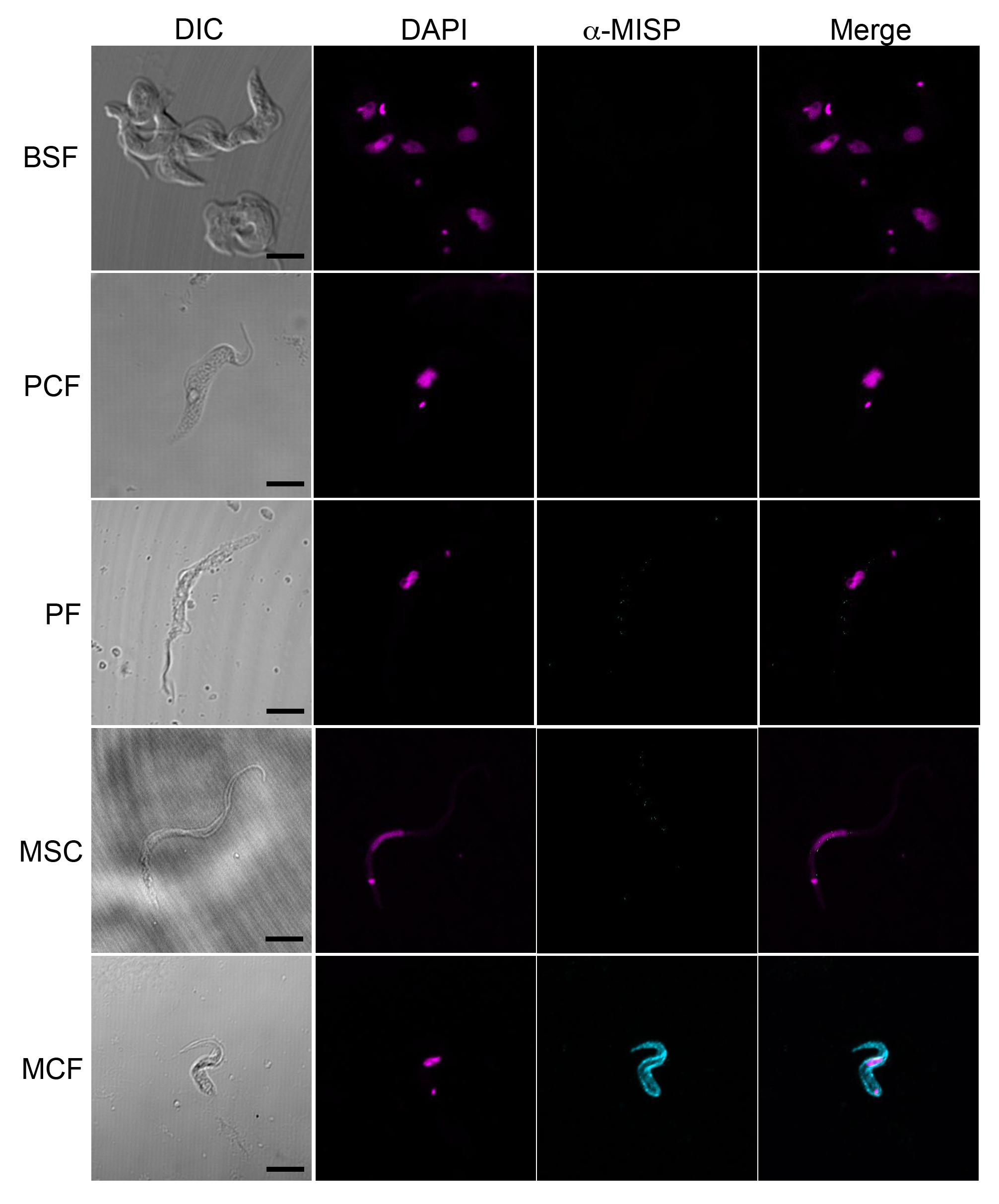

Supplement: S7 Fig — T. b. brucei AnTat 1.1 cultured bloodstream forms (BSF), procyclic cultured form (PCF), midgut procyclic form (PF), proventricular mesocyclic form (MSC), and metacyclic form (MCF) immunostained with anti-MISP polyclonal antibody (cyan); DAPI (magenta); merged and differential interference contrast (DIC); scale bars = 5 mm. (TIF) [file ppat.1011269.s007.tif]

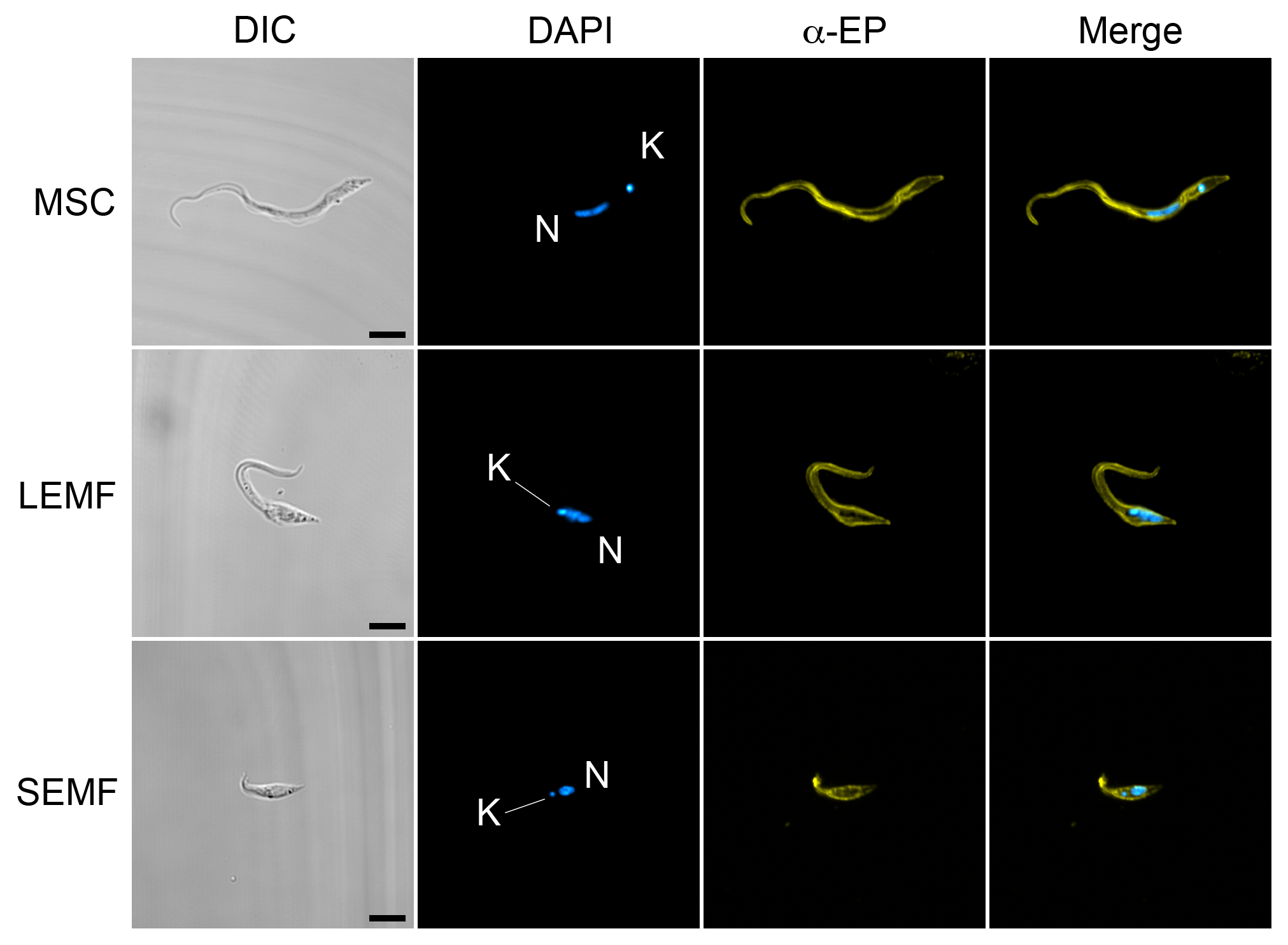

Supplement: S8 Fig — Parasites extracted from infected PV at 30 d.p.i. Mesocyclics (MSC), long epimastigote forms (LEMF) and short epimastigote forms (SEMF) probed with anti-EP monoclonal antibody (yellow) and DAPI (blue). Nuclei (N) and kinetoplastids (K) noted in the blue channel. Scale bars: 5 μm. (TIF) [file ppat.1011269.s008.tif]

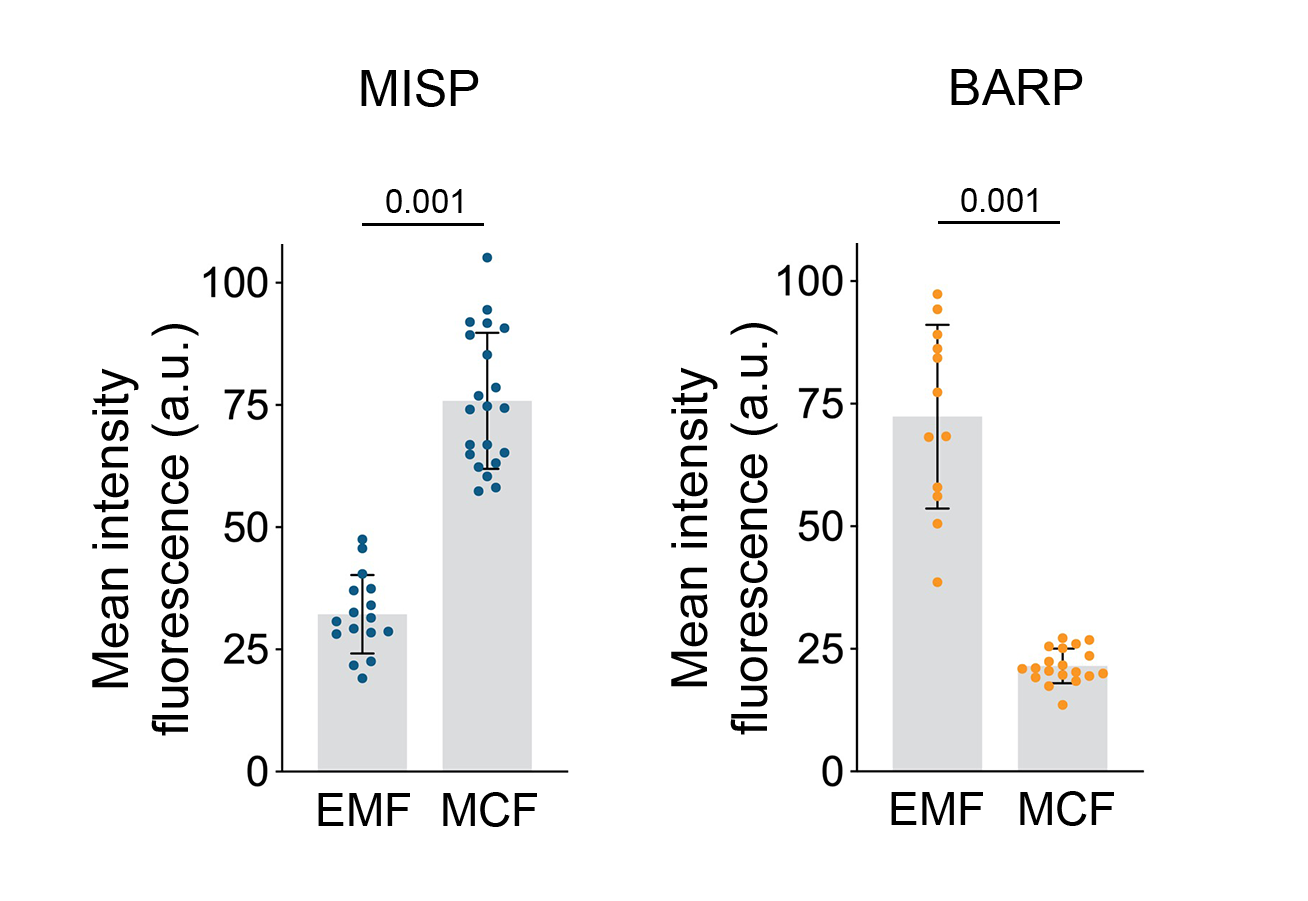

Supplement: S9 Fig — Anti-MISP (blue) and anti-BARP (orange) staining in attached epimastigote forms (EMF) and metacyclic forms (MCF); bars represent mean values; dots represent individual cell values. error bars indicate ±S.D. (TIF) [file ppat.1011269.s009.tif]

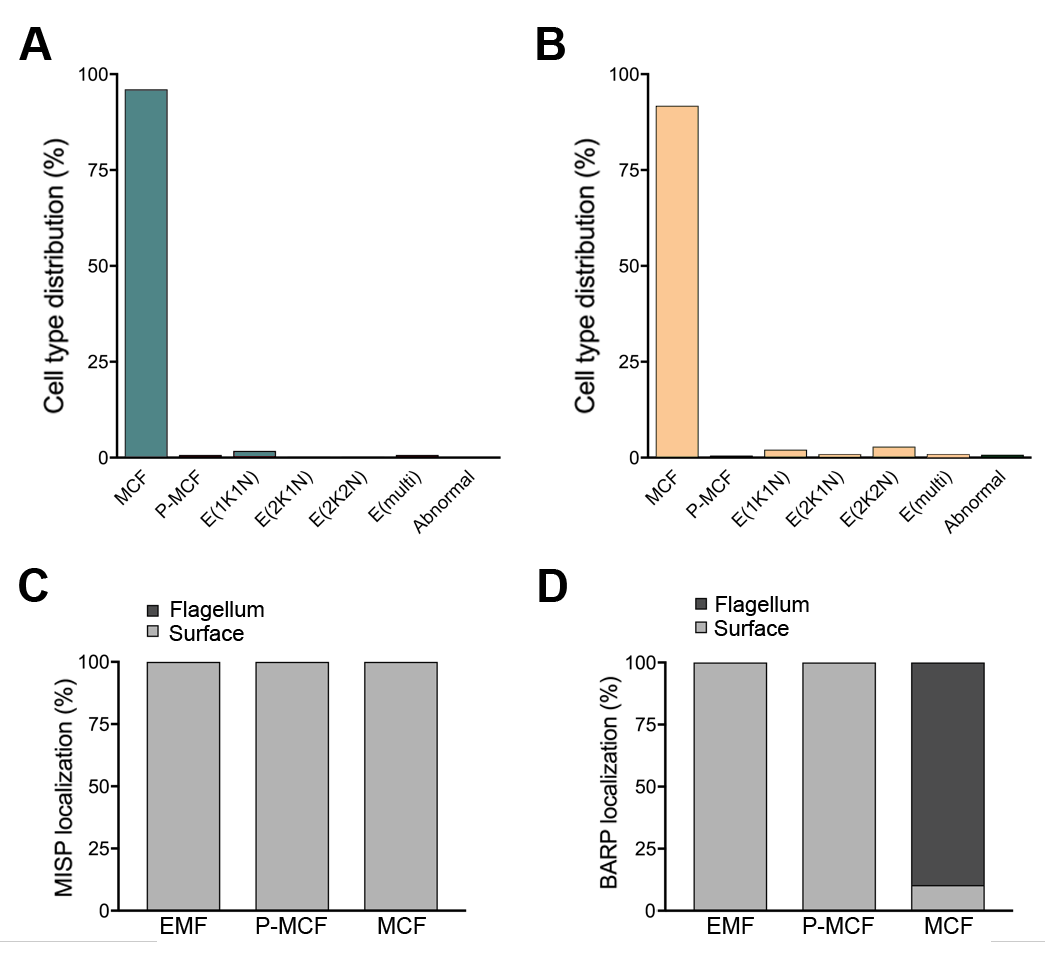

Supplement: S10 Fig — Cell type distribution (percentage) in SG-extracted parasites used for the quantification of mean fluorescence intensities of MISP (A) and BARP (B). Metacyclic forms (MCF), pre-metacyclic forms (P-MCF), epimastigotes with variable kDNA (K) and nuclei (N) numbers (E(1K1N), E(2K1N), E(2K2N)), multi-nucleated epimastigotes (E(multi)) and abnormal cells. C, Distribution (percentage) of types of localization of MISP and BARP (D), either flagellar (flagellum) or on the whole cell surface (surface). (TIF) [file ppat.1011269.s010.tif]

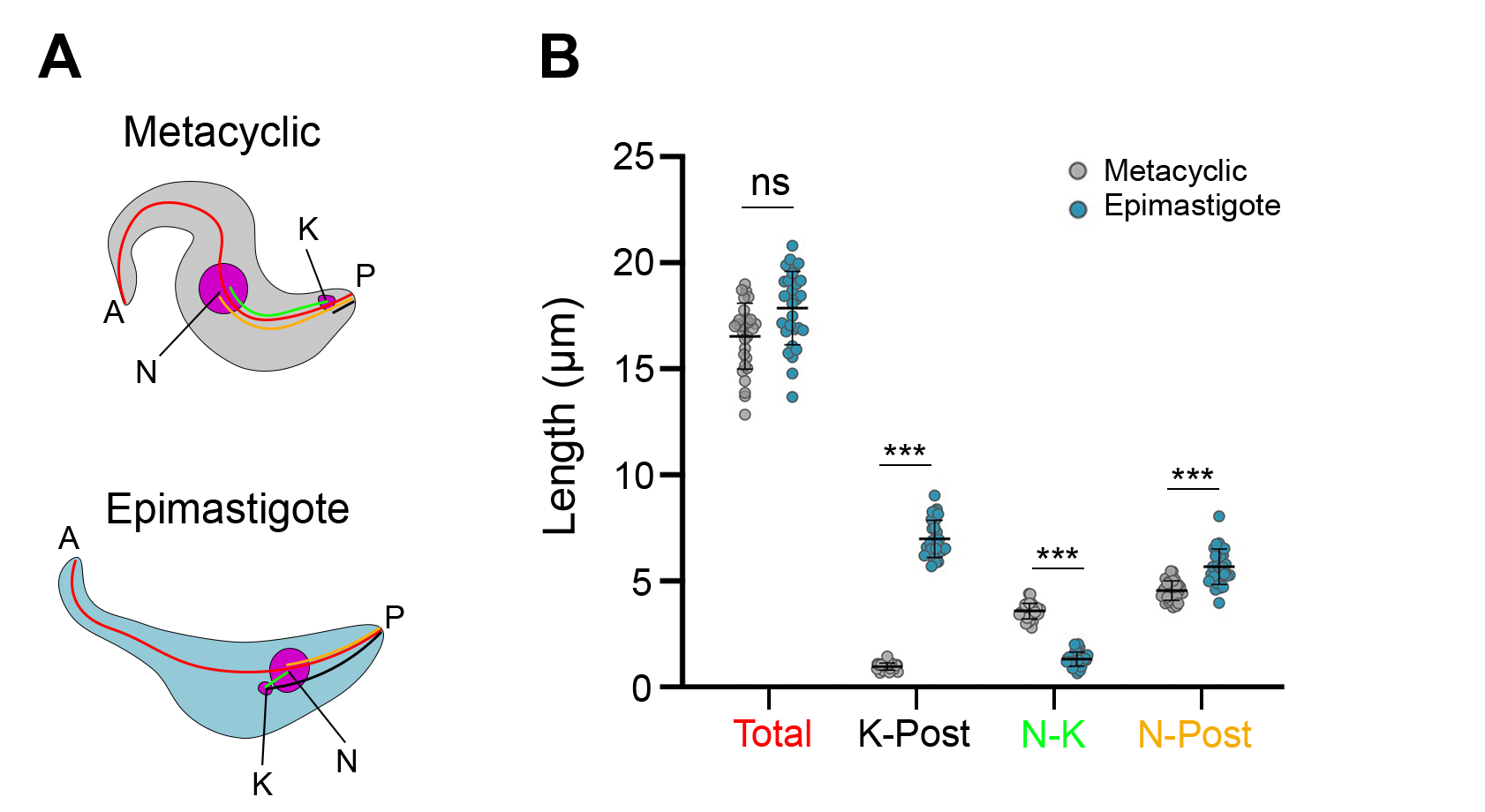

Supplement: S11 Fig — A, Schematic of a metacyclic and an epimastigote cell indicating the measurements in ‘B’: total cell length (red), nucleus to posterior end (orange), nucleus to kDNA (green), and kDNA to posterior end (black); anterior end (A), posterior end (P), kDNA (K), nucleus (N). B, Length measurements (mm) described in ‘A’ in metacyclic (n = 30; gray) and epimastigote cells (n = 30; blue) isolated from tsetse infected salivary glands (from 3 different biological replicates); circles indicate individual data points; thick horizontal line indicates mean value; error bars indicate ±SD; asterisks indicate significance (*** p<0.001); ns indicates non-significant differences; from one-sided t-test assuming normal distribution. (TIF) [file ppat.1011269.s011.tif]

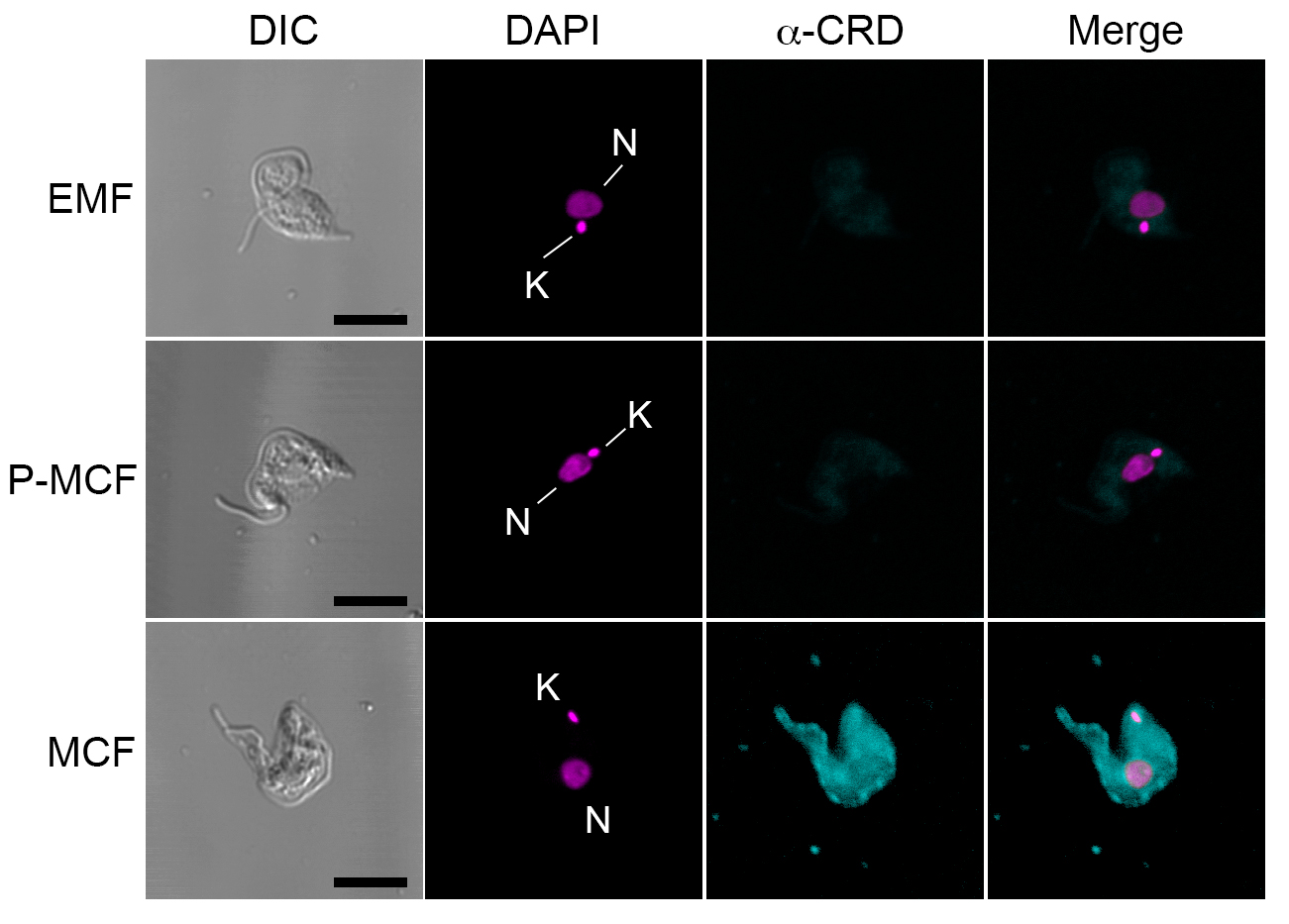

Supplement: S12 Fig — Immunostaining of epimastigotes (EMF), pre-metacyclics (P-MCF) and metacyclics (MCF) from tsetse infected SG with polyclonal anti-CRD antibody. Nuclei (N) and kinetoplastids (K) noted in the magenta channel. Differential interference contrast (DIC), DAPI (magenta) and anti-CRD (blue). Scale bars: 5 μm. (TIF) [file ppat.1011269.s012.tif]

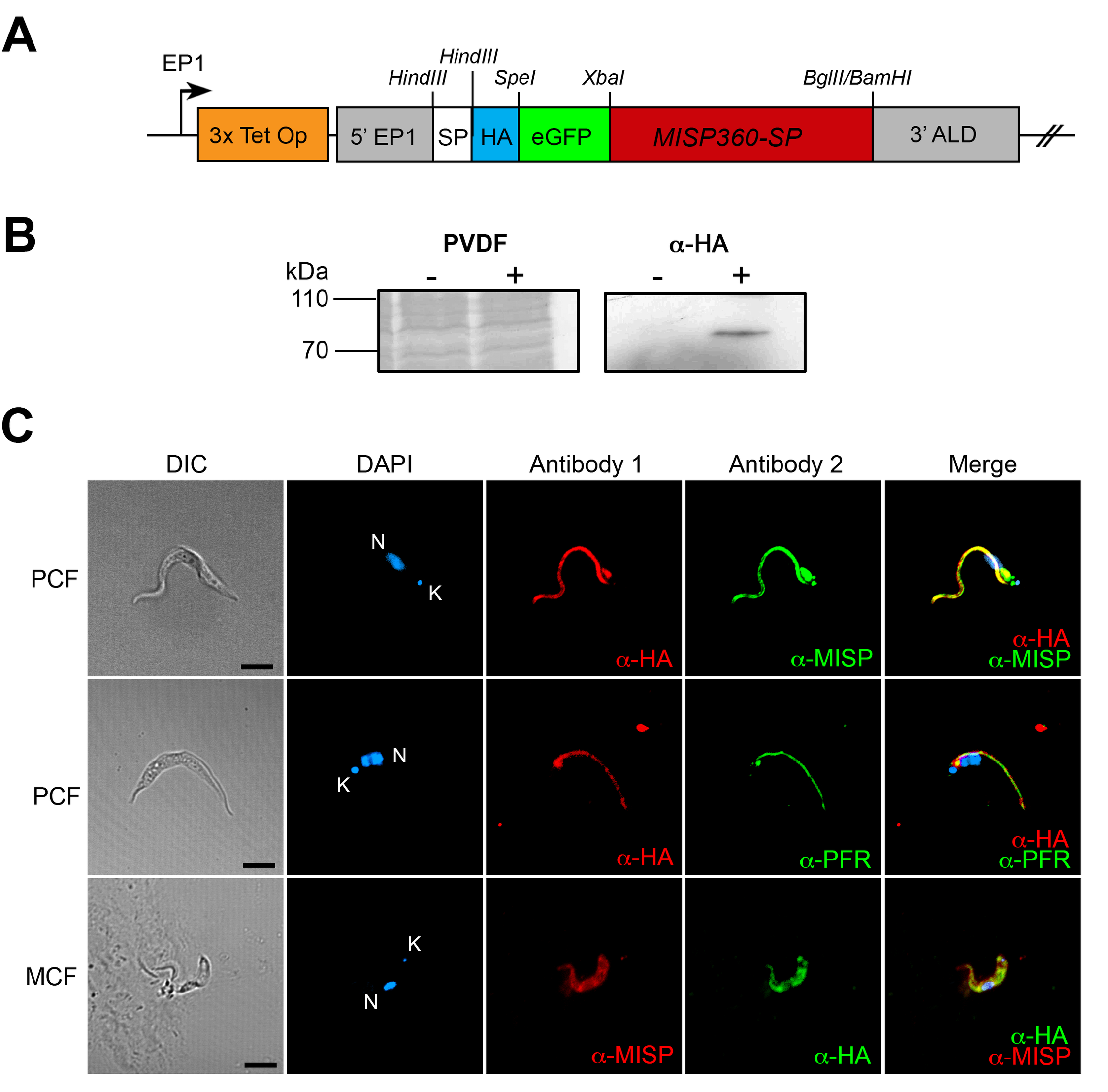

Supplement: S13 Fig — A, DNA construct encoding for the ectopic tagged MISP360. B, Immunoblotting to detect ectopic (HA-tagged) MISP360 expressed by the mutant HA-GFPMISP360 PCF cell line, either with the transgene uninduced (-) or tet-induced (+), probed with anti-HA (top). PVDF membrane stained with nigrosine after film exposure for sample loading control. C, Cellular localization of the HA-tagged ectopic MISP360 in AnTat 1.1 90:13 procyclic (PCF) and metacyclic form (MCF), detected by immunostaining on PFA-fixed non-permeabilized PCF cells with either anti-HA plus anti-MISP (co-localization of ectopic tag with MISP), or with anti-HA plus anti-PFR (flagellar marker) in methanol-fixed cells. Scale bars: 5 μm. (TIF) [file ppat.1011269.s013.tif]

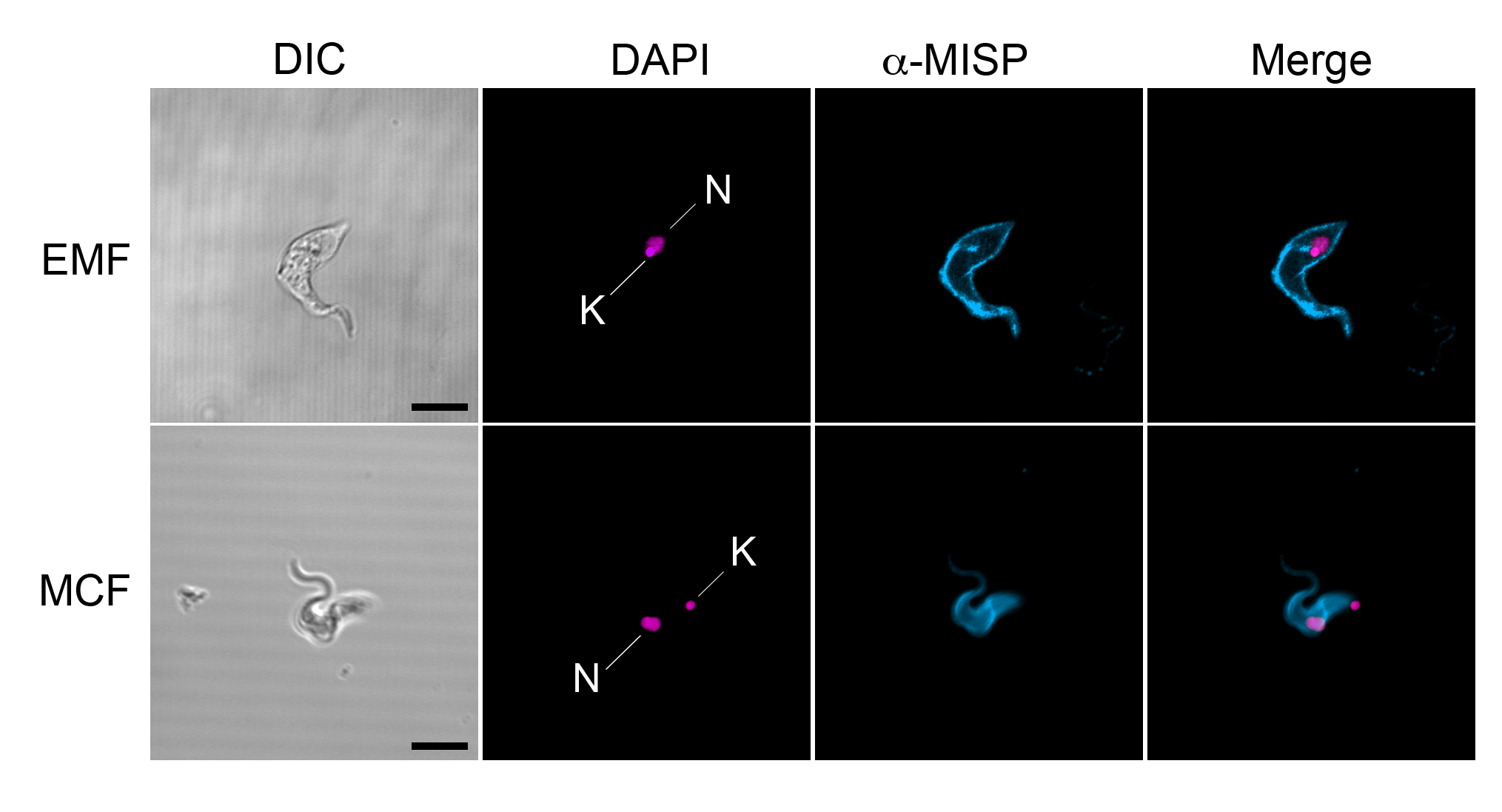

Supplement: S14 Fig — Representative images of live immunostaining of SG parasites using anti-MISP. Epimastigote (EMF) and metacyclic form (MCF) stained with anti-MISP (cyan) and DAPI (magenta). Nuclei (N) and kinetoplasts (K) noted in the blue channel. Scale bars: 5μm. DIC: differential interference contrast. (TIF) [file ppat.1011269.s014.tif]

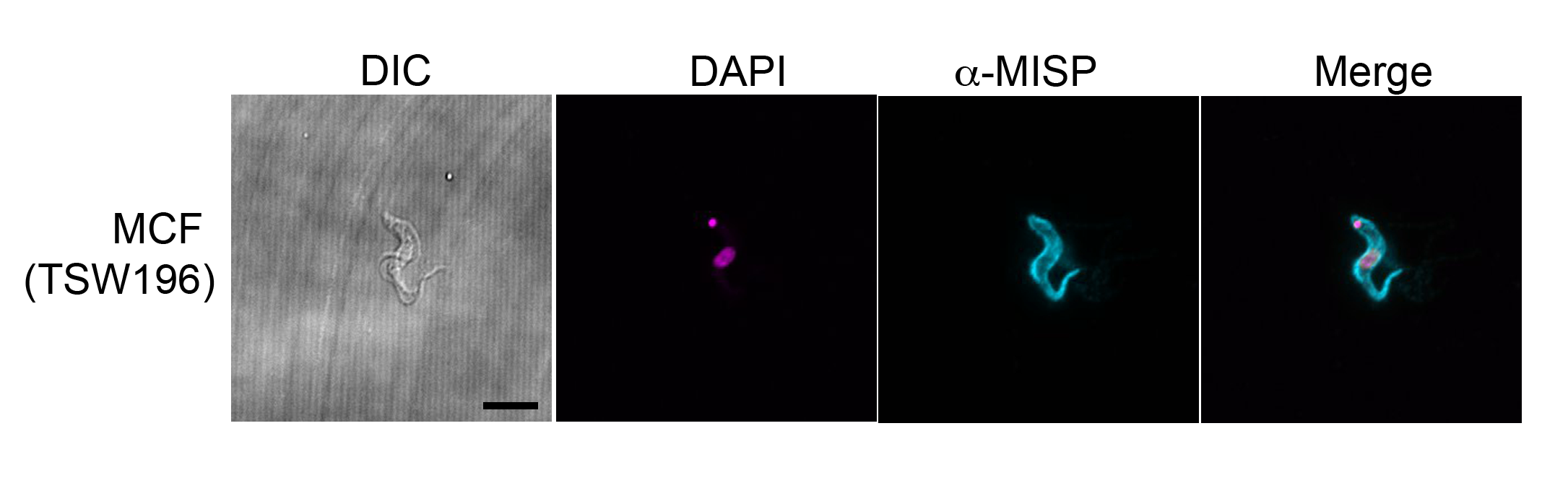

Supplement: S15 Fig — Representative metacyclic cell (MCF) obtained from infected tsetse salivary glands immunostained with polyclonal anti-MISP antibody (cyan); DAPI (magenta); merge, and differential interference contrast (DIC); scale bar = 5 μm. TSW-196 epimastigote and pre-metacyclic cells were also detected by the anti-MISP antibody. Salivary gland stages obtained from three independent tsetse infections with TSW-196 strain were performed and successfully immunostained with anti-MISP polyclonal antibody. (TIF) [file ppat.1011269.s015.tif]

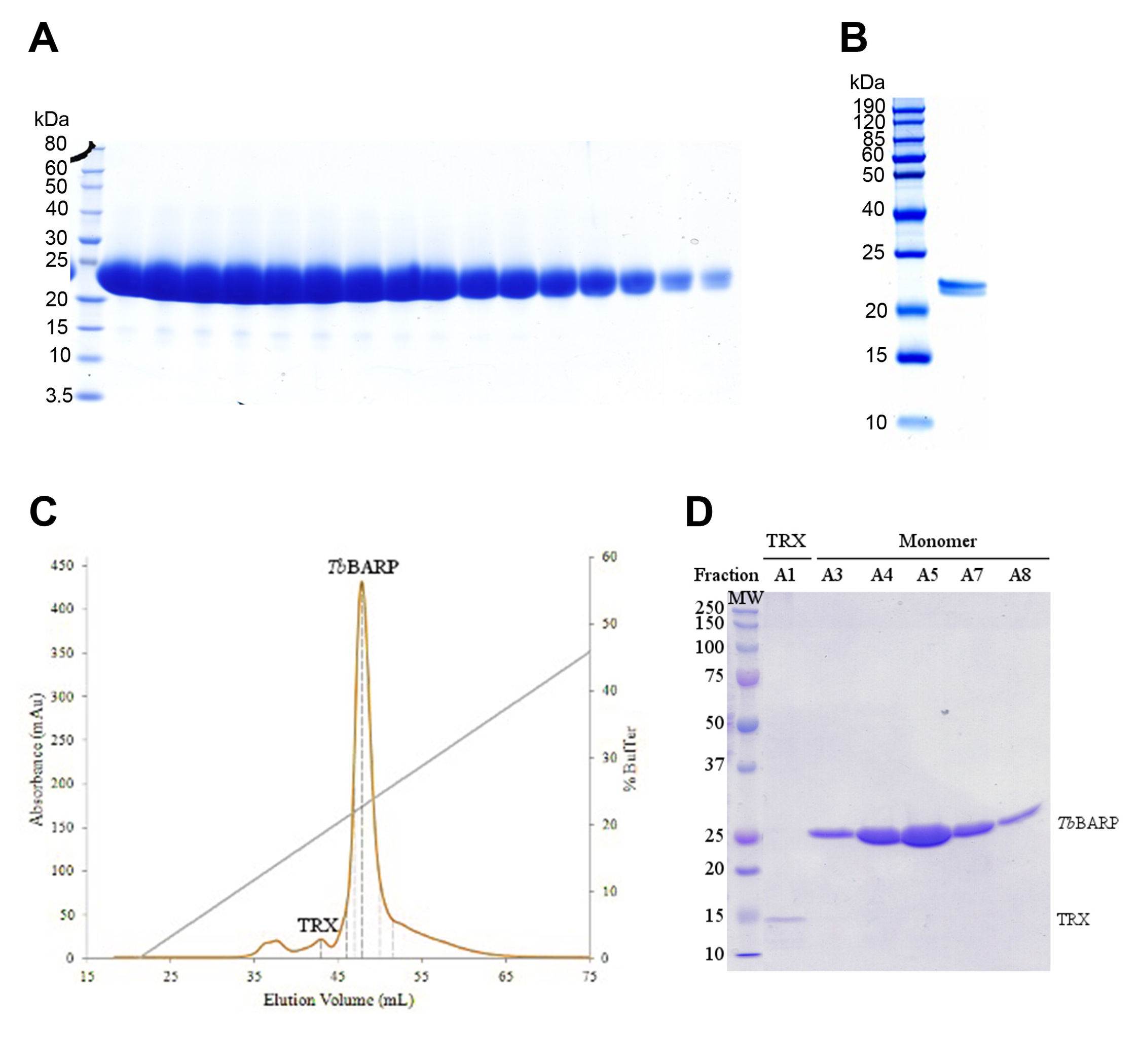

Supplement: S16 Fig — A, SDS-PAGE (Coomassie blue-stained) of SEC elution fractions of the recombinant MISP360 ectodomain (ladder displaying relative molecular weight on the left (kDa). B, Complete SDS-PAGE with fraction of rMISP360 used to determine its crystal structure. C, Size exclusion chromatogram of rBARP used for crystallography (peaks for free TRX tag and rBARP monomer indicated). D, SDS-PAGE (Coomassie-stained) of rBARP fractions. (TIF) [file ppat.1011269.s016.tif]

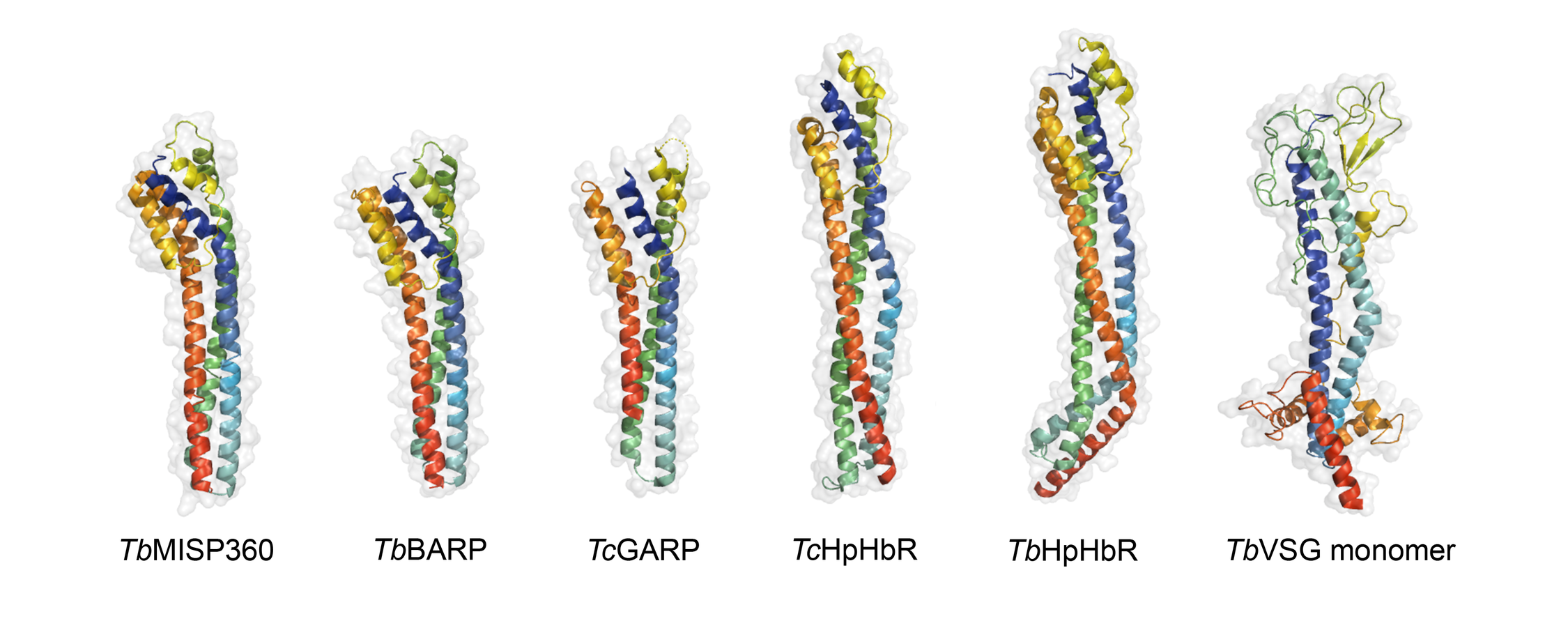

Supplement: S17 Fig — MISP360 (PDB: 5VTL), BARP (high confidence model), GARP (PDB: Y44), TcHpHbR (PDB: 4E40), TbHpHbR (PDB: 4X0J), VSG 221 monomer (PDB: 1VSG). Structures colored from blue (N-terminus) to red (C-terminus); molecular surface in semi-transparent grey. (TIF) [file ppat.1011269.s017.tif]

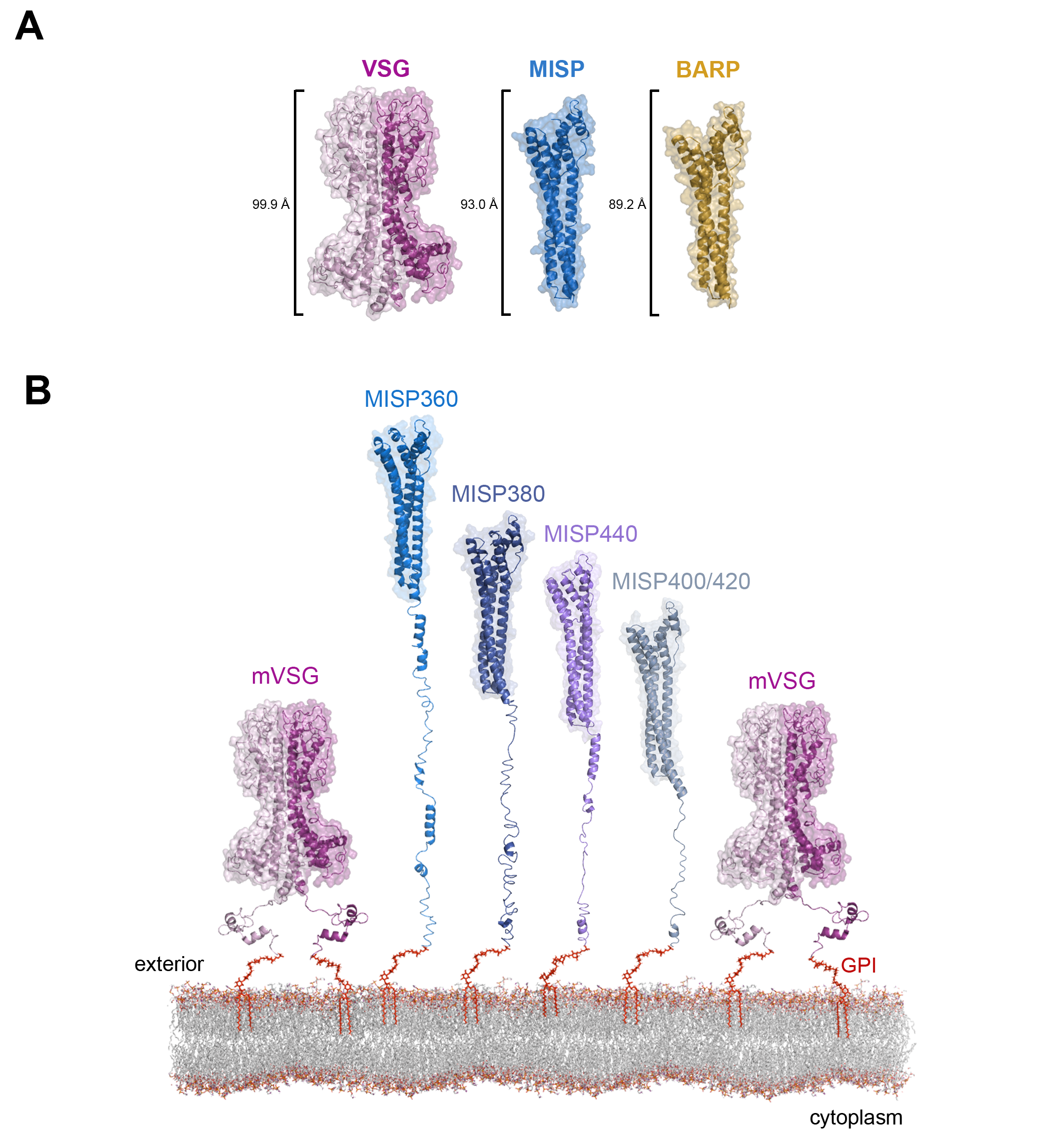

Supplement: S18 Fig — A, Height comparison between the crystal structure of VSG MiTat 1.2 N-terminus (PDB: 1VSG), the MISP360 N-terminus and the BARP model. B, Models of the five T. brucei MISP isoforms on the metacyclic surface. mVSGs modelled using the mVAT4 protein sequence and the structures of VSG MiTat 1.2 N- (PDB: 1VSG) and C-terminus (PDB: 1XU6). MISPs modelled using the crystal structure of MISP360 N-terminus. (TIF) [file ppat.1011269.s018.tif]

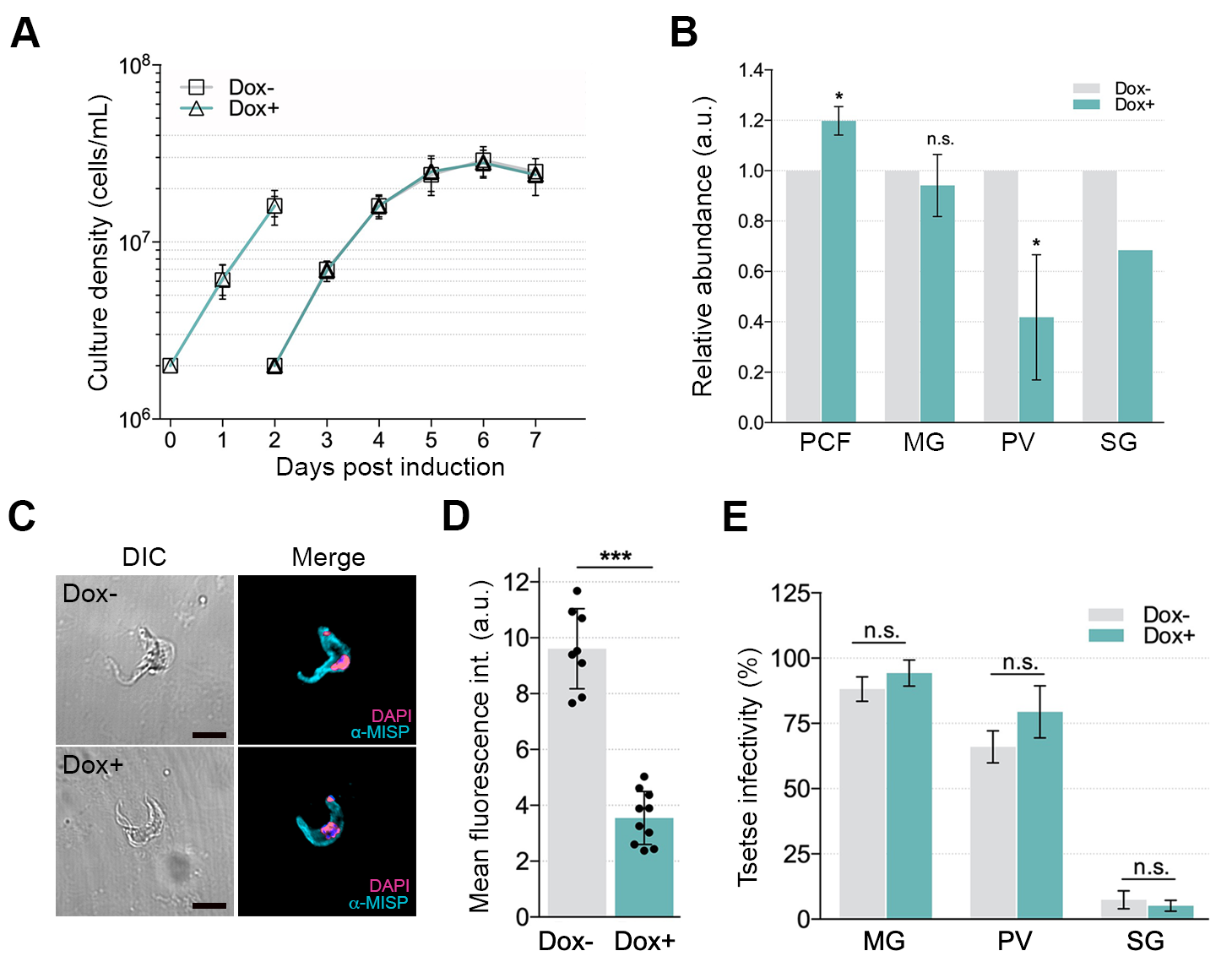

Supplement: S19 Fig — A, In vitro growth curve of mispRNAi PCF cells, either induced (Dox+) or uninduced (Dox-). B, Relative misp RNA expression in mispRNAi PCF cells, midgut procyclics (MG), proventricular parasites (PV) and salivary gland forms (SG). Expression levels of Dox- cells are normalized to 100%. C, Representative immunostaining images of non-permeabilized mispRNAi MCF (Dox-/+) with anti-MISP (red), DAPI (blue) and phase. D, MISP mean fluorescence intensities (arbitrary units) on mispRNAi MCF (Dox-/+). E, Percentage of flies with mispRNAi cells infecting the midgut (MG), proventriculus (PV) and salivary glands (SG). Scale bars: 5μm. Error bars show standard deviation, asterisks represent significance (* for p-value < 0.05; *** for p-value < 0.001). (TIF) [file ppat.1011269.s019.tif]

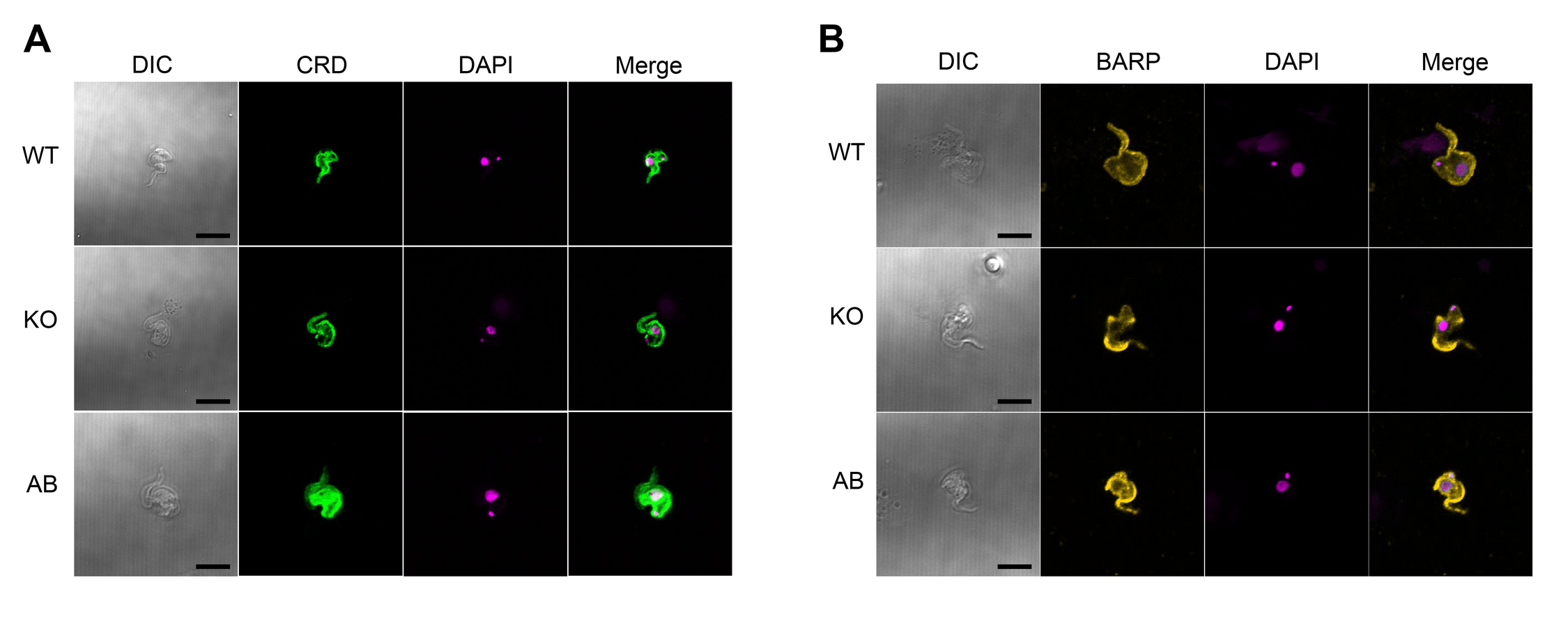

Supplement: S20 Fig — Cells were immunostained with either anti-CRD polyclonal antibody (A, green), or anti-BARP polyclonal antibody (B, yellow); DAPI (magenta), merged, and differential interference contrast (DIC); scale bars = 5 mm. (TIF) [file ppat.1011269.s020.tif]

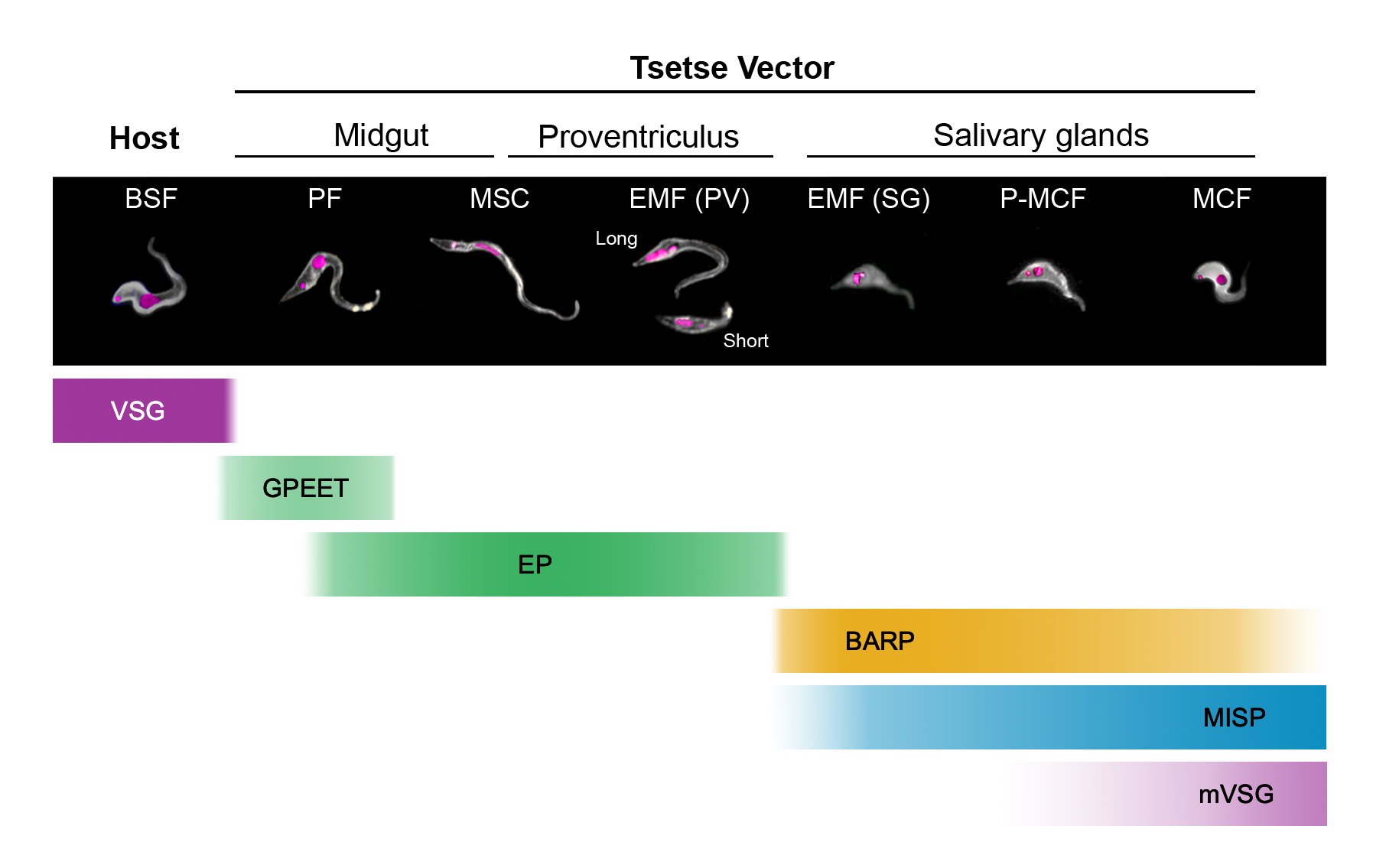

Supplement: S21 Fig — Bloodstream forms (BSF), procyclic forms (PF), mesocyclic forms (MSC), short (SE) and long (LE) epimastigotes infecting the proventriculus, attached epimastigotes infecting the salivary glands (EMF (SG)), pre-metacyclic forms (P-MCF), metacyclic forms (MCF). Representative immunostaining images of the parasite stages highlighting the nucleus and kinetoplast (magenta), and the cell surface (white) (top). Bottom bars define the duration of the expression of the surface proteins in relation to parasite developmental stage. (TIF) [file ppat.1011269.s021.tif]

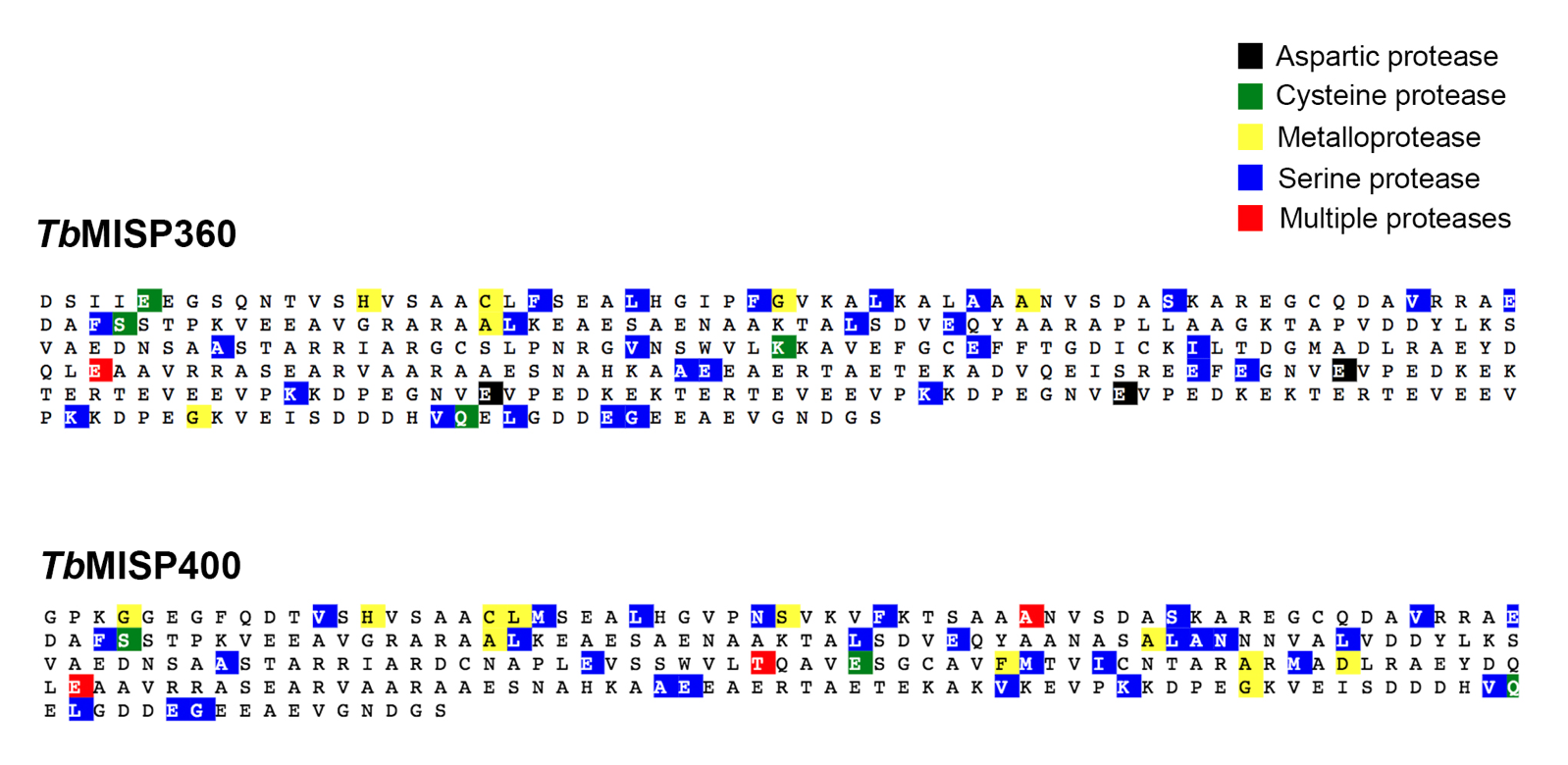

Supplement: S22 Fig — MISP360 (top) and MISP400 (bottom) amino acidic sequences with predicted protease cleavage sites highlighted (see key for protease types). Only one member of each MISP subfamily is represented. (TIF) [file ppat.1011269.s022.tif]

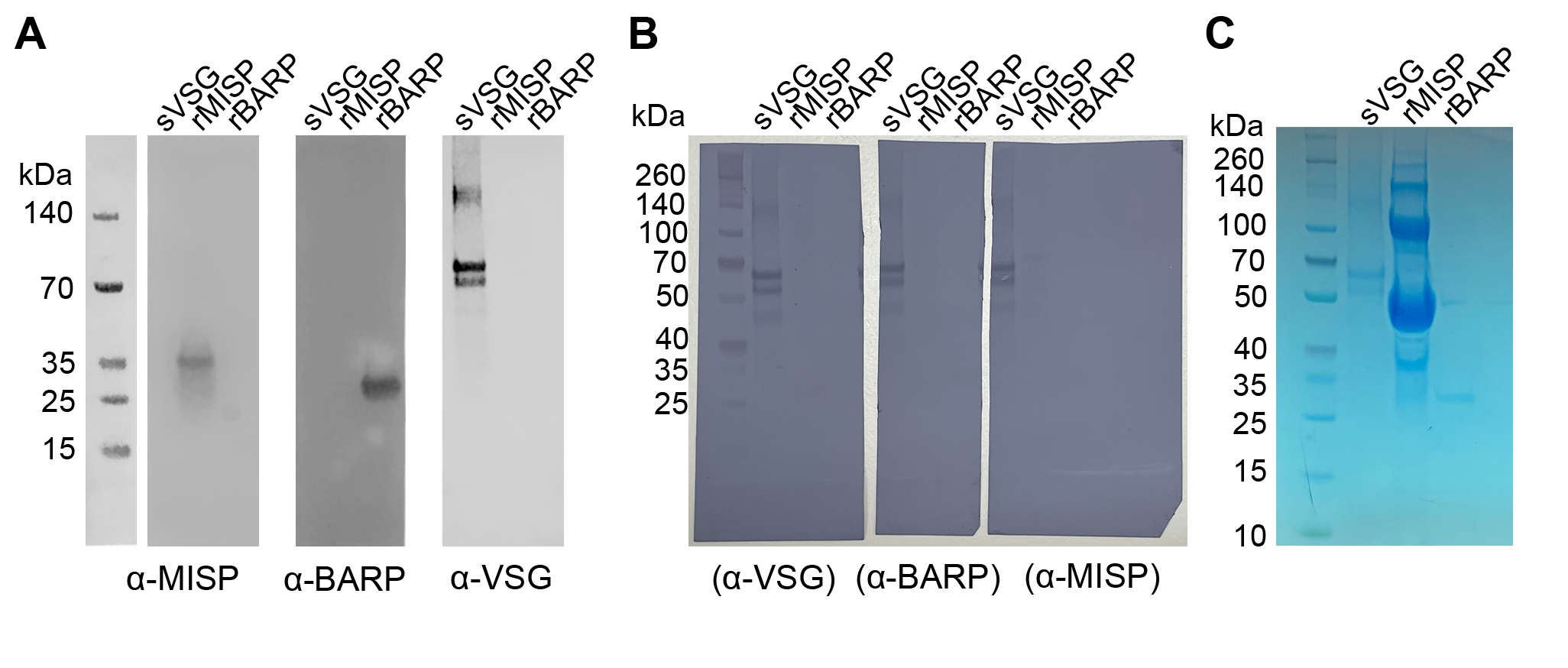

Supplement: S23 Fig — A, Western blotting of soluble VSG 221 (sVSG), full recombinant MISP380 (rMISP), and recombinant BARP (rBARP) probed with either anti-MISP polyclonal antibody (left), or anti-BARP polyclonal antibody (centre), or anti-VSG 221 polyclonal antibody (right); ladder on the left for apparent molecular weights (kDa). B, Blotting membranes developed in ‘A’ stained with nigrosine. C, Instablue-stained SDS-PAGE analysis of same samples as in ‘A’. (TIF) [file ppat.1011269.s023.tif]
