# Supplementary material for: The Trypanosoma brucei MISP family of invariant proteins is co-expressed with BARP as triple helical bundle structures on the surface of salivary gland forms, but is dispensable for parasite development within the tsetse vector
Source: PLoS Pathog. 2023 Mar 30;19(3):e1011269. doi: 10.1371/journal.ppat.1011269 (PMC10089363; doi:10.1371/journal.ppat.1011269)
Supplement: S1 File — (PDF) [file ppat.1011269.s024.pdf]

A. BARP peptides (1)

Sequence C...	Protein	Accession	Category	Bio Sample	MS/M...	Prob	%Spec	#Pep	#Unique	#Spec	%Cov	m.w.
	BARP protein OS=Trypanosoma brucei brucei (strain 927/4 GUTat10.1) GN=Tb09.244.2510 PE=4 SV=1	Q38CW0_TRYB2	Infected	D1_Infected		100%	0.0065%	1	1	2	8.8%	27 kDa

Valid	Weight	Sequence	SEQU...	Prob	SEQU...	NTT	Modifications	Observed	Actual Mass	Charge	Delta ...	Delta ...	Reten...	Intens...	TIC	Start	Stop	#...	...	Spectrum ID
☑	0.8	(K)VQAEEAVELAESK(G)	4.51	100%	0.63	2		701.86	1,401.70	2	0.0020	1.4	3530		45010	77	89	0		Dataset1 Infected-11787-14096_14096
☑	1.0	(R)QNTGWEVLTK(C)	2.19	99%	0.42	2		588.31	1,174.60	2	0.0017	1.4	3660		37860	134	143	0		Dataset1 Infected-12352-14699_14699

Q38CW0_TRYB2 (100%), 27,491.5 Da
BARP protein OS=Trypanosoma brucei brucei (strain 927/4 GUTat10.1) GN=Tb09.244.2510 PE=4 SV=1
1 exclusive unique peptides, 1 exclusive unique spectra, 2 total spectra, 23/260 amino acids (9% coverage)

M S I T F H N L W L   L L T V L C T A G V   R A H H G W T N C Y   P A G N S P P V E E   I R A S C E V A H Q   L R G L S Q T V T S   A V E T S A A A S S   K A F E A K **V Q A E**
E A V E L A E S K G   L N V T K A K E A A   V R A T L A A E A A   A T A A S N V E I N   A A N I A A V P W L   E R R **Q N T G W E V**   **L T K** C I N L D D D   I K K M A S K C K S
A A E V V T A E S I   S A A L N D L G K T   F I N E K T R E A L   R K E S V E F H K E   L A S L E E H V E E   A V R A Q K R A E D   A A A D A N Q T A G   T N T G P V V N S V
A S P E G S V L L L   M A G L F L G S V L

Peptide: VQAEEAVELAESK; Spectrum 1

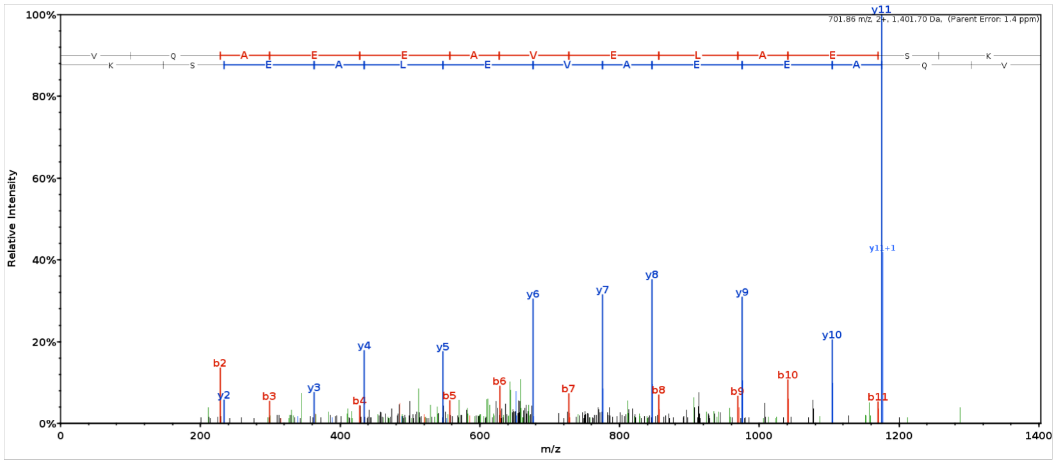

B	B Ions	B+2H	B-NH3	B-H2O	AA	Y Ions	Y+2H	Y-NH3	Y-H2O	Y
1	100.1				V	1,40...	701.9	1,38...	1,38...	13
2	228.1		211.1		Q	1,30...	652.3	1,28...	1,28...	12
3	299.2		282.1		A	1,17...	588.3	1,15...	1,15...	11
4	428.2		411.2	410.2	E	1,10...	552.8	1,08...	1,08...	10
5	557.3		540.2	539.2	E	975.5	488.3	958.5	957.5	9
6	628.3	314.7	611.3	610.3	A	846.5	423.7	829.4	828.4	8
7	727.4	364.2	710.3	709.4	V	775.4	388.2	758.4	757.4	7
8	856.4	428.7	839.4	838.4	E	676.4	338.7	659.3	658.3	6
9	969.5	485.2	952.5	951.5	L	547.3		530.3	529.3	5
10	1,040.5	520.8	1,023.5	1,02...	A	434.2		417.2	416.2	4
11	1,169.6	585.3	1,152.5	1,15...	E	363.2		346.2	345.2	3
12	1,256.6	628.8	1,239.6	1,23...	S	234.1		217.1	216.1	2
13	1,402.7	701.9	1,385.7	1,38...	K	147.1		130.1		1

Peptide: QNTGWEVLTK; Spectrum 1

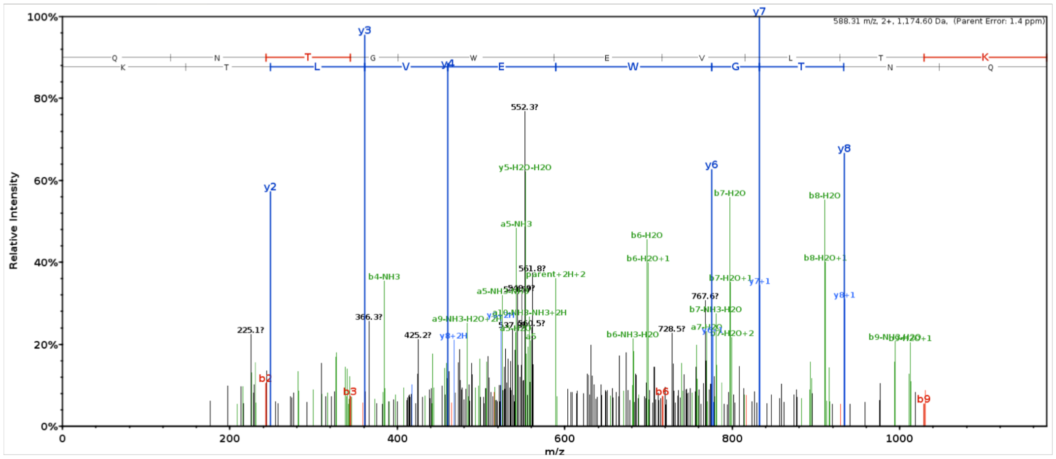

B	B Ions	B+2H	B-NH3	B-H2O	AA	Y Ions	Y+2H	Y-NH3	Y-H2O	Y
1	129.1		112.0		Q	1,17...	588.3	1,15...	1,15...	10
2	243.1		226.1		N	1,04...	524.3	1,03...	1,02...	9
3	344.2		327.1	326.1	T	933.5	467.3	916.5	915.5	8
4	401.2		384.2	383.2	G	832.5	416.7	815.4	814.4	7
5	587.3		570.2	569.2	W	775.4	388.2	758.4	757.4	6
6	716.3	358.7	699.3	698.3	E	589.4		572.3	571.3	5
7	815.4	408.2	798.3	797.4	V	460.3		443.3	442.3	4
8	928.5	464.7	911.4	910.4	L	361.2		344.2	343.2	3
9	1,029.5	515.3	1,012.5	1,01...	T	248.2		231.1	230.1	2
10	1,175.6	588.3	1,158.6	1,15...	K	147.1		130.1		1

B. BARP peptides (2)

Sequence C...	Protein	Accession	Category	Bio Sample	MS/M...	Prob	%Spec	#Pep	#Unique	#Spec	%Cov	m.w.
	BARP protein OS=Trypanosoma brucei brucei (strain 927/4 GUTat10.1) GN=Tb09.244.2510 PE=4 SV=1	Q38CW0_TRYB2	Infected	D3_Infected		100%	0.014%	2	2	5	20%	27 kDa

Valid	Weight	Sequence	SEQU...	Prob	SEQU...	NTT	Modifications	Observed	Actual Mass	Charge	Delta ...	Delta ...	Reten...	Intens...	TIC	Start	Stop	#...	...	Spectrum ID
☑	1.0	(R)GLSQTVTSAVETSAAASSK(A)	5.69	100%	0.63	2		897.96	1,793.90	2	0.000...	0.38	4490		43620	53	71	0		Dataset3 Infected saliva-17607-20268...
☑	0.8	(K)VQAEEAVELAESK(G)	4.30	100%	0.57	2		701.86	1,401.70	2	-0.00...	-0.49	3770		60960	77	89	0		Dataset3 Infected saliva-13943-16337...
☑	0.8	(K)VQAEEAVELAESK(G)	3.67	100%	0.45	2		701.86	1,401.70	2	0.000...	0.21	3820		59390	77	89	0		Dataset3 Infected saliva-14197-16612...
☑	1.0	(K)SAAEVVTAESISAALNDLGK(T)	4.79	100%	0.46	2		973.51	1,945.00	2	-0.00...	-0.32	6810		36180	160	179	0		Dataset3 Infected saliva-29186-32972...
☑	1.0	(K)SAAEVVTAESISAALNDLGK(T)	5.43	100%	0.46	2		973.51	1,945.00	2	0.0017	0.87	6820		30900	160	179	0		Dataset3 Infected saliva-29211-32999...

Q38CW0_TRYB2 (100%), 27,491.5 Da
BARP protein OS=Trypanosoma brucei brucei (strain 927/4 GUTat10.1) GN=Tb09.244.2510 PE=4 SV=1
2 exclusive unique peptides, 2 exclusive unique spectra, 5 total spectra, 52/260 amino acids (20% coverage)

```
MSITFHNLWL LLTVLCTAGV RAHHGWTNCY PAGNSPPVEE IRASCEVAHQ LRGLSQTVTS AVETSAAASS KAFEAKVQAE
EAVELAESKG LNVTKAKEAA VRATLAAEAA ATAASNVEIN AANIAAVPWL ERRQNTGWEV LTKCINLDDD IKKMASKCKS
AAEVVTAESI SAALNDLGKT FINEKTREAL RKESVEFHKE LASLEEHVEE AVRAQKRAED AAADANQTAG TNTGPVVNSV
ASPEGSVLLL MAGLFLGSVL
```

Peptide: GLSQTVTSAVETSAAASSK; Spectrum 1

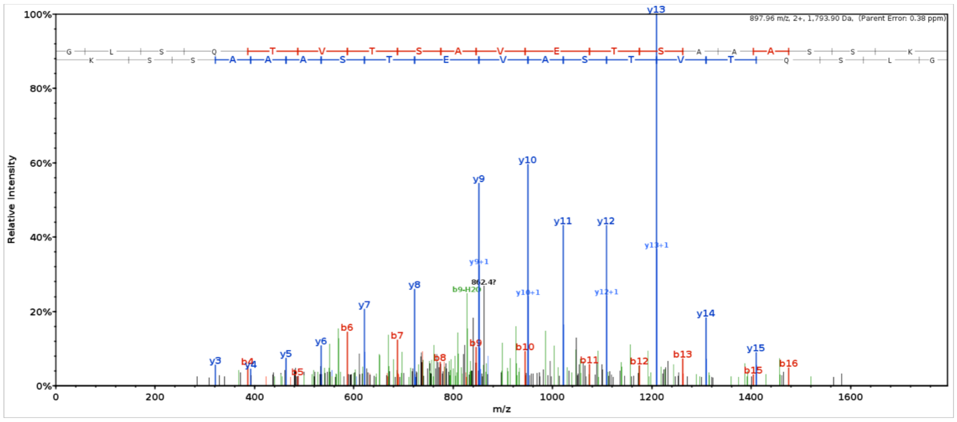

B	B Ions	B+2H	B-NH3	B-H2O	AA	Y Ions	Y+2H	Y-NH3	Y-H2O	Y
1	58.0				G	1,79...	898.0	1,77...	1,77...	19
2	171.1				L	1,73...	869.4	1,72...	1,71...	18
3	258.1			240.1	S	1,62...	812.9	1,60...	1,60...	17
4	386.2		369.2	368.2	Q	1,53...	769.4	1,52...	1,51...	16
5	487.3		470.2	469.2	T	1,40...	705.4	1,39...	1,39...	15
6	586.3	293.7	569.3	568.3	V	1,30...	654.8	1,29...	1,29...	14
7	687.4	344.2	670.3	669.4	T	1,20...	605.3	1,19...	1,19...	13
8	774.4	387.7	757.4	756.4	S	1,10...	554.8	1,09...	1,09...	12
9	845.4	423.2	828.4	827.4	A	1,02...	511.3	1,00...	1,00...	11
10	944.5	472.8	927.5	926.5	V	950.5	475.7	933.5	932.5	10
11	1,073.5	537.3	1,056.5	1,05...	E	851.4	426.2	834.4	833.4	9
12	1,174.6	587.8	1,157.6	1,15...	T	722.4	361.7	705.3	704.4	8
13	1,261.6	631.3	1,244.6	1,24...	S	621.3	311.2	604.3	603.3	7
14	1,332.7	666.8	1,315.6	1,31...	A	534.3	267.6	517.3	516.3	6
15	1,403.7	702.4	1,386.7	1,38...	A	463.3		446.2	445.2	5
16	1,474.7	737.9	1,457.7	1,45...	A	392.2		375.2	374.2	4
17	1,561.8	781.4	1,544.7	1,54...	S	321.2		304.2	303.2	3
18	1,648.8	824.9	1,631.8	1,63...	S	234.1		217.1	216.1	2
19	1,794.9	898.0	1,777.9	1,77...	K	147.1		130.1		1

Peptide: VQAEEAVELAESK; Spectrum 1

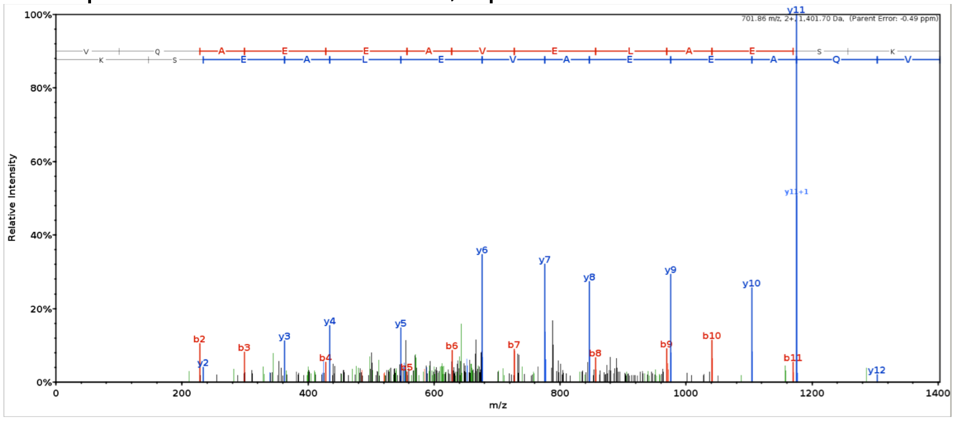

B	B Ions	B+2H	B-NH3	B-H2O	AA	Y Ions	Y+2H	Y-NH3	Y-H2O	Y
1	100.1				V	1,40...	701.9	1,38...	1,38...	13
2	228.1		211.1		Q	1,30...	652.3	1,28...	1,28...	12
3	299.2		282.1		A	1,17...	588.3	1,15...	1,15...	11
4	428.2		411.2	410.2	E	1,10...	552.8	1,08...	1,08...	10
5	557.3		540.2	539.2	E	975.5	488.3	958.5	957.5	9
6	628.3	314.7	611.3	610.3	A	846.5	423.7	829.4	828.4	8
7	727.4	364.2	710.3	709.4	V	775.4	388.2	758.4	757.4	7
8	856.4	428.7	839.4	838.4	E	676.4	338.7	659.3	658.3	6
9	969.5	485.2	952.5	951.5	L	547.3		530.3	529.3	5
10	1,040.5	520.8	1,023.5	1,02...	A	434.2		417.2	416.2	4
11	1,169.6	585.3	1,152.5	1,15...	E	363.2		346.2	345.2	3
12	1,256.6	628.8	1,239.6	1,23...	S	234.1		217.1	216.1	2
13	1,402.7	701.9	1,385.7	1,38...	K	147.1		130.1		1

Peptide: SAAEVVTAESISAALNDLGK; Spectrum 1

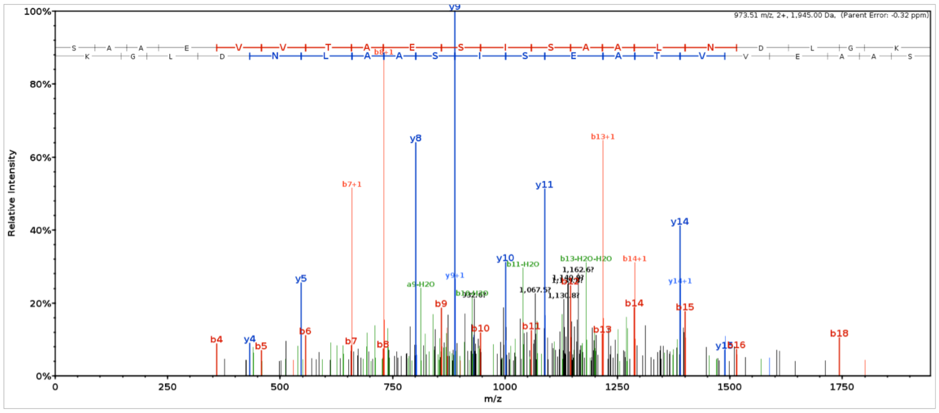

B	B Ions	B+2H	B-NH3	B-H2O	AA	Y Ions	Y+2H	Y-NH3	Y-H2O	Y
1	88.0			70.0	S	1,94...	973.5	1,92...	1,92...	20
2	159.1			141.1	A	1,85...	930.0	1,84...	1,84...	19
3	230.1			212.1	A	1,78...	894.5	1,77...	1,76...	18
4	359.2			341.1	E	1,71...	859.0	1,69...	1,69...	17
5	458.2			440.2	V	1,58...	794.4	1,57...	1,56...	16
6	557.3	279.2		539.3	V	1,48...	744.9	1,47...	1,47...	15
7	658.3	329.7		640.3	T	1,38...	695.4	1,37...	1,37...	14
8	729.4	365.2		711.4	A	1,28...	644.8	1,27...	1,27...	13
9	858.4	429.7		840.4	E	1,21...	609.3	1,20...	1,19...	12
10	945.5	473.2		927.4	S	1,08...	544.8	1,07...	1,07...	11
11	1,058.5	529.8		1,04...	I	1,00...	501.3	984.5	983.6	10
12	1,145.6	573.3		1,12...	S	888.5	444.7	871.5	870.5	9
13	1,216.6	608.8		1,19...	A	801.4	401.2	784.4	783.4	8
14	1,287.6	644.3		1,26...	A	730.4	365.7	713.4	712.4	7
15	1,400.7	700.9		1,38...	L	659.4	330.2	642.3	641.4	6
16	1,514.8	757.9	1,497.7	1,49...	N	546.3		529.3	528.3	5
17	1,629.8	815.4	1,612.8	1,61...	D	432.2		415.2	414.2	4
18	1,742.9	871.9	1,725.9	1,72...	L	317.2		300.2		3
19	1,799.9	900.5	1,782.9	1,78...	G	204.1		187.1		2
20	1,946.0	973.5	1,929.0	1,92...	K	147.1		130.1		1

C. BARP peptides (3)

Sequence C...	Protein	Accession	Category	Bio Sample	MS/M...	Prob	%Spec	#Pep	#Unique	#Spec	%Cov	m.w.
	| transcript=Tbg972.9.9730:mRNA | gene=Tbg972.9.9730 | organism=Trypanoso...	Tbg972.9.9730:mR...	Infected	D3_Infected		92%	0.0055%	0	0	2	7.5%	27 kDa

Valid	Weight	Sequence	SEQU...	Prob	SEQU...	NTT	Modifications	Observed	Actual Mass	Charge	Delta ...	Delta ...	Reten...	Intens...	TIC	Start	Stop	#...	...	Spectrum ID
☑	0.7	(R)GLPDTVSSALVNAAAASSK(A)	4.78	100%	0.69	2		879.96	1,757.92	2	-0.00...	-0.48	5690		148400	53	71	0		Dataset3 Infected saliva-23701-26929...
☑	0.7	(R)GLPDTVSSALVNAAAASSK(A)	4.97	100%	0.65	2		879.97	1,757.92	2	0.000...	0.36	5700		79180	53	71	0		Dataset3 Infected saliva-23754-26989...

Tbg972.9.9730:mRNA-p1 (92%), 26,734.6 Da
| transcript=Tbg972.9.9730:mRNA | gene=Tbg972.9.9730 | organism=Trypanosoma_brucei_gambiense_DAL972 | gene_product=BARP protein | transcript_product=BARP protein | location=Tbg972_09:2091071-2
0 exclusive unique peptides, 0 exclusive unique spectra, 2 total spectra, 19/255 amino acids (7% coverage)

M S I T F H S L W L   L L T V L C T A G I   R A D P N N G E C P   D K G S Y P A A E D   V L V L C H V A E Q   L R **G L P D T V S S**   **A L V N A A A A S S**   **K** A F E A K V Q A E
K A V E L A E S R G   L N V T K A K E A A   V R A T L A A E A A   A T A V S Y A N A H   G T K V G S M T E I   L W E A D E E L R E   L V V C K R M G E E   T I K A A A E C T G
T A E G V T A Q S L   S E A L E A L A N L   H Y G D E N V A E T   L R R K D V V F H K   E L K S L E G Y V E   E A V H A Q K Q A E   D A A A D A N Q V A   V G N S V A S P E G
S V L L L M A G L F   L S S V L

Peptide: GLPDTVSSALVNAAAASSK; Spectrum 1

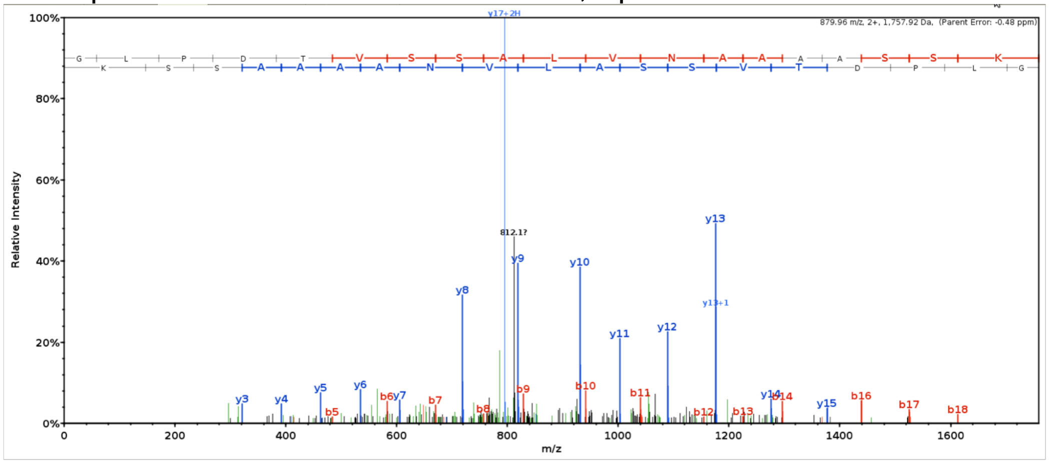

B	B Ions	B+2H	B-NH3	B-H2O	AA	Y Ions	Y+2H	Y-NH3	Y-H2O	Y
1	58.0				G	1,75...	880.0	1,74...	1,74...	19
2	171.1				L	1,70...	851.5	1,68...	1,68...	18
3	268.2				P	1,58...	794.9	1,57...	1,57...	17
4	383.2			365.2	D	1,49...	746.4	1,47...	1,47...	16
5	484.2			466.2	T	1,37...	688.9	1,35...	1,35...	15
6	583.3	292.2		565.3	V	1,27...	638.3	1,25...	1,25...	14
7	670.3	335.7		652.3	S	1,17...	588.8	1,15...	1,15...	13
8	757.4	379.2		739.4	S	1,08...	545.3	1,07...	1,07...	12
9	828.4	414.7		810.4	A	1,00...	501.8	985.5	984.5	11
10	941.5	471.3		923.5	L	931.5	466.3	914.5	913.5	10
11	1,040.6	520.8		1,02...	V	818.4	409.7	801.4	800.4	9
12	1,154.6	577.8	1,137.6	1,13...	N	719.4	360.2	702.3	701.4	8
13	1,225.6	613.3	1,208.6	1,20...	A	605.3	303.2	588.3	587.3	7
14	1,296.7	648.8	1,279.7	1,27...	A	534.3	267.6	517.3	516.3	6
15	1,367.7	684.4	1,350.7	1,34...	A	463.3		446.2	445.2	5
16	1,438.8	719.9	1,421.7	1,42...	A	392.2		375.2	374.2	4
17	1,525.8	763.4	1,508.8	1,50...	S	321.2		304.2	303.2	3
18	1,612.8	806.9	1,595.8	1,59...	S	234.1		217.1	216.1	2
19	1,758.9	880.0	1,741.9	1,74...	K	147.1		130.1		1

Peptide: GLPDTVSSALVNAAAASSK; Spectrum 2

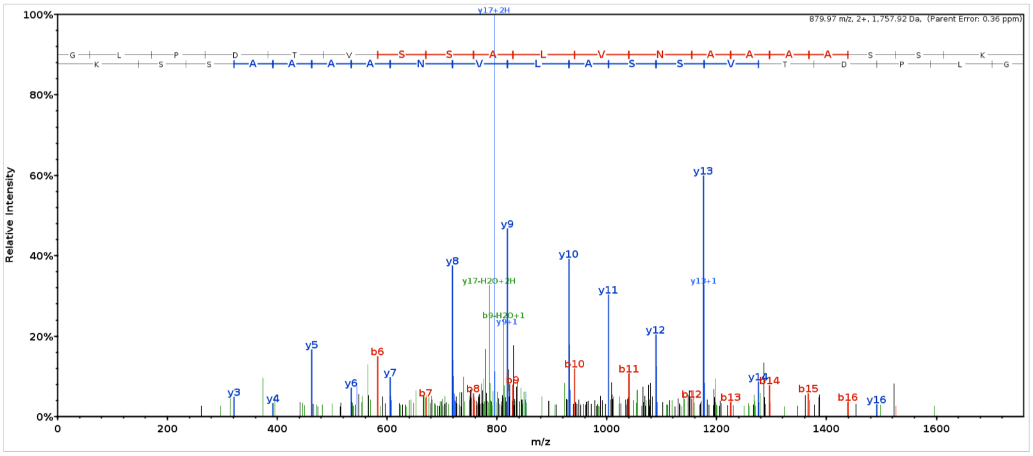

B	B Ions	B+2H	B-NH3	B-H2O	AA	Y Ions	Y+2H	Y-NH3	Y-H2O	Y
1	58.0				G	1,75...	880.0	1,74...	1,74...	19
2	171.1				L	1,70...	851.5	1,68...	1,68...	18
3	268.2				P	1,58...	794.9	1,57...	1,57...	17
4	383.2			365.2	D	1,49...	746.4	1,47...	1,47...	16
5	484.2			466.2	T	1,37...	688.9	1,35...	1,35...	15
6	583.3	292.2		565.3	V	1,27...	638.3	1,25...	1,25...	14
7	670.3	335.7		652.3	S	1,17...	588.8	1,15...	1,15...	13
8	757.4	379.2		739.4	S	1,08...	545.3	1,07...	1,07...	12
9	828.4	414.7		810.4	A	1,00...	501.8	985.5	984.5	11
10	941.5	471.3		923.5	L	931.5	466.3	914.5	913.5	10
11	1,040.6	520.8		1,02...	V	818.4	409.7	801.4	800.4	9
12	1,154.6	577.8	1,137.6	1,13...	N	719.4	360.2	702.3	701.4	8
13	1,225.6	613.3	1,208.6	1,20...	A	605.3	303.2	588.3	587.3	7
14	1,296.7	648.8	1,279.7	1,27...	A	534.3	267.6	517.3	516.3	6
15	1,367.7	684.4	1,350.7	1,34...	A	463.3		446.2	445.2	5
16	1,438.8	719.9	1,421.7	1,42...	A	392.2		375.2	374.2	4
17	1,525.8	763.4	1,508.8	1,50...	S	321.2		304.2	303.2	3
18	1,612.8	806.9	1,595.8	1,59...	S	234.1		217.1	216.1	2
19	1,758.9	880.0	1,741.9	1,74...	K	147.1		130.1		1

D. VSG peptides (1) – 221 variant

Sequence C...	Protein	Accession	Category	Bio Sample	MS/M...	Prob	%Spec	#Pep	#Unique	#Spec	%Cov	m.w.
	Variant surface glycoprotein MITAT 1.2 OS=Trypanosoma brucei brucei PE=1 SV=1	VSM2_TRYBB	Infected	D1_Infected		100%	0.0065%	1	1	2	3.4%	51 kDa
	Variant surface glycoprotein MITAT 1.2 OS=Trypanosoma brucei brucei PE=1 SV=1	VSM2_TRYBB	Infected	D3_Infected		100%	0.0028%	1	1	1	1.9%	51 kDa

Valid	Weight	Sequence	SEQU...	Prob	SEQU...	NTT	Modifications	Observed	Actual Mass	Charge	Delta ...	Delta ...	Reten...	Intens...	TIC	Start	Stop	#...	...	Spectrum ID
☑	1.0	(R)QADAANNFHDNDAECR(L)	2.94	100%	0.79	2	Carbamidomethyl (+57)	616.58	1,846.73	3	0.0036	1.9	1670		33190	199	214	0		Dataset1 Infected-4026-5438_5438
☑	1.0	(R)QADAANNFHDNDAECR(L)	3.63	100%	0.71	2	Carbamidomethyl (+57)	616.59	1,846.74	3	0.0071	3.8	1670		35680	199	214	0		Dataset1 Infected-4025-5436_5436

VSM2_TRYBB (100%), 51,042.2 Da
Variant surface glycoprotein MITAT 1.2 OS=Trypanosoma brucei brucei PE=1 SV=1
1 exclusive unique peptides, 1 exclusive unique spectra, 2 total spectra, 16/476 amino acids (3% coverage)

```
M P S N Q E A R L F   L A V L V L A Q V L   P I L V D S A A E K   G F K Q A F W Q P L   C Q V S E E L D D Q   P K G A L F T L Q A   A A S K I Q K M R D   A A L R A S I Y A E
I N H G T N R A K A   A V I V A N H Y A M   K A D S G L E A L K   Q T L S S Q E V T A   T A T A S Y L K G R   I D E Y L N L L L Q   T K E S G T S G C M   M D T S G T N T V T
K A G G T I G G V P   C K L Q L S P I Q P   K R P A A T Y L G K   A G Y V G L T R Q A   D A A N N F H D N D   A E C R L A S G H N   T N G L G K S G Q L   S A A V T M A A G Y
V T V A N S Q T A V   T V Q A L D A L Q E   A S G A A H Q P W I   D A W K A K K A L T   G A E T A E F R N E   T A G I A G K T G V   T K L V E E A L L K   K K D S E A S E I Q
T E L K K Y F S G H   E N E Q W T A I E K   L I S E Q P V A Q N   L V G D N Q P T K L   G E L E G N A K L T   T I L A Y Y R M E T   A G K F E V L T Q K   H K P A E S Q Q Q A
A E T E G S C N K K   D Q N E C K S P C K   W H N D A E N K K C   T L D K E E A K K V   A D E T A K D G K T   G N T N T T G S S N   S F V I S K T P L W   L A V L L F
```

Peptide: QADAANNFHDNDAECR; Spectrum 1

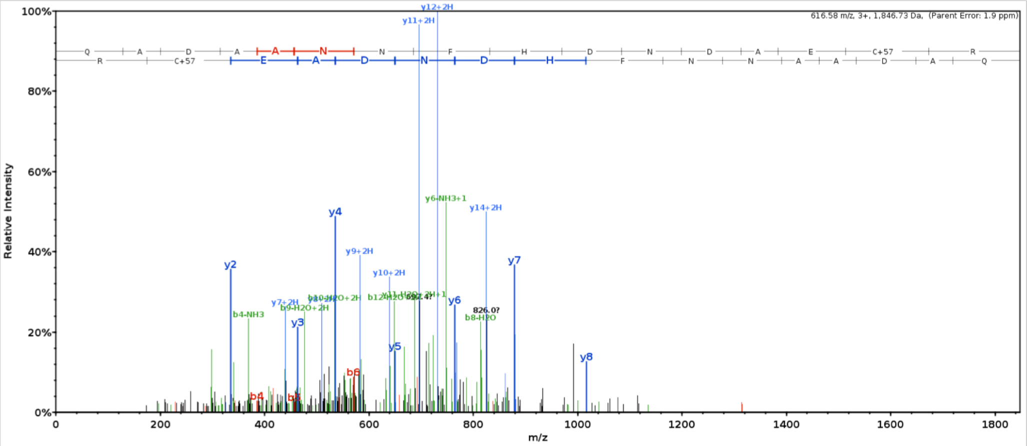

B	B Ions	B+2H	B-NH3	B-H2O	AA	Y Ions	Y+2H	Y-NH3	Y-H2O	Y
1	129.1	65.0	112.0		Q	1,84...	924.4	1,83...	1,82...	16
2	200.1	100.6	183.1		A	1,71...	860.3	1,70...	1,70...	15
3	315.1	158.1	298.1	297.1	D	1,64...	824.8	1,63...	1,63...	14
4	386.2	193.6	369.1	368.2	A	1,53...	767.3	1,51...	1,51...	13
5	457.2	229.1	440.2	439.2	A	1,46...	731.8	1,44...	1,44...	12
6	571.2	286.1	554.2	553.2	N	1,39...	696.3	1,37...	1,37...	11
7	685.3	343.1	668.3	667.3	N	1,27...	639.3	1,26...	1,25...	10
8	832.4	416.7	815.3	814.3	F	1,16...	582.2	1,14...	1,14...	9
9	969.4	485.2	952.4	951.4	H	1,01...	508.7	999.4	998.4	8
10	1,084.4	542.7	1,067.4	1,06...	D	879.3	440.2	862.3	861.3	7
11	1,198.5	599.7	1,181.5	1,18...	N	764.3	382.7	747.3	746.3	6
12	1,313.5	657.3	1,296.5	1,29...	D	650.3	325.6	633.2	632.2	5
13	1,384.6	692.8	1,367.5	1,36...	A	535.2	268.1	518.2	517.2	4
14	1,513.6	757.3	1,496.6	1,49...	E	464.2	232.6	447.2	446.2	3
15	1,673.6	837.3	1,656.6	1,65...	C+57	335.1	168.1	318.1		2
16	1,847.7	924.4	1,830.7	1,82...	R	175.1	88.1	158.1		1

Peptide: QADAANNFHDNDAECR; Spectrum 2

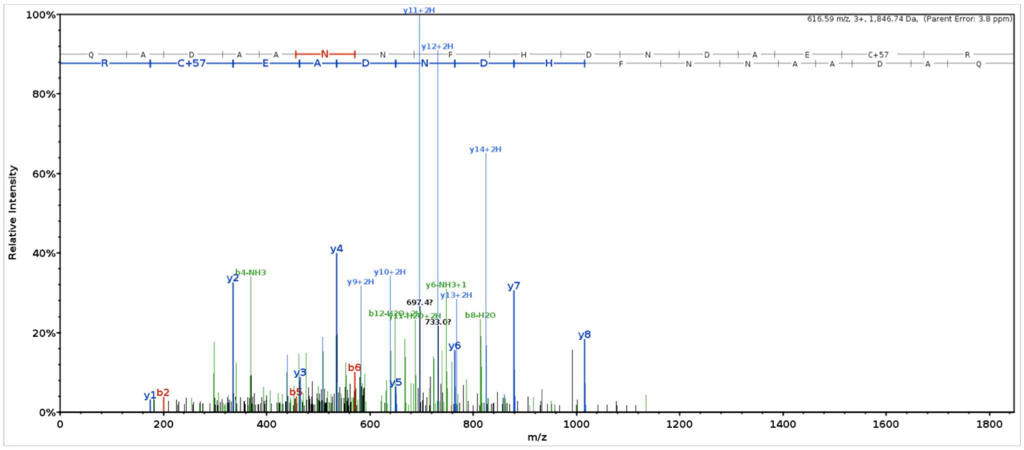

B	B Ions	B+2H	B-NH3	B-H2O	AA	Y Ions	Y+2H	Y-NH3	Y-H2O	Y
1	129.1	65.0	112.0		Q	1,84...	924.4	1,83...	1,82...	16
2	200.1	100.6	183.1		A	1,71...	860.3	1,70...	1,70...	15
3	315.1	158.1	298.1	297.1	D	1,64...	824.8	1,63...	1,63...	14
4	386.2	193.6	369.1	368.2	A	1,53...	767.3	1,51...	1,51...	13
5	457.2	229.1	440.2	439.2	A	1,46...	731.8	1,44...	1,44...	12
6	571.2	286.1	554.2	553.2	N	1,39...	696.3	1,37...	1,37...	11
7	685.3	343.1	668.3	667.3	N	1,27...	639.3	1,26...	1,25...	10
8	832.4	416.7	815.3	814.3	F	1,16...	582.2	1,14...	1,14...	9
9	969.4	485.2	952.4	951.4	H	1,01...	508.7	999.4	998.4	8
10	1,084.4	542.7	1,067.4	1,06...	D	879.3	440.2	862.3	861.3	7
11	1,198.5	599.7	1,181.5	1,18...	N	764.3	382.7	747.3	746.3	6
12	1,313.5	657.3	1,296.5	1,29...	D	650.3	325.6	633.2	632.2	5
13	1,384.6	692.8	1,367.5	1,36...	A	535.2	268.1	518.2	517.2	4
14	1,513.6	757.3	1,496.6	1,49...	E	464.2	232.6	447.2	446.2	3
15	1,673.6	837.3	1,656.6	1,65...	C+57	335.1	168.1	318.1		2
16	1,847.7	924.4	1,830.7	1,82...	R	175.1	88.1	158.1		1

E. VSG peptides (2) – 221 variant

Sequence C...	Protein	Accession	Category	Bio Sample	MS/M...	Prob	%Spec	#Pep	#Unique	#Spec	%Cov	m.w.
	Variant surface glycoprotein MITAT 1.2 OS=Trypanosoma brucei brucei PE=1 SV=1	VSM2_TRYBB	Infected	D1_Infected		100%	0.0065%	1	1	2	3.4%	51 kDa
	Variant surface glycoprotein MITAT 1.2 OS=Trypanosoma brucei brucei PE=1 SV=1	VSM2_TRYBB	Infected	D3_Infected		100%	0.0028%	1	1	1	1.9%	51 kDa

Valid	Weight	Sequence	SEQU...	Prob	SEQU...	NTT	Modifications	Observed	Actual Mass	Charge	Delta ...	Delta ...	Reten...	Intens...	TIC	Start	Stop	#...	...	Spectrum ID
☑	1.0	(K)LQLSPIQPK(R)	2.67	99%	0.44	2		512.31	1,022.61	2	-0.00...	-0.86	3680		111700	173	181	0		Dataset3 Infected saliva-13478-15838...
☑	1.0	(K)DSEASEIQTELK(K)	3.57	93%	0.24	2		675.32	1,348.63	2	-0.0018	-1.4	3750		74310	313	324	0		Dataset3 Infected saliva-13842-162

VSM2_TRYBB (100%), 51,042.2 Da
Variant surface glycoprotein MITAT 1.2 OS=Trypanosoma brucei brucei PE=1 SV=1
2 exclusive unique peptides, 2 exclusive unique spectra, 2 total spectra, 21/476 amino acids (4% coverage)

MPSNQEARLF LAVLVLAQVL PILVDSAAEK GFKQAFWQPL CQVSEELDDQ PKGALFTLQA AASKIQKMRD AALRASIYAE
INHGTNRAKA AVIVANHYAM KADSGLEALK QTLSSQEVTA TATASYLKGR IDEYLNLLLQ TKESGTSGCM MDTSGTNTVT
KAGGTIGGVP CK**LQLSPIQP** **K**RPAATYLGK AGYVGLTRQA DAANNFHDND AECRLASGHN TNGLGKSGQL SAAVTMAAGY
VTVANSQTAV TVQALDALQE ASGAAHQPWI DAWKAKKALT GAETAEFRNE TAGIAGKTGV TKLVEEALLK KK**DSEASEIQ**
TELKKYFSGH ENEQWTAIEK LISEQPVAQN LVGDNQPTKL GELEGNAKLT TILAYYRMET AGKFEVLTQK HKPAESQQQA
AETEGSCNKK DQNECKSPCK WHNDAENKKC TLDKEEAKKV ADETAKDGKT GNTNTTGSSN SFVISKTPLW LAVLLF

Peptide: LQLSPIQPK; Spectrum 1

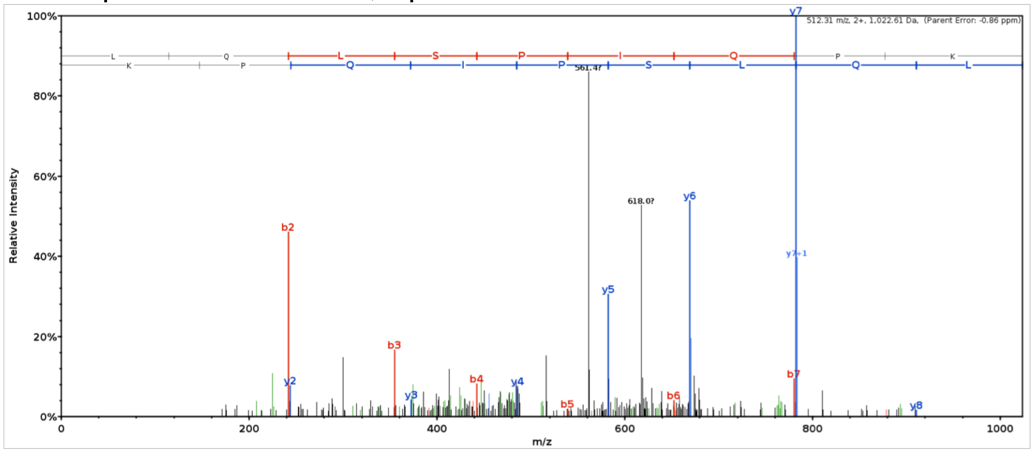

B	B Ions	B+2H	B-NH3	B-H2O	AA	Y Ions	Y+2H	Y-NH3	Y-H2O	Y
1	114.1				L	1,02...	512.3	1,00...	1,00...	9
2	242.1		225.1		Q	910.5	455.8	893.5	892.5	8
3	355.2		338.2		L	782.5	391.7	765.5	764.5	7
4	442.3		425.2	424.3	S	669.4	335.2	652.4	651.4	6
5	539.3		522.3	521.3	P	582.4		565.3		5
6	652.4	326.7	635.4	634.4	I	485.3		468.3		4
7	780.5	390.7	763.4	762.5	Q	372.2		355.2		3
8	877.5	439.3	860.5	859.5	P	244.2		227.1		2
9	1,023.6	512.3	1,006.6	1,00...	K	147.1		130.1		1

Peptide: DSEASEIQTELK; Spectrum 1

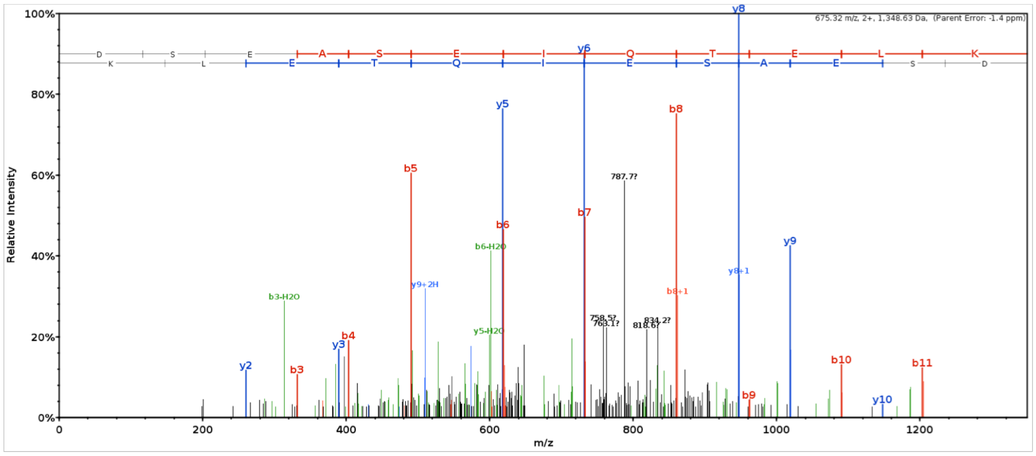

B	B Ions	B+2H	B-NH3	B-H2O	AA	Y Ions	Y+2H	Y-NH3	Y-H2O	Y
1	116.0			98.0	D	1,34...	675.3	1,33...	1,33...	12
2	203.1			185.1	S	1,23...	617.8	1,21...	1,21...	11
3	332.1			314.1	E	1,14...	574.3	1,13...	1,12...	10
4	403.1			385.1	A	1,01...	509.8	1,00...	1,00...	9
5	490.2			472.2	S	947.5	474.3	930.5	929.5	8
6	619.2	310.1		601.2	E	860.5	430.7	843.4	842.5	7
7	732.3	366.7		714.3	I	731.4	366.2	714.4	713.4	6
8	860.4	430.7	843.3	842.4	Q	618.3		601.3	600.3	5
9	961.4	481.2	944.4	943.4	T	490.3		473.3	472.3	4
10	1,090.5	545.7	1,073.4	1,07...	E	389.2		372.2	371.2	3
11	1,203.5	602.3	1,186.5	1,18...	L	260.2		243.2		2
12	1,349.6	675.3	1,332.6	1,33...	K	147.1		130.1		1

F. VSG (3) – mVAT4

Sequence C...	Protein	Accession	Category	Bio Sample	MS/M...	Prob	%Spec	#Pep	#Unique	#Spec	%Cov	m.w.
	MVAT4 variant surface glycoprotein OS=Trypanosoma brucei rhodesiense GN=MVT...	O76421_TRYBR	Infected	D1_Infected		100%	0.0033%	1	1	1	3.3%	51 kDa

Valid	Weight	Sequence	SEQU...	Prob	SEQU...	NTT	Modifications	Observed	Actual Mass	Charge	Delta ...	Delta ...	Reten...	Intens...	TIC	Start	Stop	#...	...	Spectrum ID
☑	1.0	(R)EAADVPCVNNGGGLNK(I)	2.82	100%	0.52	2	Carbamidomethyl (+57)	807.88	1,613.75	2	0.0016	0.97	2860		17450	154	169	0		Dataset1 Infected-8830-10903_10903

O76421_TRYBR (100%), 50,880.3 Da
MVAT4 variant surface glycoprotein OS=Trypanosoma brucei rhodesiense GN=MVTA4-vsg PE=4 SV=1
1 exclusive unique peptides, 1 exclusive unique spectra, 1 total spectra, 16/479 amino acids (3% coverage)

M P T A G A V L L L   A T A A V L S R P A   A A A N E K K P L T   I S A A G A V C G F   S N E L K E V A S F   A A T K V N A Y L T   E T E Q L G T L A V   D L S A A I F N G N
G K P T A G E V Y A   A L L A A K T Q T D   R Q A Q Q Q K L V T   Q A L L A S S L C S   Q Q A G H I E S F I   H V F Y Q A H R D A   T T T C I Y K S D E   H Q R **E A A D V P C**
V N N G G G L N K I   T I T T R A T K P Q   L N S K Y K A I V A   G T T P N G A A A E   K N C K L V N G E Q   S Q N V F L A S Q T   T N I A L K W G D G   L L T V P I G D T A
I S A S N W E P N N   K D S I A S G N Y K   E C A A A L E Q V A   A A A E Y S S A A T   S K L L K L E A E D   D P K L E E E L I S   K N S F Y G D F P I   S N V K I T A T D F
S S L H S K L K S Y   R K K H T A D G D Q   L L K A R L Q H L E   K Q M Q M N A T A C   K L G A E I S S G E   P S T A K T T T S G   R C E G K A K T A C   P K S D C Q W E E K
D G K G E C K P K S   G E E Q K T Q T T G   A G E G A A D K K E   E K C K G K L E P E   C T K A P E C K W E   G E T C K D S S I L   V N K Q F T L S M I   S A A F M A L L F

Peptide: EAADVPCVNNGGGLNK; Spectrum 1

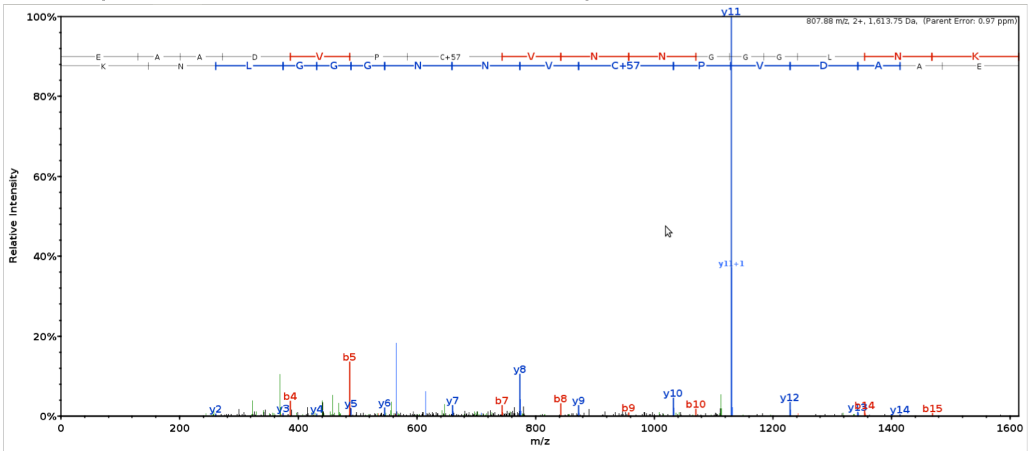

B	B Ions	B+2H	B-NH3	B-H2O	AA	Y Ions	Y+2H	Y-NH3	Y-H2O	Y
1	130.0			112.0	E	1,61...	807.9	1,59...	1,59...	16
2	201.1			183.1	A	1,48...	743.4	1,46...	1,46...	15
3	272.1			254.1	A	1,41...	707.8	1,39...	1,39...	14
4	387.2			369.1	D	1,34...	672.3	1,32...	1,32...	13
5	486.2			468.2	V	1,22...	614.8	1,21...		12
6	583.3	292.1		565.3	P	1,12...	565.3	1,11...		11
7	743.3	372.2		725.3	C+57	1,03...	516.7	1,01...		10
8	842.4	421.7		824.4	V	872.5	436.7	855.4		9
9	956.4	478.7	939.4	938.4	N	773.4	387.2	756.4		8
10	1,070.5	535.7	1,053.4	1,05...	N	659.3	330.2	642.3		7
11	1,127.5	564.2	1,110.5	1,10...	G	545.3	273.2	528.3		6
12	1,184.5	592.8	1,167.5	1,16...	G	488.3		471.3		5
13	1,241.5	621.3	1,224.5	1,22...	G	431.3		414.2		4
14	1,354.6	677.8	1,337.6	1,33...	L	374.2		357.2		3
15	1,468.6	734.8	1,451.6	1,45...	N	261.2		244.1		2
16	1,614.8	807.9	1,597.7	1,59...	K	147.1		130.1		1

G. VSG (4) – 1125.4959 variant

Sequence C...	Protein	Accession	Category	Bio Sample	MS/M...	Prob	%Spec	#Pep	#Unique	#Spec	%Cov	m.w.
	Variant surface glycoprotein 1125.4959 OS=Trypanosoma brucei PE=4 SV=1	A0A1J0RB71_9TRYP	Infected	D1_Infected		95%	0.0033%	1	1	1	2.1%	44 kDa

Valid	Weight	Sequence	SEQU...	Prob	SEQU...	NTT	Modifications	Observed	Actual Mass	Charge	Delta ...	Delta ...	Reten...	Intens...	TIC	Start	Stop	#...	...	Spectrum ID
☑	1.0	(K)LQQQLALYR(V)	2.57	99%	0.32	2		566.83	1,131.64	2	0.000...	0.81	3310		68910	69	77	0		Dataset1 Infected-10776-13014_13014

A0A1J0RB71_9TRYP (95%), 44,493.5 Da
Variant surface glycoprotein 1125.4959 OS=Trypanosoma brucei PE=4 SV=1
1 exclusive unique peptides, 1 exclusive unique spectra, 1 total spectra, 9/421 amino acids (2% coverage)

M K Q H L V F A A A   A A I I S L A P A A   H V E A A A G D A L   N H A A W S K L C D   I T R D L D N L P S   N E L A S I A A E Q   V N V G D L T K **L Q**   **Q Q L A L Y R** V L N
T D K A A T T A E Q   V F A T F L S R K I   S G A A I S E T K L   K E A L Q S T A D A   A F L H G Q L A E W   L A T A S S I G G N   T A G C L G A A G G   A D T A D K A T I T
Q A P Y A C K L T T   Q M A S A K L T T P   A S I D A N G Y T G   F T A I P N V E T S   N A V T Q K K C A Y   S Q H G A S G L G G   A Q D T T T S I A F   A G G A F I L T D N
A L T R S N W A A T   S Q A G T L P H E K   A F K A L K R Q H K   S L P K A N Y T K I   T D L K Q D S D F R   S A A I A I I Y G D   Q D S S Q L E N K L   T D L F G A S D N S
F E E K F W K N V R   E A K I D G G K F G   A K E A T T I Q A L   E D P I K L S K A F   Y Y Y A K Q G T E Q   L K T L E A R A A A   T N D L P K P T E E   L C N A K N D E P K
A C N E A T G C H Y   D A S K Q K G Q N V   L

Peptide: LQQQLALYR; Spectrum 1

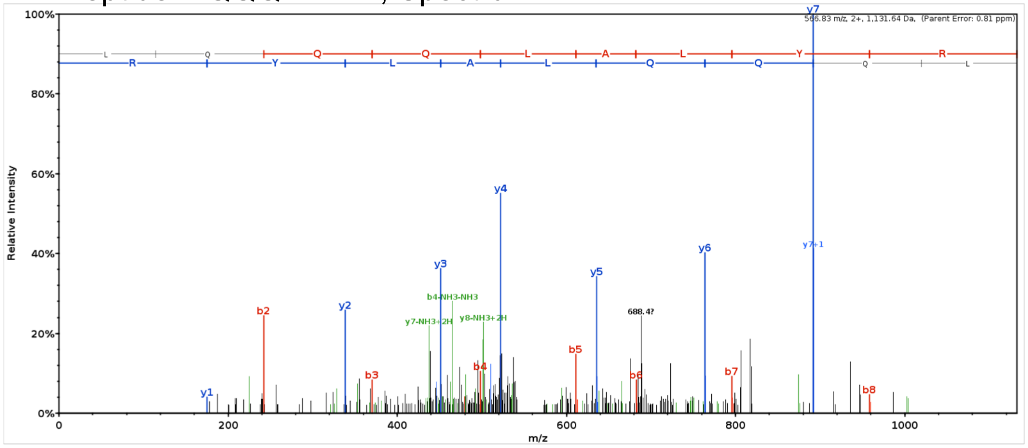

B	B Ions	B+2H	B-NH3	B-H2O	AA	Y Ions	Y+2H	Y-NH3	Y-H2O	Y
1	114.1				L	1,13...	566.8	1,11...	1,11...	9
2	242.1		225.1		Q	1,01...	510.3	1,00...	1,00...	8
3	370.2		353.2		Q	891.5	446.3	874.5		7
4	498.3		481.2		Q	763.4	382.2	746.4		6
5	611.4		594.3		L	635.4		618.4		5
6	682.4	341.7	665.4		A	522.3		505.3		4
7	795.5	398.2	778.4		L	451.3		434.2		3
8	958.5	479.8	941.5	940.5	Y	338.2		321.2		2
9	1,132.6	566.8	1,115.6	1,11...	R	175.1		158.1		1

H. VSG (5) – 1125.3088 variant

Sequence C...	Protein	Accession	Category	Bio Sample	MS/M...	Prob	%Spec	#Pep	#Unique	#Spec	%Cov	m.w.
	Variant surface glycoprotein 1125.3088 OS=Trypanosoma brucei PE=4 SV=1	A0A1J0R978_9TRYP	Infected	D1_Infected		97%	0.0033%	1	1	1	2.1%	46 kDa

Valid	Weight	Sequence	SEQU...	Prob	SEQU...	NTT	Modifications	Observed	Actual Mass	Charge	Delta ...	Delta ...	Reten...	Intens...	TIC	Start	Stop	#...	...	Spectrum ID
☑	1.0	(K)STEELCNAK(K)	2.49	99%	0.45	2	Carbamidomethyl (+57)	526.24	1,050.47	2	0.000...	0.069	1200		30080	317	325	0		Dataset1 Infected-2340-3416_3416

A0A1J0R978_9TRYP (97%), 45,950.0 Da
Variant surface glycoprotein 1125.3088 OS=Trypanosoma brucei PE=4 SV=1
1 exclusive unique peptides, 1 exclusive unique spectra, 1 total spectra, 9/426 amino acids (2% coverage)

```
M F S I V L S L A F   L V L T T M K K T E   A A A A A F L Q G Q   Q A G F I T T M E N   L V S S T G A S S G   G C L Q A T Q G G S   D A V N G A S T L K   N C G L N G R N D E
A K E E D P T N Y G   L T K L F N H L P A   A A I T D A T G T P   A G K C L L T K I A   T G S G L L S D Q D   T P S D V A M A G N   F I I S K K H P G G   A E V K T A H N K A
T G Q L T P D I P L   L T Q T R Q K M I T   F D S H S F L T A N   T Y S R P T L A E V   K A D K R S K V C A   K L Y L Q N K N D A   Y D D K A D K A G A   E T A I A A A Y G K
G T E I D A A K T W   T K I E N E H V D K   K A Y M E D A T G T   E P L S K V K D I E   H L L K V L A Y Y N   E K T F N E L N E K   I K D L E E K S K T   N K D F P K S T E E
L C N A K K D N A K   A C N E T T G C H Y   D A S K T E G R R C   T L K P E V K A Q L   Q E T N Q E T G G N   D G K T E E K C A K   H G S D K N A C D N   D N N C K W E N N A
C K D S S F I L K K   K F S L R M A A A F   V G M V V F
```

Peptide: STEELCNAK; Spectrum 1

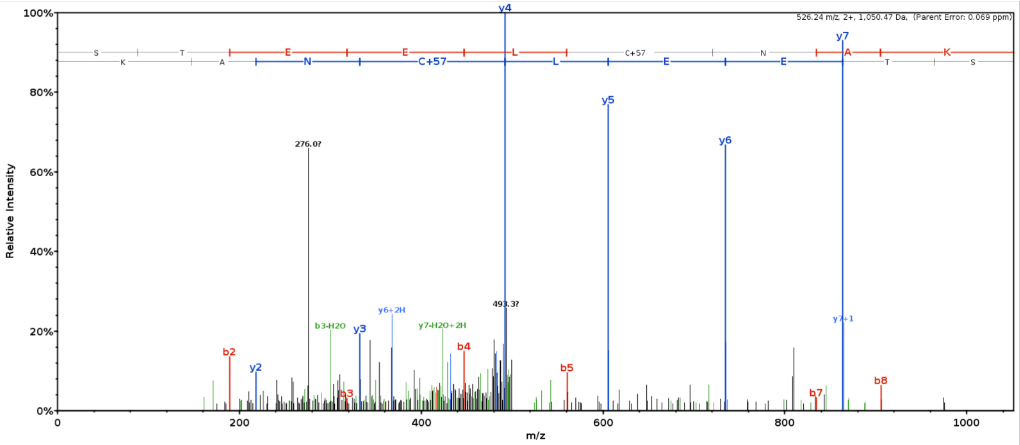

B	B Ions	B+2H	B-NH3	B-H2O	AA	Y Ions	Y+2H	Y-NH3	Y-H2O	Y
1	88.0			70.0	S	1,05...	526.2	1,03...	1,03...	9
2	189.1			171.1	T	964.4	482.7	947.4	946.4	8
3	318.1			300.1	E	863.4	432.2	846.4	845.4	7
4	447.2			429.2	E	734.4	367.7	717.3	716.3	6
5	560.3			542.2	L	605.3		588.3		5
6	720.3	360.6		702.3	C+57	492.2		475.2		4
7	834.3	417.7	817.3	816.3	N	332.2		315.2		3
8	905.4	453.2	888.3	887.4	A	218.1		201.1		2
9	1,051.5	526.2	1,034.4	1,03...	K	147.1		130.1		1

I. VSG (6) – 1125.408 variant

Sequence C...	Protein	Accession	Category	Bio Sample	MS/M...	Prob	%Spec	#Pep	#Unique	#Spec	%Cov	m.w.
	Variant surface glycoprotein 1125.408 OS=Trypanosoma brucei PE=4 SV=1	A0A1J0R4A6_9TRYP	Infected	D3_Infected		99%	0.0083%	1	2	3	2.3%	49 kDa

Valid	Weight	Sequence	SEQU...	Prob	SEQU...	NTT	Modifications	Observed	Actual Mass	Charge	Delta ...	Delta ...	Reten...	Intens...	TIC	Start	Stop	#...	...	Spectrum ID
☑	1.0	(K)KTDAADKTEEK(I)	3.15	100%	0.39	2		412.54	1,234.60	3	-0.00...	-0.19	725		116200	430	440	0		Dataset3 Infected saliva-499-1265_12...
☑	1.0	(K)KTDAADKTEEK(I)	3.18	99%	0.40	2		618.31	1,234.60	2	-0.00...	-0.42	737		19230	430	440	0		Dataset3 Infected saliva-525-1305_13...
☑	1.0	(K)KTDAADKTEEK(I)	2.97	99%	0.34	2		618.31	1,234.60	2	-0.00...	-0.12	740		18840	430	440	0		Dataset3 Infected saliva-529-1312_13...

A0A1J0R4A6_9TRYP (99%), 49,359.8 Da
Variant surface glycoprotein 1125.408 OS=Trypanosoma brucei PE=4 SV=1
1 exclusive unique peptides, 2 exclusive unique spectra, 3 total spectra, 11/471 amino acids (2% coverage)

```
M A A G L G F Q Q P   I V F L L A T A T L   T H A S K S T P P A   N A A V V D F C T E   A D Y G D S I V A E   L T E W T A T E T E   N L R T L A D D A A   M F Q L A A T R Y A
G H P E G L G F K T   L A A I A A N R L T   K Q S Q T I K E D L   K N Q I A A I E I L   S R R M A E L A T L   H A A E T A T V T T   G T S G K A T T A S   H P I A E A E A S K
R C E I T T T T T A   K K T T T C Q P K G   S S G A D P R A I R   Q Q L Q Q G S E V A   I R E P A T L L T R   K Y E L L V G V K G   T L S S G G T W A T   F N D A P G C T D D
S S A G N I G A E T   N V V L A S L Q T A   G E R P S F T K G R   I D T A G P T Q T A   P A A T E S G A N Q   R R L F T T T D C E L   A A A L K A A L K H   K P G P V P A L S E
V I E T V I G Q L E   G Q A A A R G V L L   A T G K A A D K S L   S A D E V A Q L V F   N V K P R E I K T K   F I D N L A A D S I   T I N A A H L A I K   G T T K D L S E T N
F D E A Q V Y F Y A   K N Q K K A A A A S   V G A K N E G E K K   T D A A D K T E E K   I D G D N K T T T N   T T G S N S F V I N   R A P L L L A F L L   F
```

Peptide: KTDAADKTEEK; Spectrum 1

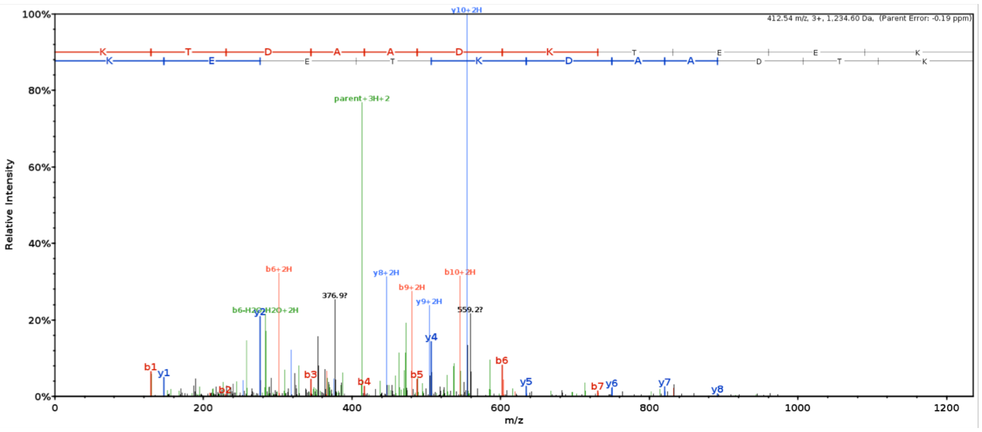

B	B Ions	B+2H	B-NH3	B-H2O	AA	Y Ions	Y+2H	Y-NH3	Y-H2O	Y
1	129.1	65.1	112.1		K	1,23...	618.3	1,21...	1,21...	11
2	230.1	115.6	213.1	212.1	T	1,10...	554.3	1,09...	1,08...	10
3	345.2	173.1	328.2	327.2	D	1,00...	503.7	989.4	988.5	9
4	416.2	208.6	399.2	398.2	A	891.4	446.2	874.4	873.4	8
5	487.3	244.1	470.2	469.2	A	820.4	410.7	803.4	802.4	7
6	602.3	301.6	585.3	584.3	D	749.4	375.2	732.3	731.4	6
7	730.4	365.7	713.3	712.4	K	634.3	317.7	617.3	616.3	5
8	831.4	416.2	814.4	813.4	T	506.2	253.6	489.2	488.2	4
9	960.5	480.7	943.4	942.5	E	405.2	203.1	388.2	387.2	3
10	1,089.5	545.3	1,072.5	1,07...	E	276.2	138.6	259.1	258.1	2
11	1,235.6	618.3	1,218.6	1,21...	K	147.1	74.1	130.1		1

J. MISP peptides

Sequence C...	Protein	Accession	Category	Bio Sample	MS/M...	Prob	%Spec	#Pep	#Unique	#Spec	%Cov	m.w.
	Uncharacterized protein OS=Trypanosoma brucei brucei (strain 927/4 GUTat10.1) GN=Tb927.7.380 PE=4 SV=1	Q57U16_TRYB2	Infected	D1_Infected		100%	0.0065%	1	1	2	3.7%	38 kDa

Valid	Weight	Sequence	SEQU...	Prob	SEQU...	NTT	Modifications	Observed	Actual Mass	Charge	Delta ...	Delta ...	Reten...	Intens...	TIC	Start	Stop	#...	...	Spectrum ID
☑	1.0	(K)SVAEDNSAASTAR(R)	3.71	100%	0.65	2		639.80	1,277.59	2	0.0015	1.2	1180		20290	139	151	0		Dataset1 Infected-2262-3332_3332
☑	1.0	(K)SVAEDNSAASTAR(R)	3.51	100%	0.64	2		639.80	1,277.59	2	0.000...	0.50	1200		12830	139	151	0		Dataset1 Infected-2359-3436_3436

Q57U16_TRYB2 (100%), 38,262.9 Da
Uncharacterized protein OS=Trypanosoma brucei brucei (strain 927/4 GUTat10.1) GN=Tb927.7.380 PE=4 SV=1
1 exclusive unique peptides, 1 exclusive unique spectra, 2 total spectra, 13/356 amino acids (4% coverage)

```
MTARFLCLLA ILTYVTADSI IEEGSQNTVS HVSAACLFSE ALHGIPFGVK ALKALAAANV SDASKAREGC QDAVRRAEDA
SWNNPKAEEA VGRARAALKE AESAENAAKT ALSDVEQYAA RAPLLAAGKT APIDDYLKSV AEDNSAASTA RRIARGCSLP
NRGVNSWVLT QAVESACAIF MGEICRIART RMVDLRAEYD QLEAAVRRAG EARVAARAAE SNARKAAEEA ERTAETEEAD
VQEISREEFE GNVEVPEDKE KTGRTEVEEV PKKDPEGNVE VPEDKEKTER TEVEEVPKKD PEGKVEISDD DHVQELGDDE
GEEAEVGNDG SYAESIGGYT LLILLAALFH SAAAHF
```

Peptide: SVAEDNSAASTAR; Spectrum 1

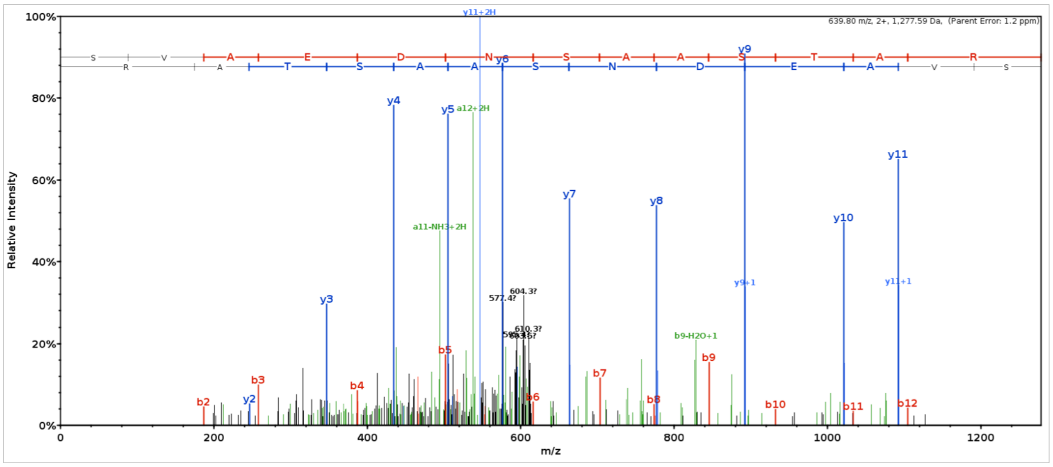

B	B Ions	B+2H	B-NH3	B-H2O	AA	Y Ions	Y+2H	Y-NH3	Y-H2O	Y
1	88.0			70.0	S	1,27...	639.8	1,26...	1,26...	13
2	187.1			169.1	V	1,19...	596.3	1,17...	1,17...	12
3	258.1			240.1	A	1,09...	546.7	1,07...	1,07...	11
4	387.2			369.2	E	1,02...	511.2	1,00...	1,00...	10
5	502.2			484.2	D	892.4	446.7	875.4	874.4	9
6	616.3	308.6	599.2	598.2	N	777.4	389.2	760.4	759.4	8
7	703.3	352.1	686.3	685.3	S	663.3	332.2	646.3	645.3	7
8	774.3	387.7	757.3	756.3	A	576.3	288.7	559.3	558.3	6
9	845.4	423.2	828.3	827.4	A	505.3		488.2	487.3	5
10	932.4	466.7	915.4	914.4	S	434.2		417.2	416.2	4
11	1,033.4	517.2	1,016.4	1,01...	T	347.2		330.2	329.2	3
12	1,104.5	552.7	1,087.5	1,08...	A	246.2		229.1		2
13	1,278.6	639.8	1,261.6	1,26...	R	175.1		158.1		1

Peptide: SVAEDNSAASTAR; Spectrum 2

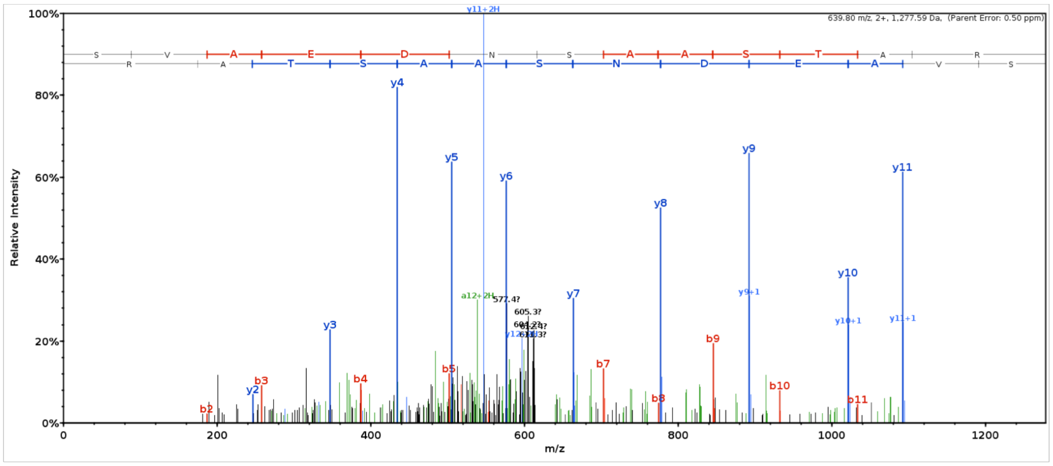

Protein Sequence	Similar Proteins	Spectrum	Spectrum/Model Error	Fragmentation Table						
B	B Ions	B+2H	B-NH3	B-H2O	AA	Y Ions	Y+2H	Y-NH3	Y-H2O	Y
1	88.0			70.0	S	1,27...	639.8	1,26...	1,26...	13
2	187.1			169.1	V	1,19...	596.3	1,17...	1,17...	12
3	258.1			240.1	A	1,09...	546.7	1,07...	1,07...	11
4	387.2			369.2	E	1,02...	511.2	1,00...	1,00...	10
5	502.2			484.2	D	892.4	446.7	875.4	874.4	9
6	616.3	308.6	599.2	598.2	N	777.4	389.2	760.4	759.4	8
7	703.3	352.1	686.3	685.3	S	663.3	332.2	646.3	645.3	7
8	774.3	387.7	757.3	756.3	A	576.3	288.7	559.3	558.3	6
9	845.4	423.2	828.3	827.4	A	505.3		488.2	487.3	5
10	932.4	466.7	915.4	914.4	S	434.2		417.2	416.2	4
11	1,033.4	517.2	1,016.4	1,01...	T	347.2		330.2	329.2	3
12	1,104.5	552.7	1,087.5	1,08...	A	246.2		229.1		2
13	1,278.6	639.8	1,261.6	1,26...	R	175.1		158.1		1
