# Supplementary material for: The Trypanosoma brucei MISP family of invariant proteins is co-expressed with BARP as triple helical bundle structures on the surface of salivary gland forms, but is dispensable for parasite development within the tsetse vector
Source: PLoS Pathog. 2023 Mar 30;19(3):e1011269. doi: 10.1371/journal.ppat.1011269 (PMC10089363; doi:10.1371/journal.ppat.1011269)
Supplement: S2 File — (PDF) [file ppat.1011269.s025.pdf]

Immunization experiment

Vaccination schedule

D -5	D0	D10	D21	D30	D33	D37
Pre-immune serum collection	Immunization	Booster	Booster	Anti- serum collection	Challenge	Daily follow-up parasitemia

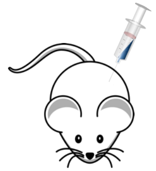

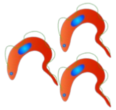

Dead *T. brucei* metacyclics

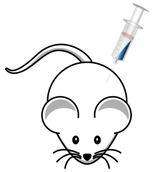

RecMISP

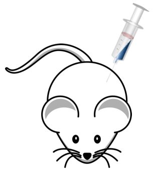

RecBARP

+ 2 ctrl groups : PBS & L(MLPA)

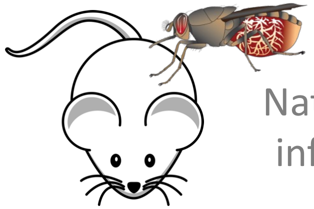

Natural transmission by infected tsetse fly bite

Western blot – analysis IgG response

Credits: open-source images (mice & syringes) were obtained from https://openclipart.org/. Tsetse fly and trypanosomes were drawn by AC-S.

Western blot analysis

Materials & Methods

GEL: BIO-RAD miniprotean TCX precast gel 10%
Ladder: BIO-RAD precision Plus Protein Dual Colour Standard
Running buffer: 80mL TG 10X + 8mL SDS 0.1% + 710mL H2O
TG: 10 x Tris-glycine → 30gr Trizmabase + 144 gr Glycine + 1L H20
Transfer buffer TGM: 120mL TG 10x + 240mL methanol +840mL H20
10 x TBS: 87.6g NaCl + 60.6g Tris in 800 mL H2O – dissolve, add H20 up to 1mL, adjust pH to 7-6 with HCl
TBS-tween 0.05%: 500mL TBS +250µL Tween 20
Blocking buffer: TBS-T 5% skimmed milk: 50mL TBS-T + 2.5gr skimmed milk
TMB-membrane peroxidase substrate

Samples (recMISP, recBARP and whole frozen Tbb metacyclics) were prepared with 4xsodium dodecyl sulfate–polyacrylamide gel electrophoresis (SDS–PAGE) sample loading buffer and heated to 95 °C for 5 min before loading 10µL onto the 10% gel used for SDS-page. The gel was run for 45 min in 0,02mAmp. Next, the samples are blotted onto nitrocellulose membrane (Cytiva) using 75V for 90 min. The blots were cut in separate lanes and probed with anti-serum (1:200 dilution in TBS-T blocking buffer) from the mice immunized with recMISP, recBARp, whole metacyclics and control groups during 1h at RT. After three washes with TBS-T, anti-mouse IgG HRP secondary antibody was applied at 1:20000 dilution and incubated for 1hr ar RT. Bound antibodies were detected by using the TMB-membrane peroxidase substrate (Seracare).

Western blot analysis

Anti-MISP antibody response

Western blot set-up:
recMISP-protein - 95ng /lane (estimate was based upon the concentration mentioned on the vial)
Serum - 1:200 dilution
Anti-mouse IgG HRP - 1:20000 dilution

Samples: we tested 3 mice immunized with recMISP as a try-out

Results:

All three selected mice show an IgG response towards recMISP. The adjuvant control(Ctrla) remained negative

However, in follow-up experiments (> 2 months later) no signal for MISP was observed.
Follow-up experiments: >> loading in the gel: no signal; also no stained band observed after Coomassie; only very faint band after loading 2.5 µg recMISP
Possible explanation: MISP was kept stored at 4°C; maybe not stable and degraded and/or sticking to the tube wall → << recMISP in solution.

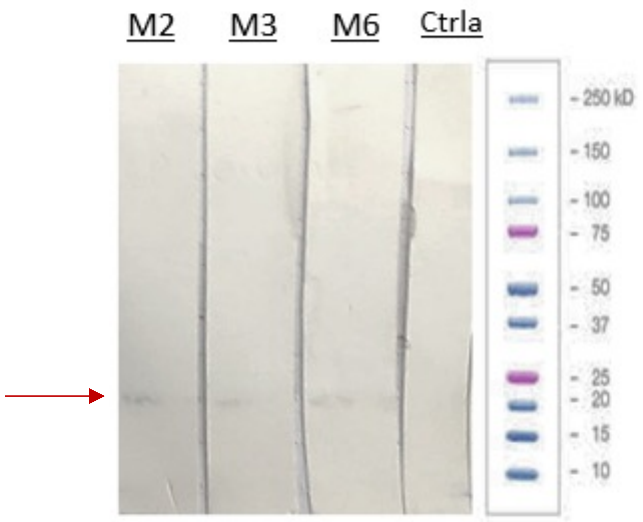

Western blot analysis

Anti-BARP antibody response

Goal: Document the anti-BARP IgG response and background IgG by Western blot analysis.

Western blot set-up:
recBARP-protein - 95ng/lane
Serum - 1:200 dilution
Anti-mouse IgG HRP - 1:20000 dilution

Samples: mice immunized with

Group B: control adjuvants – L(MLPA)
Group C: recMISP
Group D: recBARP
Group E: Whole *T.brucei* metacyclics collected from infected tsetse flies

Anti-BARP antibody response - results

Group B: control adjuvants – L(MLPA)

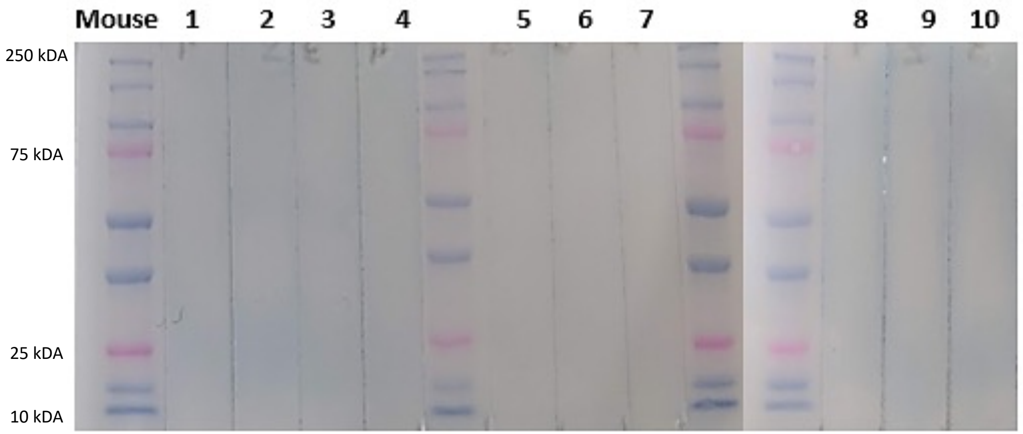

Results: The control group injected with L(MLPA) shows no IgG response towards recBARP

Anti-BARP antibody response - results

Group C: RecMISP

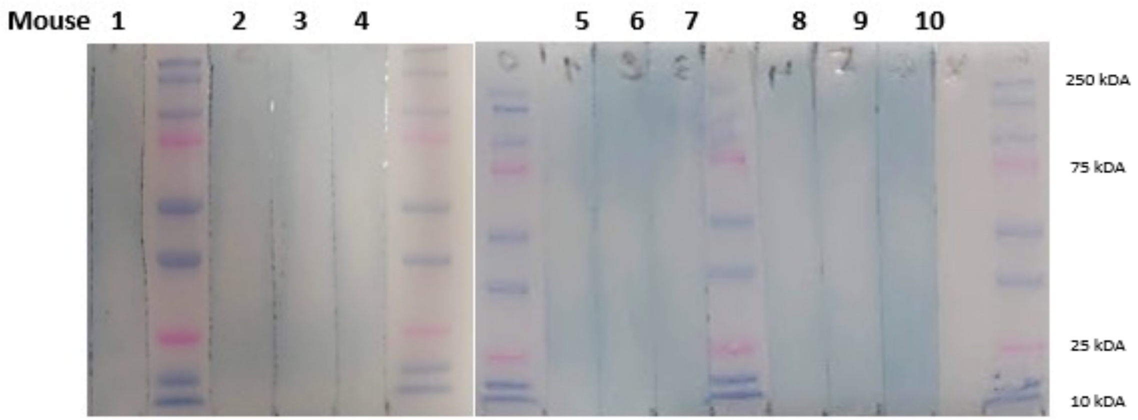

Results: The group immunized with recMISP shows no IgG response towards recBARP

Anti-BARP antibody response - results

Group D: RecBARP

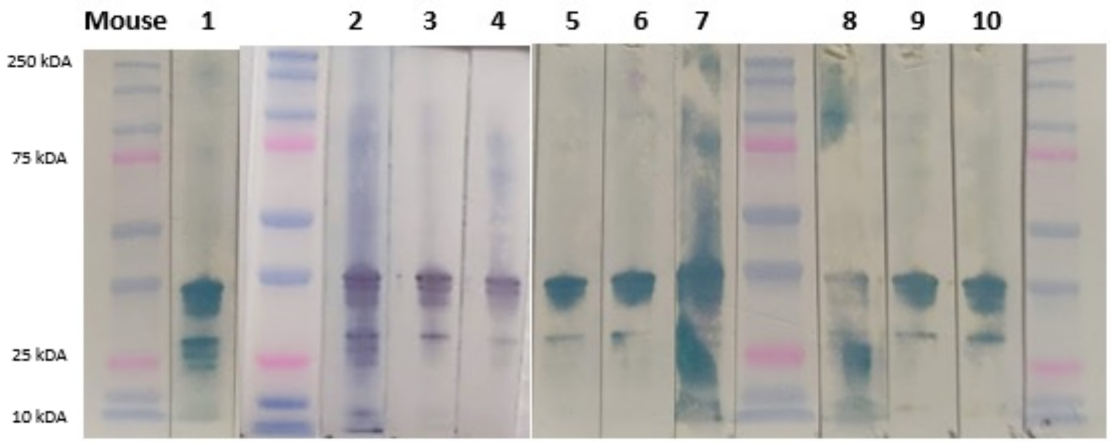

Results: The group immunized with recBARP shows a strong IgG response towards recBARP.

Anti-BARP antibody response - results

Group E: Whole *T. brucei* metacyclics collected from infected tsetse flies

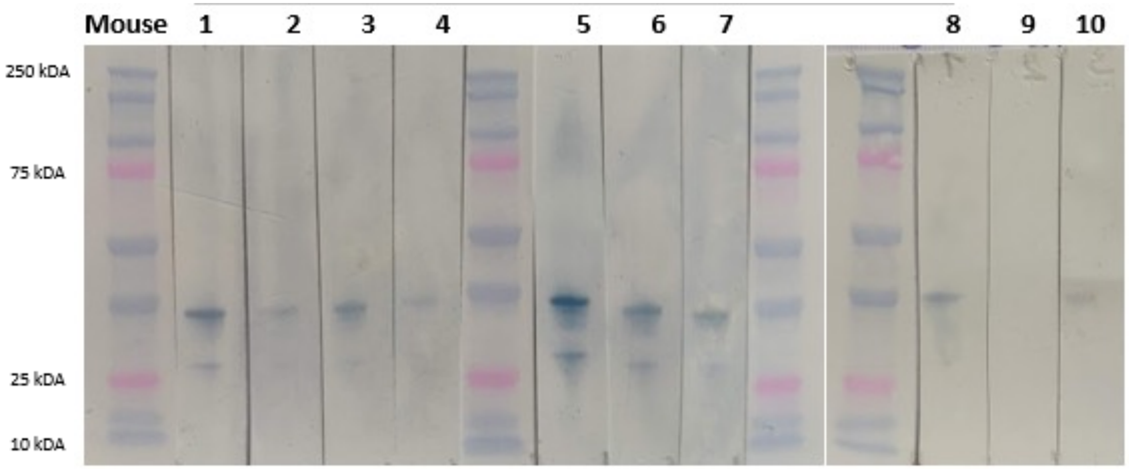

Results: The group immunized with whole *T.b.b* metacyclics shows an IgG response towards recBARP, except for mouse number 9.
