# Supplementary material for: The Trypanosoma brucei MISP family of invariant proteins is co-expressed with BARP as triple helical bundle structures on the surface of salivary gland forms, but is dispensable for parasite development within the tsetse vector
Source: PLoS Pathog. 2023 Mar 30;19(3):e1011269. doi: 10.1371/journal.ppat.1011269 (PMC10089363; doi:10.1371/journal.ppat.1011269)
Supplement: S3 File — (PDF) [file ppat.1011269.s026.pdf]

Metacyclic Invariant Surface Proteins (MISP)

Tb927.7.360; Tb927.7.380; Tb927.7.400; Tb927.7.420; Tb927.7.440; (identified as a group)

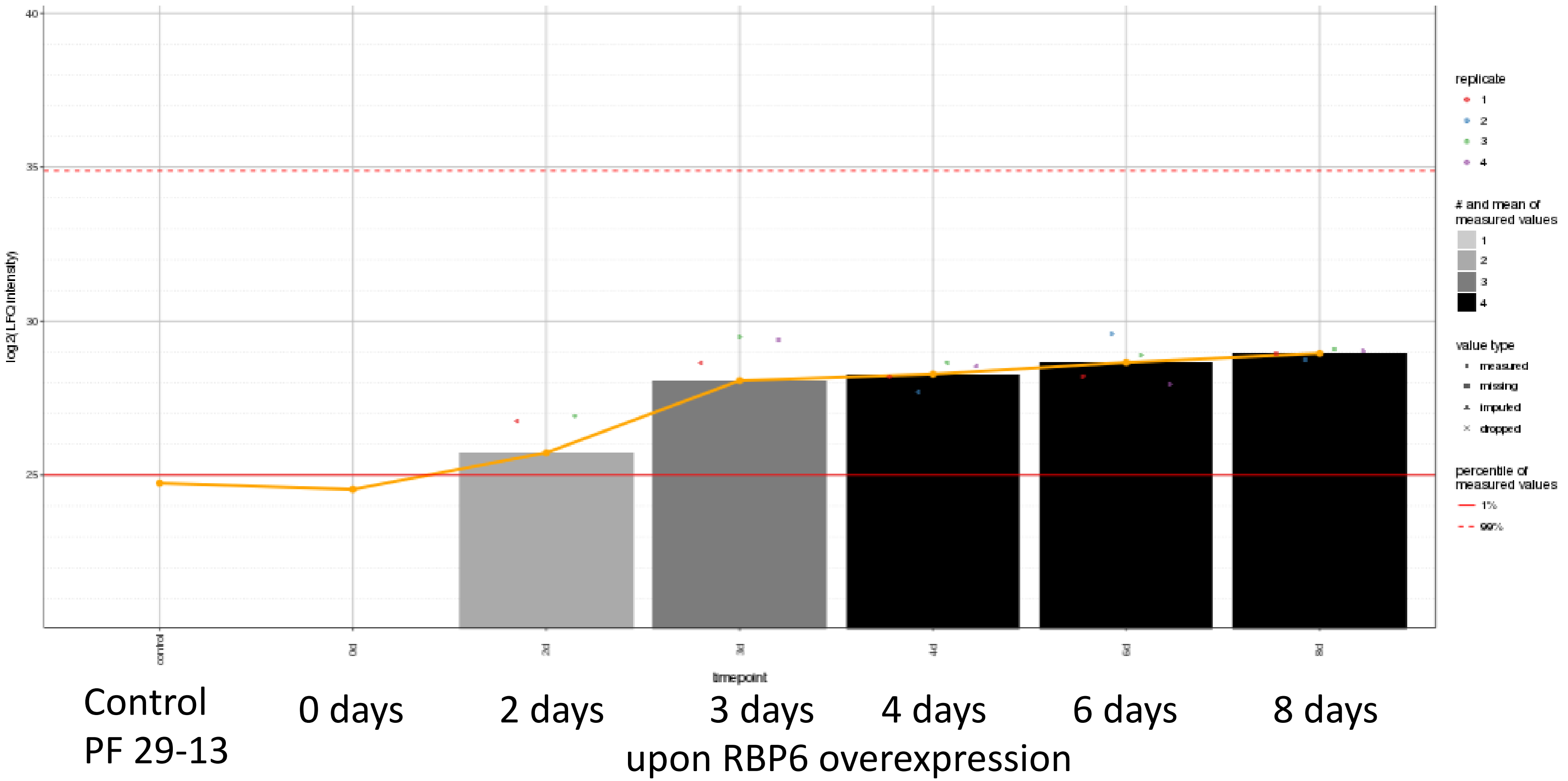

Quantification of MISP peptides (all isoforms identified as a single group) (log_2 LFQ intensity) in proteomics dataset comparing control *T. b. brucei* 29:13 procyclic cultured forms (Control) with cells at 0, 2, 3, 4, 6, and 8 post-induction of RBP6 overexpression. Coloured dots indicate replicate values according to legend; connected bars indicate mean values.

Brucei Alanine-Rich Proteins (BARP)

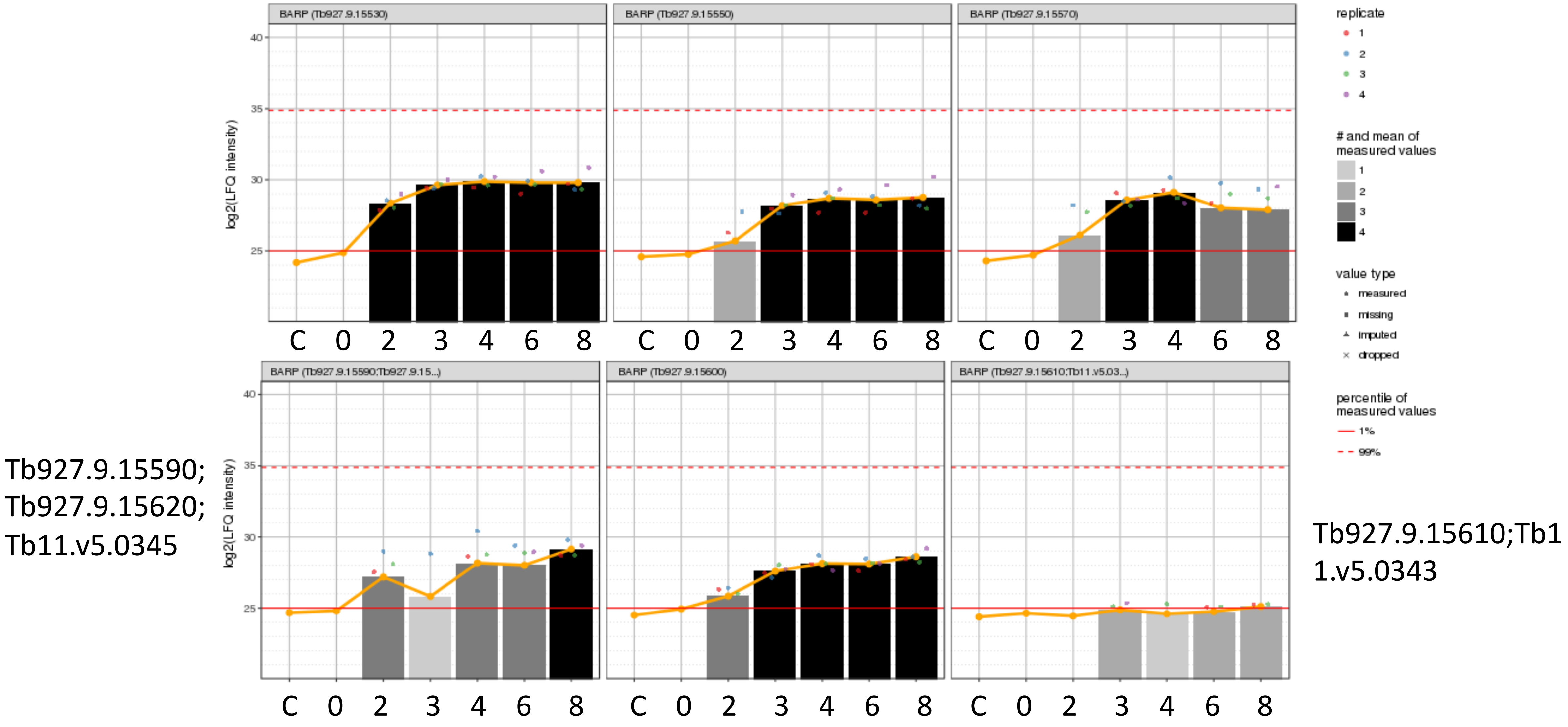

Quantification of BARP peptides (grouped isoforms in individual graphs; log_2 LFQ intensity) in proteomics dataset comparing control *T. b. brucei* 29:13 procyclic cultured forms (C) with cells at 0, 2, 3, 4, 6, and 8 post-induction of RBP6 overexpression. Coloured dots indicate replicate values according to legend; connected bars indicate mean values.

Metacyclic VSG (mVSG) combined

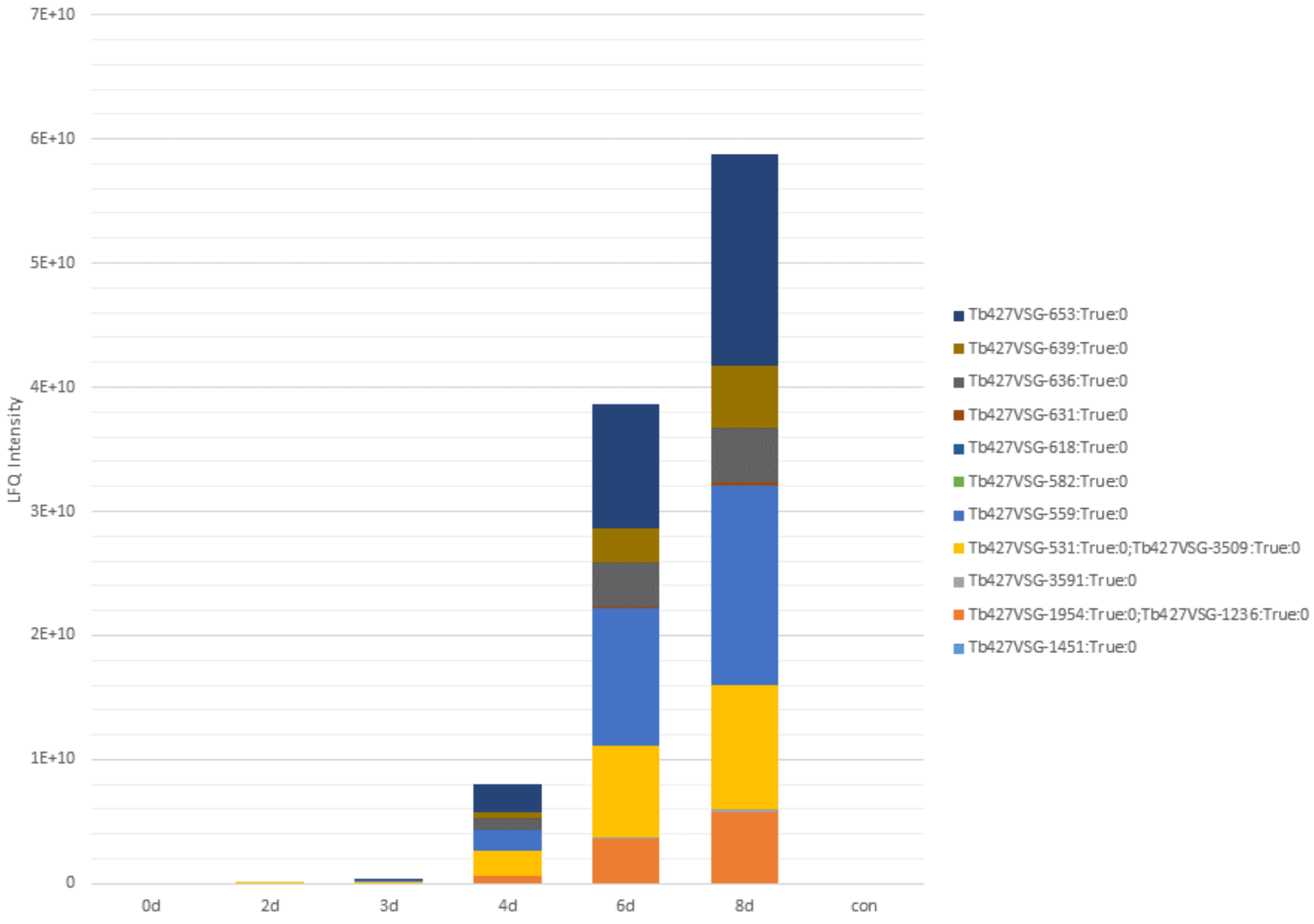

Quantification of mVSG peptides (coloured according to figure legend; LFQ intensity) in proteomics dataset comparing control *T. b. brucei* 29:13 procyclic cultured forms (Con) with cells at 0, 2, 3, 4, 6, and 8 post-induction of RBP6 overexpression.

Alpha tubulin controls

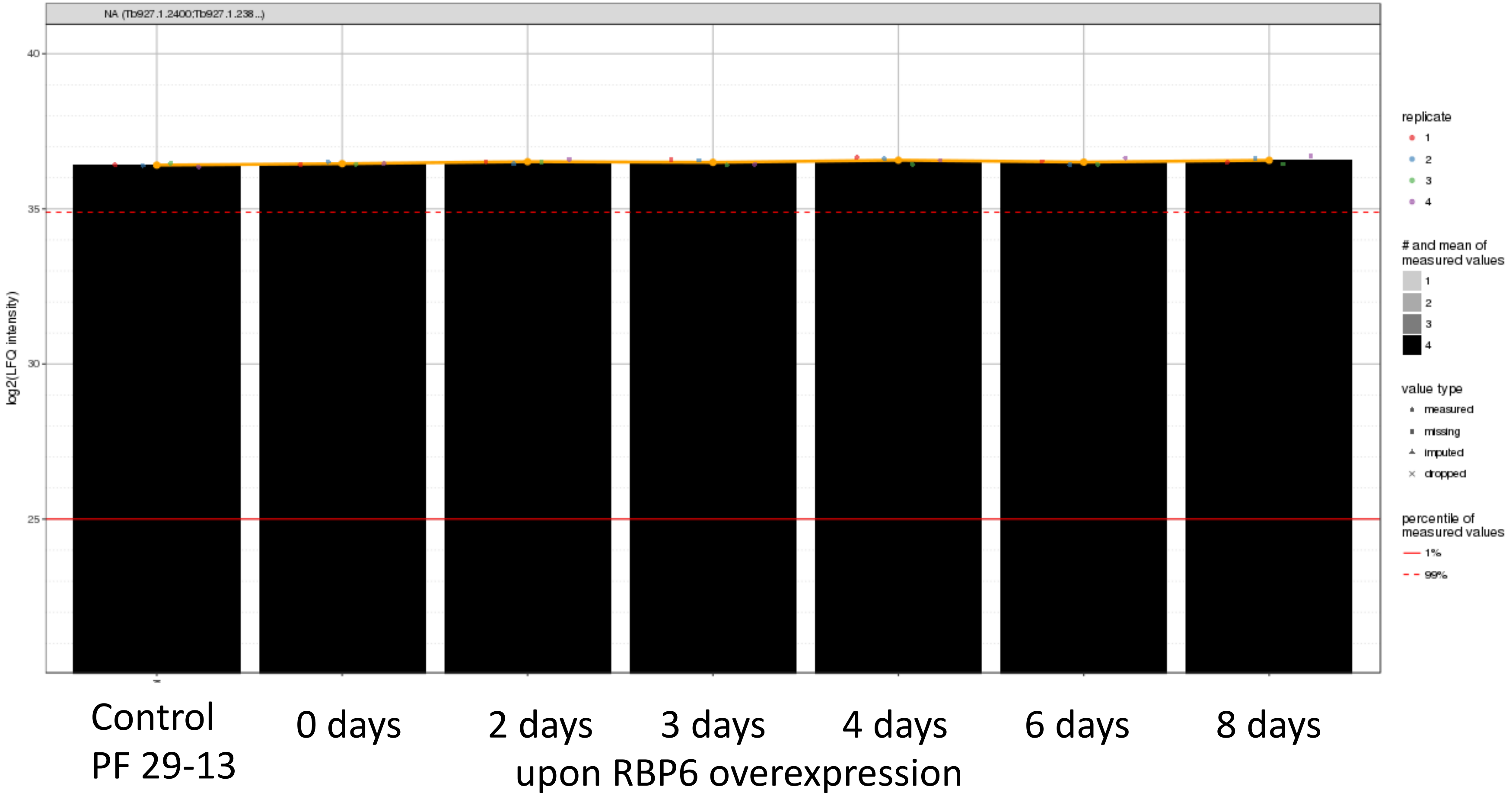

Quantification of alpha tubulin peptides (log_2 LFQ intensity) as house keeping/loading control in proteomics dataset comparing control *T. b. brucei* 29:13 procyclic cultured forms (Control) with cells at 0, 2, 3, 4, 6, and 8 post-induction of RBP6 overexpression. Coloured dots indicate replicate values according to legend; connected bars indicate mean values.

Culture composition during overexpression of RBP6

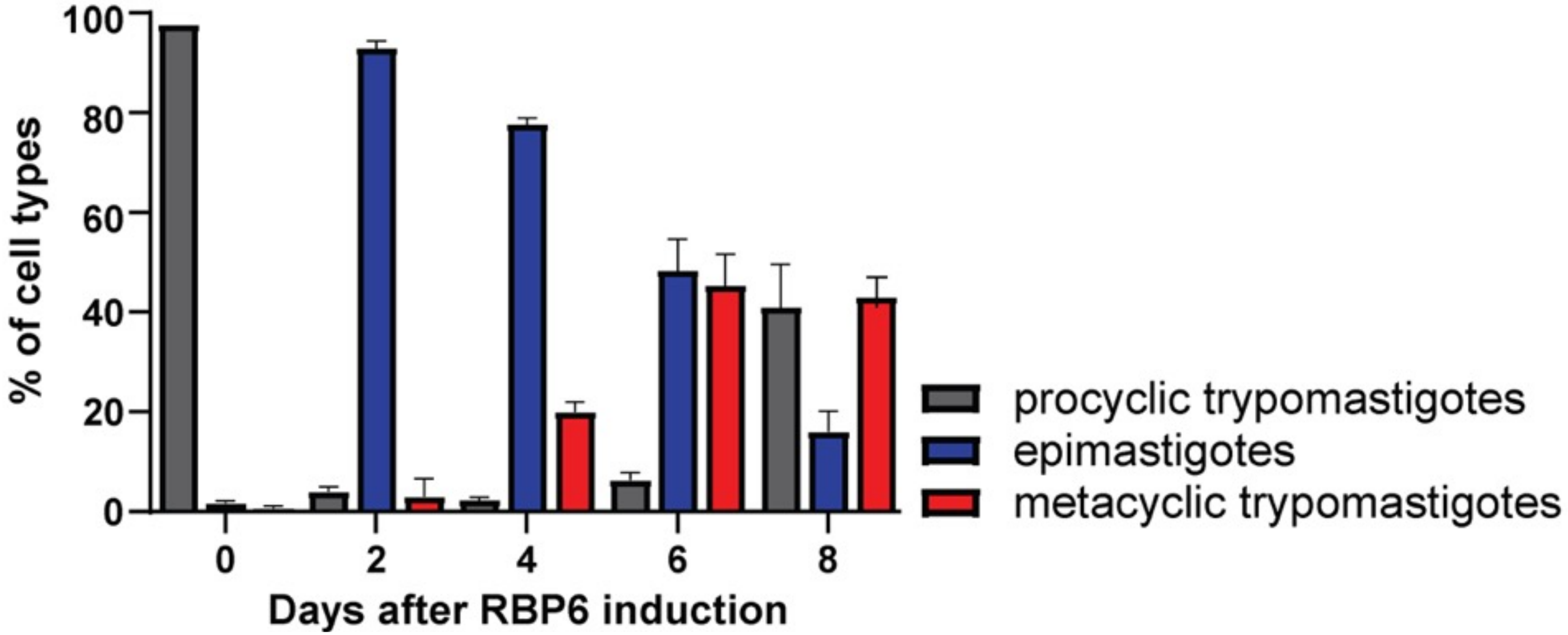

Adapted from *Doleželová et al. 2020 PLoS Biology*; Figure 1E. Percentage of procyclic trypomastigotes (gray), epimastigotes (blue), and metacyclic trypomastigotes (red) in the *T. b. brucei* 29:13 culture at 0, 2, 4, 6, and 8 days post overexpression of RBP6. Bars indicate mean values; error bars indicate standard deviation.
