# Supplementary material for: The Trypanosoma brucei MISP family of invariant proteins is co-expressed with BARP as triple helical bundle structures on the surface of salivary gland forms, but is dispensable for parasite development within the tsetse vector
Source: PLoS Pathog. 2023 Mar 30;19(3):e1011269. doi: 10.1371/journal.ppat.1011269 (PMC10089363; doi:10.1371/journal.ppat.1011269)
Supplement: S4 Table — (DOCX) [file ppat.1011269.s030.docx]

| **Protein ID ^(a)^** | **Annotation** | **Peptides** | **Spectra** |
| --- | --- | --- | --- |
| Tb927.1.2390 | Beta tubulin | 8 | 12 |
| Tb927.11.11330 | Heat shock protein 70 | 4 | 4 |
| Tb927.10.5620* | Fructose bi-phosphate aldolase | 2 | 2 |
| Tb927.9.6210* | Arginine kinase | 2 | 2 |
| Tb927.10.4560 | Elongation factor 2 | 2 | 2 |
| Tb927.10.10280* | Microtubule-associated protein | 2 | 2 |
| Tb927.10.10460* | Histone 2B, putative | 2 | 2 |
| Tb927.11.3510 | Peptidylpropyl isomerase | 1 | 1 |
| Tb927.3.4850 | Enoyl-CoA hydratase, mitochondrial, putative | 1 | 1 |
| Tb927.11.2470 | Metallo-peptidase, Clan MF, Family M17 | 1 | 1 |
| Tb927.4.2740 | p-25 alpha, putative | 1 | 1 |
| Tb927.10.10890* | Heat shock protein, putative | 1 | 1 |
| Tb927.2.2440* | Proteasome regulatory non-ATPase subunit 6 | 1 | 1 |
| Tb927.10.6510* | Chaperonin HSP60, mitochondrial | 1 | 1 |
| Tb927.4.1340 | Cleavage and polyadenylation specific factor subunit, putative | 1 | 1 |
| Tb927.8.6370 | Cytoskeleton associated protein, putative | 1 | 1 |
| Tb927.3.5530 | Tb-292 membrane associated protein | 1 | 1 |

**S4 Table. Intracellular *T. b. brucei* proteins identified by nLC-MS/MS in infected tsetse saliva.** All identified proteins and peptides have >95% confidence and were manually curated. Accession code (Protein ID), protein description (Annotation), number of identified unique peptides (Peptides), and number of unique spectra (Spectra) are indicated.

^(1)^: Accession code of coding gene in strain TREU927, TriTrypDB.

 *: Representative ID of multiple homologs sharing the detected peptide.
