# Supplementary material for: The Trypanosoma brucei MISP family of invariant proteins is co-expressed with BARP as triple helical bundle structures on the surface of salivary gland forms, but is dispensable for parasite development within the tsetse vector
Source: PLoS Pathog. 2023 Mar 30;19(3):e1011269. doi: 10.1371/journal.ppat.1011269 (PMC10089363; doi:10.1371/journal.ppat.1011269)
Supplement: S5 Table — (DOCX) [file ppat.1011269.s031.docx]

**S5 Table. *In silico* predictions based on MISP amino acid sequences.** Accession code (Protein ID), trypanosome species having the coding gene (Species), MISP sub-family (Sub-family), predicted signal peptide (SP), predicted GPI anchor peptide (GPI), number of predicted *N-*glycosylation sites (*N-*glycan) and number of C-terminus 26 residues motifs (C- mot.) are indicated.

| **Protein ID ^(a)^** | **Species** | **Sub-family** | **SP ^(b)^** | **GPI ^(c)^** | ***N-*gly** | **CTR ^(d)^** |
| --- | --- | --- | --- | --- | --- | --- |
| Tb927.7.360 | *T. b. brucei / rhodesiense* | MISP-A | 1-17 | Yes | 1 | 3 |
| Tb927.7.380 | *T. b. brucei / rhodesiense* | MISP-A | 1-17 | Yes | 1 | 2 |
| Tb927.7.400 | *T. b. brucei / rhodesiense* | MISP-B | 1-24 | Yes | 1-2 | 0 |
| Tb927.7.420 | *T. b. brucei / rhodesiense* | MISP-B | 1-24 | Yes | 0 | 0 |
| Tb927.7.440 | *T. b. brucei / rhodesiense* | MISP-A | 1-17 | Yes | 1 | 1 |
| Tbg972.7.270 | *T. b. gambiense* | MISP-B | 1-17/24 | Yes | 1-2 | 0 |
| Tbg972.7.290 | *T. b. gambiense* | MISP-A | 1-17/24 | Yes | 1 | 1 |
| TevSTIB805.7.300 | *T. evansi* | MISP-A | 1-17 | Yes | 0 | 3 |
| TevSTIB805.7.320 | *T. evansi* | MISP-A | 1-17 | Yes | 1 | 2 |
| TevSTIB805.7.380 | *T. evansi* | MISP-A | 1-17 | Yes | 1 | 1 |
| TcIL3000.0.02370 | *T. congolense* | - | 1-22 | Yes | 6-7 | - |

^(a)^: Accession code of coding genes in TriTrypDB.

^(b)^: Starting-ending residues. Peptide cut after ending residue.

^(c)^: All GPI anchor peptides are formed by the last 26 residues of the protein.

^(d)^: 26 residues motives described in Figure 2-B.
