# Supplementary material for: The Trypanosoma brucei MISP family of invariant proteins is co-expressed with BARP as triple helical bundle structures on the surface of salivary gland forms, but is dispensable for parasite development within the tsetse vector
Source: PLoS Pathog. 2023 Mar 30;19(3):e1011269. doi: 10.1371/journal.ppat.1011269 (PMC10089363; doi:10.1371/journal.ppat.1011269)
Supplement: S6 Table — (DOCX) [file ppat.1011269.s032.docx]

**S6 Table. Summary of *T. brucei* VSG species found in either cultured or fly-derived metacyclics**

| **Source ^(a,b)^** | **RNA (RNA-seq) ^(c)^** | **Protein (MS/MS) ^(c)^** | **Reference** |
| --- | --- | --- | --- |
| *Tbb*-infected tsetse salivary glands (RUMP 503) | Tb927.5.3990 (Q57Z50_TRYB2) | - | Telleria et al. 2014 |
| *Tbb*-infected tsetse salivary glands (RUMP 503) | **VSG ILTat 1.22 (VSI2_TRYBB)** | - | Savage et al. 2016 |
|  | **VSG ILTat 1.61 (O97352_9TRYP)** |  |  |
|  | **VSG ILTat 1.63 (Q8MPG1_9TRYP)** |  |  |
|  | **VSG ILTat 1.64 (Q8MPG0_9TRYP)** |  |  |
| Saliva from *Tbb*-infected tsetse (EATRO 1125 AnTaR 1) | - | **mVAT5 (Q26842_9TRYP)** | Kariithi et al. 2016 |
|  |  | VSG 1228 (A0A1J0R6Q7_9TRYP) / VSG 1255 (A0A1J0R6U9_9TRYP) |  |
|  |  | VSG 725 (M4SU87_9TRYP) |  |
| Cultured *T. brucei* MCF (Lister 427 29:13 *rbp6* overexpressor) | **VSG 397 (A0A1J0R4A4_9TRYP)** | **VSG 397 (A0A1J0R4A4_9TRYP)** | Christiano et al. 2017 |
|  | **VSG 531 (M4SYA9_9TRYP)** | **VSG 531 (M4SYA9_9TRYP)** |  |
|  | **VSG 639 (M4TDP9_9TRYP)** | **VSG 639 (M4TDP9_9TRYP)** |  |
|  | **VSG 653 (M4SYN2_9TRYP)** | **VSG 653 (M4SYN2_9TRYP)** |  |
|  | **VSG 1954 (M4T0T6_9TRYP)** | **VSG 1954 (M4T0T6_9TRYP)** |  |
|  | Tb927.5.4690 / Tb927.9.1050 / Tb927.11.18330 ^(d)^ | Tb927.5.291b (B2ZWB3_TRYB2) |  |
|  | Tb927.1.5300 ^(e)^ | Tb927.9.7380 / Tb927.1.5060 ^(f)^ |  |
| Saliva from *Tbb*-infected tsetse (TSW-196) | - | **mVAT4 (O76421_TRYBR)** | This work |
|  |  | VSG 221(VSM2_TRYBB) |  |
|  |  | VSG 4959 (A0A1J0RB71_9TRYP) |  |
|  |  | VSG 3088 (M4SWM0_9TRYP) |  |
|  |  | VSG 408/646/769/3613/  474/1142/4207/4707  (M4SWZ7_9TRYP) |  |

**Metacyclic VSGs in bold font**

^(a)^ *In vivo* samples isolated from infected *G. m. morsitans* flies

^(b)^ *T. brucei* strain in parentheses

^(c)^ VSG species; TritrypDB gene accession code; UniProt protein accession code

^(d)^ Genes with higher differential expression (MCF compared to PCF) than canonical mVSGs (>1100 fold)

^(e)^ There are 11 more VSG genes with differential expression MCF/PCF within the canonical mVSG range (250 to 730 fold)

^(f)^ Also upregulated in RNA-seq data
