# Supplementary material for: The Trypanosoma brucei MISP family of invariant proteins is co-expressed with BARP as triple helical bundle structures on the surface of salivary gland forms, but is dispensable for parasite development within the tsetse vector
Source: PLoS Pathog. 2023 Mar 30;19(3):e1011269. doi: 10.1371/journal.ppat.1011269 (PMC10089363; doi:10.1371/journal.ppat.1011269)
Supplement: S7 Table — (DOCX) [file ppat.1011269.s033.docx]

**S7 Table. Data collection and refinement statistics of *Tb*MISP360 crystal structure**

|  | *Tb*427.07.360 |  |
| --- | --- | --- |
| A. Data collection |  |  |
| Synchrotron source | CLS |  |
| Space group | *P*2_1_2_1_2_1_ |  |
| *a*, *b*, *c* (Å) | 24.81, 79.50, 108.17 |  |
| *α* = *β* = *γ* (°) | 90.00 |  |
| Wavelength (Å) | 0.9795 |  |
| Temperature (K) | 100 |  |
| Resolution range (Å) | 44.72-1.82 (1.92–1.82) |  |
| Measured reflections | 136043 |  |
| Unique reflections | 19605 (2715) |  |
| Redundancy | 6.9 (6.4) |  |
| Completeness (%) | 98.2 (94.9) |  |
| *I/σ(I)* | 20.8 (9.3) |  |
| *R*_merge_^a^ (%) | 6.0 (15.9) |  |
|  |  |  |
| B. Refinement Statistics |  |  |
| Resolution (Å) | 37.31-1.82 (1.88–1.82) |  |
| *R*_cryst_^b^ / *R*_free_^c^ (%) | 16.94(19.24)/20.28(29.20) |  |
| No. of atoms |  |  |
| Overall | 3,347 |  |
| Protein | 3,074 |  |
| Solvent/Heterogen atoms | 273 |  |
| Mean temperature factor (Å^2^) |  |  |
| Overall | 12.3 |  |
| Protein | 11.3 |  |
| Solvent/Heterogen atoms | 18.0 |  |
| r.m.s. deviation from ideality |  |  |
| Bond lengths (Å) | 0.010 |  |
| Bond angles (°) | 1.14 |  |
| Ramachandran statistics |  |  |
| Most favored (%) | 99.5 |  |
| Allowed (%) | 6.6 |  |
| Generously allowed (%) | 0.0 |  |
| Disallowed (%) | 0.0 |  |
| Values in parentheses are for the highest resolution shell | | |
| ^a^ *R*_merge_= ∑*_hkl_* ∑*_i_* \|I*_hkl,i_* - [I*_hkl_*]\| / ∑*_hkl_* ∑*_i_* I*_hkl,i_*, where [I*_hkl_*] is the is the average  of symmetry related observations of a unique reflection | | |
| ^b^ *R*_cryst_=∑\|F_obs_-F_calc_\|/∑F_fobs_, where F_obs_ and F_calc_ are the observed and the calculated  structure factors, respectively. | | |
| ^c^ *R*_free_ is R using 5% of reflections randomly chosen and omitted from refinement | | |
| ^d^ Ramachandran statistics were determined using PROCHECK | | |
