# Supplementary material for: The Trypanosoma brucei MISP family of invariant proteins is co-expressed with BARP as triple helical bundle structures on the surface of salivary gland forms, but is dispensable for parasite development within the tsetse vector
Source: PLoS Pathog. 2023 Mar 30;19(3):e1011269. doi: 10.1371/journal.ppat.1011269 (PMC10089363; doi:10.1371/journal.ppat.1011269)
Supplement: Attachment — Submitted filename: Responses to Editor & reviewers.docx [file ppat.1011269.s034.docx]

Dear Editor,

We would like to thank you and the reviewers for the positive feedback on our manuscript "The *Trypanosoma brucei* MISP family of invariant proteins are co-expressed with BARP as triple helical bundle structures on the surface of salivary gland forms, but is dispensable for parasite development within the tsetse vector".

**Responses to the general comments and requests** **made by the editor.**

Taking on board several of the reviewer's comments, we have focused on performing the main experiments to corroborate the specificity of our anti-MISP antibody. Understanding the function of MISP in the biology of *T. brucei* in the fly is beyond our capacity as transmission experiments and identification of a potential salivary gland ligand are lengthy experiments demanding time and resources we no longer have. In addition, I’m currently moving my research group to Notre Dame University (USA) and so I no longer have staff and access to a tsetse insectary (the one at LSTM has been significantly reduced in size).

As requested, we infected a new batch of teneral flies with either wild type AnTat 1.1, or MISP null mutant, or dox-induced MISP null mutant (addback (AB+)) trypanosomes and analyzed the staining of free swimming metacyclic trypanosomes from each group by fluorescence microscopy. Although the number of infected salivary glands in all groups was rather low (~2% SG infection in all groups), the number of metacyclics obtained in saliva was enough to perform new IF experiments using anti-MISP or anti-BARP antibodies (same ones described in the previous submission). As predicted, our polyclonal anti-MISP antibody did not recognize metacyclics extracted from flies infected with MISP null mutant cells, whereas it bound to the surface of metacyclics from WT and add back cells re-expressing MISP360 (these results have been incorporated in new Fig 5C and S20 Fig). Moreover, i) the specificity of anti-MISP and anti-BARP antibodies was further corroborated by western blotting (new S23 Fig), and ii) the anti-MISP polyclonal antibody recognizes fly-derived metacyclics from TSW196, another fly-transmissible strain (Paindavoine, P. et al. 1986 Parasitology; S15 Fig). These new results, together with the results that were included in our previous submission, and those reported by other labs using single-cell transcriptomics of fly-derived (Hutchinson S. et al. 2021 PLOS Path.) or transcriptomics and proteomics analyses of in vitro-differentiated metacyclics (Doleželová E. et al. 2020 PLOS Biol. and S3 File), support our claim that MISP is mainly expressed in *T. b. brucei* metacyclics.

1. ***All of the reviewers agree that this paper represents a large amount of high-quality data. The reviewers disagree, however, about whether the results are sufficient for publication in the journal, because you have not actually found any function for these proteins. The decision is based on the premise that - as stated by the more positive reviews - sometimes, negative results are indeed important.***

Thank you. We appreciate the acknowledgment for publishing negative results despite our efforts to determine the function of MISP proteins. We think our paper merits publication in PLoS Pathogens because we provide novel data on the crystal structure of the MISP N-terminal domain (including comparison with the high confidence BARP model), determine the correct surface expression, and present the CRISPRCas9 KO and RNAi evidence proving MISP is not essential for developing mature trypanosome infections in the tsetse salivary glands.

***In addition, the effects on salivary gland infection might be interesting (although of course, the lack of add-back restoration is a problem that has to be highlighted)*.**

Indeed, the reviewer has identified a frustrating problem, although not so uncommon in trypanosomes. It is difficult to predict if the lack of full restoration in the add back cells is a consequence of low levels of MISP expression, the expression of an “incorrect” MISP isoform, or to the fact that a combined set of MISP isoforms is needed to match the WT infection phenotype in salivary glands. To test these possibilities, we are limited by the prevalence of tsetse salivary gland infections (usually very low (10-15%)). Furthermore, it takes at least 4 weeks post-infection before metacyclics in spat saliva from infected flies can be obtained (common characteristics among all the transmissible *T. brucei* strains used in our group in recent years).

2. ***One major issue is clearly the fact that these proteins were previously identified in the Vigneron paper. It is very important that you describe the relevant results from that paper clearly and with sufficient detail in the Introduction, both for the sake of transparency and so that readers can bear in mind the discrepancies as they read your results section.***

We apologize for the lack of reference of the Vigneron et al work, especially in the Introduction. This has been now amended throughout the text.

3. ***Since the difference between your results and those of Vigneron et al. could well be caused by the difference between the antibodies used, one more immunofluorescence experiment is essential. This is to use your polyclonal antibody to stain the null mutant, comparing with the wild-type, in order to rule out anti-MISP cross-reactivities (for example, with VSG). (I actually find it odd that you didn't do this already, since you did it for the RNAi.) Only then would the re-naming of these proteins as "MISP" be justified.***

As suggested (and explained previously), we performed these experiments and the results are now included in (Fig 5C, S20 Fig), in addition to further corroborating the specificity of our anti-MISP antibody by western blotting (new S23 Fig).

4. ***This, together with at least parts of S16, should be part of the main text***

Several panels of S16 are now in Fig 5, which highlights the characterization of the MISP null cells over the RNAi experiments (now in S19 Fig).

5. ***The Figure could also include S8.*** This figure remained as supplemental material as it refers to the relative expression of MISP and BARP in wild type cells only; we did not look at the relative expression of BARP in the RNAi or MISP null cells.
6. ***The extra experiment could also sort out whether the re-expression was insufficient, lines 517-519.*** The extra experiment shows that MISP expression in add back cells appear equivalent to that of WT cells. Unfortunately, due to the low number of metacyclic cells obtained and the priority given for IFA, we were unable to quantify the levels of *misp* transcript and/or protein by fluorescence intensity quantification.
7. ***And at the same time you can get results concerning the epimastigote/metacyclic balance in the null mutant. (If there is a difference it might be worth checking infectivity to mice, or at least growth in vitro as bloodstream forms.)*.** Unfortunately, we have not found a way to determine the exact ratio of epimastigote, pre-metacyclic and metacyclic forms in an infected salivary gland as most (>95%) of the epimastigotes remain attached to the epithelium after dissection. Likewise, most trypanosome forms in infected saliva are free-swimming metacyclics, but occasionally we are able to detect some free epimastigotes and premetacyclics (as those shown in Fig 2B and C). In addition, as explained above, the unexpectedly low percentage of salivary gland infections in all groups (<2%) did not make possible such quantification.
8. ***Assuming the IFA result is as you hope, you should also do the co-staining with anti-BARP and anti-MISP. It is essential to define precisely how you distinguish between procyclics, epimastigotes and metacyclics, and to state how the images were blinded before analysis.***

Unfortunately, all the polyclonal antibodies available for this work were raised in rabbit and therefore co-staining was not possible.

Regarding identifying parasite salivary gland stages, in addition to having different morphologies, there is a striking distinction between epimastigotes and metacyclics, which is based on i) total parasite length; ii) the location of the kDNA in relation to the nucleus (i.e., anterior end in epimastigotes *vs* posterior end in metacyclics); iii) only metacyclics are recognised by anti-CRD antibodies (Zamze S et al 1988 Eur. J. Biochem., and Roterau B et al 2012 Development); iv) premetacyclic trypanosomes were identified using the descriptions reported by Roterau B et al 2012, which was based on the proximity between the nucleus and kDNA, parasite shape and the lack of recognition by anti-CRD antibodies. We have now included a series of measurements that further complement our observations (S11 Fig).

9. ***Of course if you DO see staining of the null mutant, the whole interpretation would need to be re-assessed.***

As described previously, the additional infection experiment confirmed the null mutant did not stain with anti-MISP polyclonal antibody.

10. ***As noted by one of the reviewers, SDs and significance values with only two replicates are invalid since there is no way to know whether the distribution is normal. Please show the replicates instead and remove the SD and claims of significance. In fact, you should get more data anyway from the essential IFA with the null mutant described above, so you will actually have biological replicates: technical replicates are anyway insufficient. (Note that two technical +two technical doesn't make four replicates!)***

As requested, we have now shown in Fig 5B the salivary gland infection percentages per biological replicate, noting the sample size of each replicate, and removing the mean values and SDs. Instead, we indicate the binomial standard error of sample proportion of each replicate as error bars, and we do not indicate significance. We analyzed the data using logistic regression which did not provide evidence of significant differences between groups.

Unfortunately, we couldn’t include in this graph the results of the latest experiment used for IF analysis as the number of infected salivary glands (and consequently the number isolated metacyclics) by all cell lines was extremely low (1-2 flies with infected salivary glands per group).

***Obviously, all the data needs to be in an appropriate databases - PRIDE or similar for the MS***

All mass spec data has now been deposited in PRIDE (dataset identifier PXD039684). Data can be found using Username: [reviewer_pxd039684@ebi.ac.uk](mailto:reviewer_pxd039684@ebi.ac.uk) / Password: 0hBp6EV5

11. ***and PLoS is now asking for all the full gel images with markers (e.g. S11B, S15 C, D, E and F).*** Full scans of SDS-PAGE images can be found in new S16 Fig.
12. ***and all data tables used for Figures, to be in a database such as Figshare as well. I suggest you make sure everything is uploaded before resubmission, on order to minimise later hassle.***

All data will be included in Figshare as requested, but unfortunately, we did not get the accession number on time before our 27 January submission deadline. We will make sure the Figshare accession number gets included in the final version.

13. ***Once you have done the vital control with the null mutant, please also make sure you have annotated the relevant genes appropriately in TritrypDB.***

We have updated TritrypDB to include MISP as new aliases for Tb927.7.360/380/400/420/440, and linked to the crystal structure deposited in PDB.

**Responses to the reviewers’ comments and questions.**

**Reviewer #1**: ***The manuscript of Casas-Sanchez et al. provides an interesting microbial cell biological and structural characterization of the MISP family of proteins. Significant work goes into identifying the MISP proteins, comparing them, and characterizing them by structural biology. Despite these efforts, the role of the MISP proteins remains as enigmatic at the beginning of the work as by the end of the manuscript. The authors rule out many functions as well as practical applications of the MISP (e.g, use in vaccines) and provide a very solid foundation for further work to understand the role of the proteins in the trypanosome. The experimental work appears very solid (proteomics with MS, crystallography, microbial cell biology, etc). Some of the modeling (e.g, CTD length and conclusions about the MISPs extending beyond the VSGs, possible binding pocket at the top of the molecule, etc), are interesting hypotheses, but not strongly established.***

We thank this reviewer for the positive comments and understanding the value of negative data to rule out potential functions.

**Reviewer #2**: ***The manuscript describes the identification of MISP proteins in infected tsetse saliva and then goes on to characterise their expression, structure and phenotype of a null mutant. Immunization of mice with MISP did not provide protection against infection by tsetse bite. The mass spec identification of trypanosome proteins in tsetse saliva is fine and probably a technological achievement. The description of the gene family and the description of differences between members is clear.***

***MISP and BARP mRNA levels were compared by ‘semi-quantitative’ RT-PCR and showed that MISP mRNA was present at highest amounts in the salivary extracts but was also presents in the proventriculus. This section would be improved by being absolutely clear it is mRNA expression that is being measured and avoiding phrases like ‘Expression of misp genes ..’ (line 256).***

Thank you for pointing this out. The mistake has been corrected by replacing, where relevant, “genes” for "transcripts” in lines 250-269 of section ‘MISP are expressed on the surface of *T. b. brucei* salivary gland stages’.

***A second item that could be clarified is whether the apparent higher abundance of the mRNA encoding the shorter MISPs is due to a shorter amplicon or whether all the amplicons were similar length?***
The apparent higher abundance of the shorter MISP mRNAs is mainly due to the amplicon being generated from 2 genes with identical sequences (Tb927.7.400 and Tb927.7.420) instead of 1 gene as for the rest of MISP (Tb927.7.360, Tb927.7.380, Tb927.7.440). However, we cannot rule out the possibility that the efficiency of amplification of a shorter amplicon may be higher in comparison to the slightly longer ones. Hence, we tested our method in a cell line overexpressing only MISP360 (the longest) under tetracycline induction (S4 Fig), and we successfully detected an increase in MISP360 mRNA only when the transgene was induced, hence reassuring that real changes in mRNA abundance of any MISP paralogs can be detected.

***The next experiment looks at MISP and BARP protein expression in different developmental stages using immunofluorescence. No MISP protein was detected in cells from the proventriculus. In the salivary glands, MISP and BARP protein was detected in more than one developmental form with MISP being higher in metacyclics than epimastigotes and BARP the other way round. As mentioned in the discussion, the expression pattern of MISP is not in agreement with a previous report from another lab (***[***https://doi.org/10.1073/pnas.1914423117***](https://protect-eu.mimecast.com/s/ABw1C7pm0iz97PycBFMPD?domain=doi.org)***). The availability of the MISP null mutant (see below) should be exploited to demonstrate the specificity of the MISP antiserum and resolve the discrepancy.***
We can confirm the MISP polyclonal antiserum does not recognise MISP null mutants. The explanation of the whole experiment is described on page 1 of this document, and the results are shown in Fig 5C and S20 Fig.

***The structure of one MISP was determined by X-ray crystallography and this showed that the protein adopts a 3-helical bundle structure found in a series of trypanosome surface proteins. This in turn was used to produce a high confidence model of BARP. The work on MISP is fine although the speculation of the pocket being a ligand binding site is just speculation.***

We agree with the reviewer and have lowered the tone about the MISP pocket being a ligand binding site in the Discussion.

***The next experiment tested whether pre-immunization with MISP would protect mice against an infected tsetse fly challenge. The experiment is fine and immunization did not protect.***

***The final set of experiments showed that both an MISP RNAi knock down and a MISP null mutant were able to progress through the salivary glands normally. The null mutant makes the RNAi experiment redundant.***
Although both approaches may look redundant, they are instead complementary. However, in the revised version, we have highlighted the null mutant data by moving the figures to the main paper (Fig 5) and the RNAi experiments have been transferred to the supplementary section (S19 Fig).

*The manuscript could do with some thorough editing, it comes across as disjointed.*

As requested, we have revised and edited the manuscript for consistency and clarity.

**Reviewer #3**: ***While a previous study both mentioned and conducted a few experiments regarding MISP proteins, the experiments conducted were highly different and included a much more rigorous and in depth focus on this particular family of proteins. While the exact timing of expression differs between the two, these can be explained when looking deeper into the differences of the techniques. Aside from the timing of expression inside the salivary gland, investigation into the proteins in the tsetse saliva, the release of surface protein coat into tsetse saliva, the investigation into the knock-out of MISP proteins on fly infections, and the elucidation of the crystal structure of MISP proteins are all novel findings.

The methodology performed in this study was extremely rigorous. The extent of different techniques which were performed showed a truly in-depth and multi-faceted analysis of a surface coat protein family on a mammalian infective stage of Trypanosoma brucei.***
Thank you for understanding the breadth of our experimental approach and the new data we present in this manuscript

***Main claims of the paper:
This paper aimed to uncover proteins found in the tsetse fly vector saliva which contribute to transmission of Trypanosoma brucei. The data set which Casas-Sanchez et al. produced was no small feat, as the fly dissections alone for this incredible number of flies (3000!) is quite labor and time intensive. Aside from vector proteins, they found an increase in bacterial symbiont proteins as well as proteins from the trypanosomes themselves, including those found on the surface coat of the parasite. The authors then did a deep dive, implementing a wide array of methods to uncover the mechanism of one of these protein families. They found that the previously poorly described surface proteins, termed Metacyclic Invariant Surface Proteins (MISPs), are found on the surface of epimastigote, pre-metacyclic, and metacyclic cells in increasing amounts as they differentiate in the fly salivary gland. Further, they were able to crystalize and analyze the MISP proteins, showing they have a similar structure not only to BARP on T. brucei but also other uncharacterized coat proteins in related species. Less visually displayed in their paper but no less important was the deletion and add back of all of the MISP proteins from the parasite to check its role in infecting tsetse flies as well as probing these surface proteins as a potential vaccine target (though using a MISP protein as a vaccine candidates had been done previously in Aksoy et al.the end results were confirmed). Though both experiments resulted in negative data, the experiments are no less important and are still telling in that we now know the protein family is not essential to protein colonization of the salivary gland and are not suitable vaccine targets, at least using GLA-AF.

It is important to note that a previous study, Vigneron et al. PNAS 2020, also looked at one MISP protein (termed SGE-1), albeit with different methods, which they had found during an RNA-seq experiment. Vigeron et al. found that the protein is expressed during a different life cycle stage. They showed that a MISP (or SGE-1) protein is expressed only by epimastigotes in the salivary glands while Casa-Sanchez et al. show that MISP expression begins in epimastigotes and then expression increases as they become metacyclics, while BARP expression is the exact opposite.***

***Casa-Sanchez et al. offer 3 reasons for this divergence in results.
The first reason includes the fundamental differences in any experimental set up. Namely, in strains and conditions used. Vigeron et al. used the RUMP 53 strain and looked at salivary gland infection from 30-33 dpi using only female flies. Casas-Sanchez et al. used the TSW196 strain and looked at salivary gland infection 28-30 dpi using only male flies. As Casa-Sanchez et al. showed that these conserved MISP proteins are non-essential, this may have allowed expression differ between the strains. This, however, would be quite easy to test, especially as Casa-Sanchez et al. use chemicals to increase infection rate, lowering the number of flies they would need to use. We would recommend that they infect flies with a different strain of trypanosome and perform immunofluorescence to check that the expression pattern of MISP matches what they saw with TSW196, rather than only the epimastigote stage as Aksoy et al. claim.***

Thanks for the positive comments. The IF experiments showing the expression of MISP and BARP in infected salivary glands and saliva (Fig 2) were done using AnTat 1.1. We have now included a representative image showing MISP expression in TSW196 metacyclic (S15 Fig), which were not included in the previous submission. The RNAi experiments and generation of MISP null mutants were done in AnTat 1.1, because this strain is genetically tractable (i.e suitable for RNAi and CRISPRCas9 KO) and still retain the capacity to infect salivary glands (a common problem in trypanosome strains that have been genetically modified or passaged in vitro for several years).

***Difference in infection days was another reason offered for the difference and this could also be directly tied to the fly sexes used. The trypanosome field normally uses male flies when looking at infection processes, as female flies are known to be less easily infected with T. brucei. As the difference in infection rate between the sexes is already known (though the mechanisms of which are hypothesized but not fully understood), this could also cause differences in trypanosome development timing. While possible, we find that this reason is unlikely as the different stages of trypanosomes should still have a similar expression pattern whether the fly has been infected for 28 days or 33 days. However, both papers are somewhat unclear about how they identified epimastigotes vs. pre-metacyclic vs. metacyclics. Both Casa-Sanchez et al. and Vigeron et al. do not clarify their method of differentiating between the stages. While it seems obvious that this was based on morphology, kinetoplast – nucleus placement, and attachment, adding a sentence about exactly how the authors defined the stages would help clear this up. This would especially be helpful as Vigeron et al. does not have a pre-metacyclic stage included in their protein results and could be another source of the difference of results.***

We thank the reviewer for pointing this out. In addition to the qualitative criteria of identifying the epimastigote form (i.e. parasite shape, kDNA positioned towards the anterior end, close proximity between kDNA and nucleus, recognition by anti-BARP antibodies, and lack of surface staining by anti-CRD antibodies), we have now included cellular measurements to further validate the identification of the life-cycle stages described in our work, as described in Rotereau B et al. Development 2012. These measurements include total cell length, distance from kDNA to the posterior end, distance from nucleus to posterior end, and distance between nucleus and kDNA (new S11 Fig).

***In terms of fly timing, as Casa-Sanchez et al. have already laid out solid experiments with clear data and hypothesis’, looking further into differences in the individual fly infection days in which the non-essential MISP proteins have different expression would just not be feasible as the experiments would take too long and be too labor intensive when there is a high potential that this was not the cause of the difference.***
We thank the reviewer for acknowledging the technical challenges behind the infection of flies with trypanosomes.

***In my view, the next two explanations given for the differences between the two papers results hold a lot more merit. The reasons being that transcription does not equal translation and that monoclonal antibodies can experience problems binding epitopes which can be blocked by tightly packed proteins, such as the VSG coat. While RNA-sequencing is a very powerful tool to look at gene expression, final protein cannot be confirmed until protein studies are done. Vigeron et al. did follow up looking at the MISP proteins by using immunofluorescence but used monoclonal antibodies which were made against whole protein, with the unknown epitope being anywhere on the protein. As Vigeron et al. only used non-permeabilized cells for the antibody work, it is very possible that the mVSG on the metacyclics blocked binding in metcyclics and not in the epimastigotes which have just started to express MISP. Meanwhile, Casas-Sanchez et al. used polyclonal antibodies, used both permeabilized and non-permeabilized cells, as well as live cells to confirm expression.***

We agree with this interpretation, although our discrepancies with Vigneron et al could still be due to a combination of several factors.

**Reviewer #4:** ***The authors did an analysis of tsetse saliva to identify secreted proteins. They identify a single peptide of Tb927.7.360, which belongs to a gene family of 5 proteins. Despite being 1 peptide, the single MS/MS spectra for this peptide looks very convincing. They follow the expression of these genes in different stages by a modified RT-PCR protocol and IF. They obtained a crystal structure for parts of TbMISP-360. Functionally, the proteins do not protect against T.brucei infection and are not essential for colonization of the SG.***

**Part II – Major Issues: Key Experiments Required for Acceptance**

Reviewer #1: (No Response)

***Reviewer #2: One experiment only: use of the MISP null mutant to demonstrate specificity of MISP antiserum.***

Done and addressed in Fig 5C and S20 Fig as previously described.

***Reviewer #3: none***

***Reviewer #4: The manuscript is well-written and nicely structured. Although a lot of work by the authors, it unfortunately remains descriptive due to a lack of functional understanding. I would consider it a strong manuscript for publication here, if there would be any insight into its function. At the moment, I feel the manuscript is more suited for a more specialized journal.***

We respectfully disagree with the reviewer’s comment as we have shown a substantial amount of novel data (summarised in the paragraph addressed to the editor), and even performed technically challenging experiments like 1) determining MISP’s immunoprotective properties against an infection bite, and 2) addressing the essentiality of MISP (by generating CRISPRCas9 MISP null mutants and RNAi experiments) during the colonization of salivary glands by trypanosomes. As far as we are aware, this is the first report addressing the essentiality of a *T. brucei* protein expressed within the tsetse using CRISPR-Cas9, so the methodology will be a valuable asset for the community.

***To gain insight, omics methods might be of help. The authors tested very specific scenarios of their protein functionality. How about performing transcriptomics/proteomics in the knockout strains to shed light on changes during SG infection or after establishment in the vertebrate skin. Following more targeted, the authors suggest a functionality in skin colonization. This could actually be checked experimentally.***

Thanks for the great suggestions, which we plan to due for a subsequent manuscript. The main reason for our inability to include further large-scale experiments such as the ones suggested is that, as pointed out above, I’m in the process of moving my group to the University of Notre Dame, USA. This means I currently have no postdocs and technicians within my group and, equally important, the LSTM tsetse facility has been significantly reduced as it will be used mainly for teaching purposes. To get significant number of metacyclic trypanosomes from infected flies for omics work, we’d need to infect a significant number of flies and have the human resources to dissect and process the 'omic data.

***For the crystal structure, maybe the authors could integrate folding predictions from alphafold to gain also ideas about the other members of the protein family and to further support their homology fold model of the misp360.***

Although a good suggestion, the focus of the current manuscript is such that we intentionally limited the use of structural modelling of the broader family members. This decision was made, in part, due to ongoing efforts to complete full structural characterization of these proteins including the regions that are poorly modelled even by programs such as alphafold.

***I also wonder whether the authors could further substantiate their claim that especially MISP-A is extending beyond the VSG coat (Figure 3H).***

Unfortunately, I don't think we have enough data to contribute much beyond what has already been explained in the text. However, in S14 Fig, we show evidence of MISP surface exposure (although without knowing which isoform(s) is expressed) by immunostaining with anti-MISP on live AnTat 1.1 metacyclic and epimastigote forms.

***The functional characterizations are unfortunately not leading to a further characterization or even a lead on the function of the proteins.***

Thanks for acknowledging our efforts to understand the function of MISP. This was a frustrating outcome, but believe in the value of publishing negative data.

***However, I wonder why the authors just reintroduced MISP360 and not a cassette with all misp genes as this might have led to a rescue of the albeit small, but significant salivary gland infection rates.***

The reason why we first focused on MISP360 is because this was the isoform we obtained the crystal structure from (partly because at that time it was the easiest to express in *E. coli*). We thought of using the MISP null cells to re-express one or several MISP isoforms at the time, but we ran out of time to create the transgenic lines and test their infection phenotypes in flies.

***The protein is obviously expressed in metacyclics generated by overexpression of RBP6. This could be utilized to study the metacyclics in the in vitro culture system. Do they show differences during the differentiations? These systems are readily accessible for growth, differentiation, transcriptomics and proteomics…***

Indeed, cultured procyclic cells expressing RBP6 tend to differentiate all the way to metacyclics, although the differentiation rate appears to vary among the different cell lines generated by the tryp research community. However, the group of Alena Zíková seems to have perfected the differentiation timing in RBP6 cells and even obtained high quality transcriptomics and proteomics data from different time-points/stages (Doleželová E et al. 2020 PLOS Biol.). In collaboration with Dr Zíková, we have interrogated these datasets and discovered that MISP protein expression peaks when the metacyclic population is at its highest, which matches our IF experiments on MISP localization. These analyses have been included as supplemental data (S3 File) and briefly mentioned within the manuscript. Of relevance is that they generated RBP6 over-expressor cells using the 29:13 clone of the *T. brucei* Lister 427 strain, which is a different strain used in our work and the published work by Vigneron et al. in 2020.

***The authors do a very extensive discussion, but some experiments are rather easy to perform and could contribute to a more solid characterization of the new protein. E.g. the mentioned skin infection behavior or the inclusion of the proteins in EVs.***

Thanks for the interesting suggestions (some of which we already have a plan to do), but again since I’m moving my group to the USA, we won’t the time and access to facilities to perform these experiments within a feasible timeframe.

**Part III – Minor Issues: Editorial and Data Presentation Modifications**

**Reviewer #1**: (No Response)

***Reviewer #2: There has been a previous study on MISPs that gave them a different name and came to different conclusions about the expression pattern. This is addressed above but I feel the authors should make this clearer, probably in the introduction as well as the discussion.***

Already sorted as previously discussed.

***Reviewer #3: Adding another sentence into the methods stating how many flies were used per replicate when collecting the fly saliva of infected vs non-infected tsetse.***
As suggested, this information has been clearly stated in the MM section ‘Tsetse fly infection and dissection’.

***For Figure 1 C. the misp-B arrows are purple rather than dark blue, as stated in the figure legend (line 202-203).***

Fig 1 legend has been amended accordingly.

***Line 260-261, ‘in parallel’ should be moved to the beginning of the sentence for clarity.***
Done.

***Add immunofluorescence images of BARP and MISP co-expressing. Line 368 and S17Fig clearly states that BARP and MISP are co-expressed, but the IF images shown (Figure 2) either show one or the other on a given cell at a given time, not true co-expression.***
We apologise as we realize this statement is misleading. Unfortunately, co-staining using both antibodies can’t be performed because they were both made in rabbit as explained above. The reviewer is correct in pointing out that this is not a true co-expression.

***Add the ladder to the western blots images.***
Ladders have been added to all western blots and Coomassie-stained gels in the corresponding supplementary figures (S16 and S23 Fig).

***Line 495, change ‘developed’ to ‘developing’***

‘developed’ has been replaced for ‘developing’.

***Line 496, change ‘either’ to ‘both’ and add an ‘s’ to ‘antigen’***

Suggested changes have been introduced.

***Line 504, change ‘neither’ to ‘either’ and ‘nor’ to ‘or’***
All suggested changes have been introduced in the revised version.

***While hidden in the supplements, we think the ‘small’ but significant reduction of salivary gland infection in the MISP knock out cell lines is very compelling. It would be interesting to look at different numbers of epimastigotes vs. metacyclics in the salivary glands using these knock out lines (as S16 Fig H only looks at general salivary gland infection – not type or numbers) or to see if the knock out metacyclics have any other differences in vitro (rather than just parasitemia in the mouse) but these would again take a lot of time and are not essential to this paper. Instead, they would be nice follow up experiments done at a later date.***

These are all great suggestions, and we thank the reviewer for acknowledging the challenges behind performing these experiments. In the repeated infection experiment using the MISP null mutant and add back, we didn’t see a particular difference in terms of morphologies or number of epimastigotes and pre-metacyclics in saliva. We did not analyse the infected glands because the infection rates were low in all groups, and we needed to maximise getting the highest number possible of “free swimming” parasites in saliva after spinning down the whole gland (and potentially damaging the tissue).

**Reviewer #4:**

***1) Figure 1C: the purple for the repeats and for the MISP-B is not intuitive, maybe change the color of either one to make a differentiation.***

Done.

***2) Figure 2B, 2C, 2E: the image space could be used better, some of the trypanosomes are pretty small while most of the area is black (done better in 2D)***

We respectfully disagree with the reviewer on this. We believe the individual immunostaining images in these figures are clear enough for comparison and identification of different cell types, morphologies, and position of both kDNA and nucleus.

***3) Figure 2B, 2C, 2E: a negative control with PCF, BSF or MG stage is necessary for showing antibody specificity (similar to Fig S6).***

We have now included these controls in S7 Fig.

***4) Figure 2A: significance from two technical replicates is not acceptable, three if possible biological replicates should be used. Why is it a one-sided t-test? Did the authors expect to just see upregulation to begin with?***

As explained above, we have now modified the Fig 2 legend accordingly.

***5) Figure 3b: the inlet plot should be covering the size range of 10 – 200 kD to judge the purity of the fraction, otherwise this is not very informative.***

Original gels covering the whole MW ladder are now in the supplementary information section (S16 and S23 Fig).

***6) Figure S4: Why is the normalization done to tert and not a universal expressed transcript?***

Transcript normalization using *T. brucei tert* has worked well for us following the published protocol in 1) Brenndorfer M, Boshart M. 2010; Mol Biochem Parasitol. doi: 10.1016/j.molbiopara.2010.03.007, and 2) Dewar S, Sienkiewicz N, Ong HB, Wall RJ, Horn D, Fairlamb AH. 2016. J Biol Chem. doi: 10.1074/jbc.M116.750422. Brenndorfer M, Boshart M. 2010 found *tert* to be the best performing housekeeping gene for *T. brucei* in comparison to other commonly used genes such as *GAPDH*, *tubulin*, *actin* or *18S*.

***7) Material and Methods: line 757 there is an alkylation step missing, otherwise carboxyamidomethylation as a fixed modification in MS/MS search does not make sense.***

We thank the reviewer for pointing out this omission of the alkylation step and apologize. Following the reduction of disulfide bonds (5 mM dithiothreitol for 15 min at 50°C), cysteine residues were alkylated with freshly prepared 10 mM iodoacetamide in 200 μl 100 mM Tris-HCl, pH 7.0, plus 8 M urea, for 30 min at room temperature (RT), protected from light.

We have now included a brief description of the trypsin digestion protocol in the revised *nLC-high resolution-MS/MS proteomic analysis* in the Methods section.

***8) MM: line 764, did you really search for acetylation on ANY amino acid as a variable modification? This does not make any sense chemically and is also not a preset standard in the field. I guess this just got wrongly described here, otherwise I would be curious for an explanation of this setting.***

We apologize for the mistake. Acetylation was a variable modification only searched on lysine residues (epsilon-amino lysine acetylation) and N-terminal amino acids (Nt-acetylation). This has been corrected in the revised manuscript, MM section.

***9) MM line 769ff, for all downloaded databases please indicate the triTrypDB database version instead of the downloaded date. They do versioning and this is easier to reproduce.***
We have now included the version/release number of several databases (lns 828-832).

***10) The original mass spec data should be uploaded to a repository like PRIDE.***

We have deposited the raw files and processed proteomic data on PRIDE; the accession number has been in the corresponding materials and methods section, and can be accessed using the following account details: Username: [reviewer_pxd039684@ebi.ac.uk](mailto:reviewer_pxd039684@ebi.ac.uk)

Password: 0hBp6EV5
